# Genomics‐Driven Discovery of NO‐Donating Diazeniumdiolate Siderophores in Diverse Plant‐Associated Bacteria

**DOI:** 10.1002/anie.201906326

**Published:** 2019-08-05

**Authors:** Ron Hermenau, Jule L. Mehl, Keishi Ishida, Benjamin Dose, Sacha J. Pidot, Timothy P. Stinear, Christian Hertweck

**Affiliations:** ^1^ Department of Biomolecular Chemistry Leibniz Institute for Natural Product Chemistry and Infection Biology (HKI) Beutenbergstrasse 11a 07745 Jena Germany; ^2^ Department of Microbiology and Immunology at the Doherty Institute University of Melbourne Melbourne VIC 3000 Australia; ^3^ Natural Product Chemistry Faculty of Biological Sciences Friedrich Schiller University Jena 07743 Jena Germany

**Keywords:** Burkholderia, diazeniumdiolate, nitric oxide, non-ribosomal peptide, siderophores

## Abstract

Siderophores are key players in bacteria–host interactions, with the main function to provide soluble iron for their producers. Gramibactin from rhizosphere bacteria expands siderophore function and diversity as it delivers iron to the host plant and features an unusual diazeniumdiolate moiety for iron chelation. By mutational analysis of the grb gene cluster, we identified genes (grbD and grbE) necessary for diazeniumdiolate formation. Genome mining using a GrbD‐based network revealed a broad range of orthologous gene clusters in mainly plant‐associated Burkholderia/Paraburkholderia species. Two new types of diazeniumdiolate siderophores, megapolibactins and plantaribactin were fully characterized. In vitro assays and in vivo monitoring experiments revealed that the iron chelators also liberate nitric oxide (NO) in plant roots. This finding is important since NO donors are considered as biofertilizers that maintain iron homeostasis and increase overall plant fitness.

Most organisms on earth require iron to maintain essential functions of their metabolism.^1^ Despite its high abundance in natural environments, iron is only scarcely available due to its low solubility under aerobic conditions. To cope with the limited supply of iron, microorganisms and some plants developed strategies that rely on the secretion of siderophores, small organic molecules that exhibit a high binding affinity towards iron(III).^2^ Thus, upon chelation the metal is mobilized and can be taken up for further usage. The presence of siderophores can influence microbe–host interactions in various ways. While many siderophores serve as virulence factors for pathogens,^3^ others may enhance plant growth and protect the host from pathogens.^4^ Numerous siderophores have various non‐classical biological functions,^5^ for example, as signaling molecules regulating virulence factor production,^6^ as mediators of mutualistic interactions,^7^ and as agents that may interfere with bacterial quorum sensing,^8^ swarming,^9^ and development.^10^ This wide range of activities, together with their omnipresence in ecological systems, has led to the discovery of more than 270 structurally characterized siderophores.^2^ Despite the high number of compounds, the variety of ligand systems in microbial siderophores is almost exclusively limited to catecholates, hydroxamates, and α‐hydroxy carboxylates.^2^, ^11^

With the recent discovery of gramibactin (**1**, Figure 1), this list was expanded with novel diazeniumdiolate (*N*‐nitroso‐*N*‐hydroxylamine) chelators found in two graminine (Gra, **2**) moieties of the cyclopeptide.^12^ With only a few other diazeniumdiolate‐bearing natural products described so far, **1** was the first to be characterized as a siderophore. More importantly, the siderophore produced by rhizosphere bacteria (*Paraburkholderia graminis*) increases iron content and thus chlorophyll production in corn plants by 50 %, indicating an important role of the siderophore in the plant–microbe interaction.^12^ Yet, it remained to be clarified whether such unusual siderophores are more widely distributed, how they are biosynthesized, and if they have additional functions. Here we shed more light on the molecular basis for the biosynthesis of the diazeniumdiolate siderophores and use this knowledge in a systematic approach to identify novel diazeniumdiolate‐siderophores in a broad range of bacteria. We also show that the chelators play a second role as NO‐donors.

**Figure 1:**
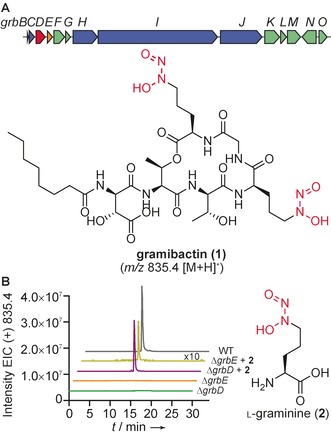
Identification of two essential genes for graminine biosynthesis. A) Biosynthetic gene cluster and structure of gramibactin (**1**). B) Extracted ion chromatograms (*m*/*z* [*M*+H]^+^ of **1**) of culture extracts of wild type (WT), *P*. *graminis* Δ*grbD*, *and* Δ*grbE* mutants, and chemically complemented mutant cultures.

To mine bacterial genomes for putative diazeniumdiolate siderophore biosynthetic pathway genes, we first aimed at identifying genes that are essential for the biosynthesis of *N*‐nitrosylated amino acid building blocks, such as graminine. Therefore, we analyzed and inactivated candidate genes in the *grb* locus in the genome of *P. graminis* (Figure 1 A).^12^ In addition to genes coding for the non‐ribosomal peptide assembly line (non‐ribosomal peptide synthetase, NRPS, and accessory enzymes, thioesterase and MbtH‐like protein) and transporter and receptor for siderophore shuttling, we detected two genes (*grbD* and *grbE*) with unknown functions. To test their involvement in Gra biosynthesis, we inserted a chloramphenicol resistance cassette in the individual genes by homologous recombination. LC‐MS analysis of culture supernatants of the Δ*grbD* and Δ*grbE* mutants revealed that the absence of GrbD and GrbE fully abrogates gramibactin production. To exclude the possibility of polar effects caused by the gene replacements, we chemically complemented the mutants with synthetic l‐graminine (Gra) that would be activated by the respective adenylation domains and transformed into the enantiomeric building block by the adjacent epimerase domains. We found that the supplement restored gramibactin production in both mutants (Figure 1 B), Thus, *grbD* and *grbE* were proven essential for graminine biosynthesis. This finding is intriguing since the closest characterized homologue of GrbD, SznF, has just been shown to mediate the nitrosylation of a urea moiety in streptozotocin biosynthesis.^13^ Whereas GrbE may play a role in the formation of the hydroxylamine, GrbD apparently mediates N−N‐bond formation. This strategy is markedly different from the fragin biosynthetic pathway, where an AurF‐like enzyme has been implicated in nitroso formation.^14^

Our gene‐inactivation experiments proved to be the key to identifying novel producers of diazeniumdiolate siderophores. We first used the EFI‐EST tool to blast the amino‐acid sequence of GrbD against the database of non‐redundant protein sequences (UniProt). We subjected these candidates to a genome neighborhood analysis (EFI‐GNT) and selected hits adjacent to homologues of GrbE, either as distinct proteins or as part of multifunctional enzymes (for example, fused to putative MbtH‐like proteins or NRPS) and excluded those with gene clusters interrupted by contig borders. Curating the list yielded 37 gene clusters, 22 of which clustered in a sequence similarity network (SSN) (Figure 2).

**Figure 2:**
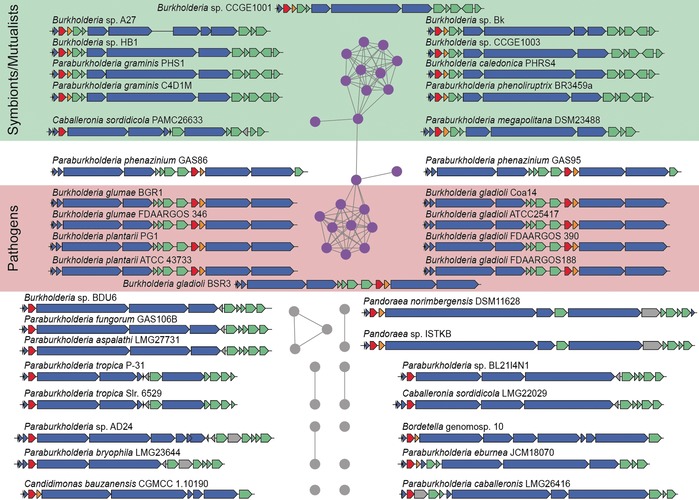
Genome mining for tentative diazeniumdiolate siderophore biosynthesis gene clusters. Sequence similarity networks based on GrbD with nodes indicating homologous genes in distinct subgroups that can be also classified according to the ecological context of the respective strains. Homologues of *grbD* are shown in red, *grbE* in orange, putative transporter and receptor related genes in green, NRPS and accessory protein encoding genes in blue.

Interestingly, all bacterial strains harboring these biosynthetic gene clusters belong to the family of *Burkholderiaceae* and are clearly divided into two major groups. The first group contains a range of plant‐associated, mutualistic *Burkholderia* and *Paraburkholderia* strains. These include rhizosphere bacteria (*P. graminis*), *P*. *caledonica*, which was also isolated from the rhizosphere^15^ and disease‐suppressing soils of an agricultural sugar‐beet field,^16^ and the moss‐associated *P. megapolitana*.^17^ The architectures of the gene clusters are similar to the *grb* gene locus. The second group is represented by mainly plant‐pathogenic *Burkholderia* species, such as *B. gladioli*, *B. glumae*, and *B. plantarii*. A similar cluster architecture was also found in two soil isolates of *P. phenazinium*. Since all detected clusters harbor genes for siderophore transporter and receptor proteins, the encoded metabolites likely function as iron shuttles. The composition of the peptide backbone was predicted by in silico analysis of the NRPS architecture and the adenylation domain specificities.

To test their potential to produce diazeniumdiolate‐containing siderophores, we cultured a selection of these strains in the same medium that triggered gramibactin production in *P*. *graminis*.^12^ Supernatants were extracted with XAD16N and analyzed by LC‐MS/MS applying untargeted fragmentation of the most abundant ions in each scan. With this approach we screened for parent ions producing the characteristic *M*‐30 fragment ions (‐NO), resulting from cleavage of the diazeniumdiolate moieties. First, as proof of concept, we investigated a species (*P*. *caledonica*) isolated from the rhizosphere^15^ and disease‐suppressing soils of an agricultural sugar‐beet field.^16^ *P. caledonica* harbors a *grb*‐like gene cluster and is therefore a potential gramibactin producer. HPLC‐MS profiling of the culture and comparison with an authentic sample revealed that this strain produces gramibactin. Furthermore, we detected a compound with *m*/*z* 853.3898 [*M*+H]^+^ and a fragmentation pattern that corresponds to a linearized gramibactin congener, named gramibactin B (**3**) (Figure 3 A and Supporting Information, Figures S3–4).

**Figure 3:**
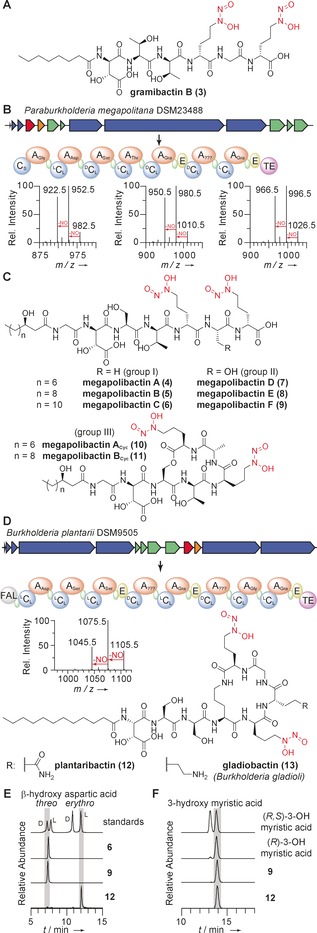
Discovery of novel diazeniumdiolate siderophores. A) Structure of gramibactin B, produced by *P. caledonica*. B) Biosynthetic gene cluster and deduced NRPS assembly line for megapolibactins, together with predicted building blocks. A: adenylation domain; C: condensation domain; E: epimerase domain; TE: thioesterase domain; green: thiolation domains. MSMS spectra showing characteristic losses of NO upon fragmentation of megapolibactin B, C, and F. C) Chemical structures of megapolibactins. D) Biosynthetic gene cluster and deduced NRPS for plantaribactin, together with predicted building blocks. MSMS spectra showing characteristic losses of NO upon fragmentation of plantaribactin. E) EIC traces of Marfey's derivatives to assign absolute configurations of β‐hydroxy aspartates in **6**, **9**, and **12**. F) EIC traces of Mosher derivatives of 3‐hydroxy myristic acid from **9** and **12**.

Culture supernatants from *P*. *megapolitana* showed a high number of different parent ions with *M*‐30 fragments (Figure 3 B). Furthermore, we detected corresponding iron complexes [*M*‐2H+Fe]^+^ with the characteristic *M*‐2 iron isotope peak. The extract of an up‐scaled culture of *P*. *megapolitana* was subjected to open column chromatography on Sephadex LH20, and preparative HPLC yielded six compounds (**4**–**9**), named megapolibactins A–F, with five of them in amounts that enabled their full characterization. ^1^H‐^1^H‐COSY, HMBC, TOCSY, and NOESY NMR correlations elucidated the peptide structures, and additional MS/MS, IR, and UV/Vis measurements confirmed the presence of the diazeniumdiolate moieties. The amino acid building blocks were identified as glycine, β‐hydroxyaspartic acid, serine, threonine, and two graminine moieties. While three of the compounds (group I) contain an additional alanine between both graminine residues, the other two (group II) bear serine residues instead. The third subgroup (III) of megapolibactins consists of two cyclic megapolibactins. Based on MS/MS fragmentation patterns, we concluded that these compounds are lactone congeners in which the C‐terminus is condensed with the serine hydroxyl group (Figure 3 C). By Marfey's analysis, we elucidated the absolute configuration of amino acids in megapolibactin C as d‐*threo*‐3‐hydroxyaspartic acid, l‐serine, d‐*allo*‐threonine, d‐graminine, and l‐alanine. The results of the same analysis for megapolibactin F differed only in the loss of a signal corresponding to l‐alanine, indicating that both serine residues in group II are l‐configured. The identified NRPS modules are colinear with the elucidated peptide backbones and in agreement with the stereochemical assignment. As suggested by the presence of a C_Starter_ domain, the N‐termini of the peptides are acylated. NMR spectroscopic analyses, HRMS and MS/MS experiments revealed that the C_Starter_ domain incorporates 3‐hydroxydecanoic acid, 3‐hydroxydodecanoic acid, and 3‐hydroxytetradecanoic acid. Derivatization with Mosher's acid chloride and LC‐MS analysis of fatty acids incorporated in megapolibactin C and F identified the 3‐hydroxytetradecanoic acids to be *R*‐configured (Figure 3 F).

From the culture extract of the representative of the second large group, *B. plantarii*, we isolated a compound named plantaribactin (**12**) with *m*/*z* 1105.5484 [*M*+H]^+^ that matched with the mass range of the predicted peptide assembled by the *plb* NRPS. The diagnostic ‐NO losses observed in HR‐MS/MS fragmentation experiments indicated the presence of diazeniumdiolate moieties (Figure 3 D). We also detected a second species with *m*/*z* 1158.4608 [*M*‐2H+Fe]^+^, which is typical for siderophores. 1D and 2D NMR spectra allowed the elucidation of the peptide backbone. The presence of an amide proton resonating at *δ*_H_=7.64 ppm adjacent to a methylene unit indicated a lactam bond formed by the C‐terminus and the δ‐amino group of ornithine. Based on NOESY and HMBC NMR experiments, we elucidated the connections of the identified amino acids. A modified Marfey analysis revealed that plantaribactin consists of l‐*erythro*‐3‐hydroxyaspartic acid, d‐serine, l‐serine, l‐ornithine, l‐glutamine, and d‐graminine (Figure 3 D). To pinpoint the position of the two enantiomeric Ser moieties, we supplemented *B. plantarii* cultures with deuterated l‐serine and compared HR‐MS/MS spectra of native plantaribactin with the spectra obtained of the labeled compound (Supporting Information, Figure S6). Thus, we determined the l‐Ser‐d‐Ser sequence, which is in full agreement with the NRPS domain architecture showing an epimerization domain in module 3. Finally, the size of the N‐terminal fatty acid was determined by HRMS and GC analysis (Supporting Information, Figure S7). Interruption of *plbD* by a chloramphenicol resistance cassette abolished plantaribactin biosynthesis, clearly linking plantaribactin production to the identified gene cluster (Supporting Information, Figure S9). The high similarities of the gene clusters of *B. plantarii*, *B. glumae*, and *B. gladioli* implied that the encoded pathways are alike or even identical. Indeed, LC‐HRMS‐based metabolic profiling of a *B. glumae* strain revealed that it produces the same compound (Supporting Information, Figure S10 A). In extracts from culture supernatants of three different *B. gladioli* strains, a congener of plantaribactin was detected (Supporting Information, Figure S10 B). Based on MS/MS fragmentation, we concluded that glutamine is replaced by lysine (Supporting Information, Figure S10 C). According to the bacterial source we named this new compound gladiobactin (**13**).

It is intriguing that plant‐associated bacteria employ such unusual chelating groups for iron complexation. Thus, we wondered whether diazeniumdiolate siderophores could have an additional function. Their structures and the MS fragmentation suggest that they could serve as nitric oxide (NO) donors. Notably, NO is a versatile signaling compound in animals and plants, and NO‐donors thus play an important role in pharmacy and are considered to have a high potential in agriculture.^18^ Nitric oxide can be enzymatically released from diazeniumdiolate residues by means of horseradish peroxidase and hydrogen peroxide.^19^ Notably, peroxidases and reactive oxygen species are highly abundant in plant roots, especially in the root elongation zone, as they are required for growth.^20^

To probe the proposed enzymatic NO release in vitro, we extracted soluble proteins from ground root tissue of hydroponically grown corn plants into phosphate buffer. To monitor the liberation of NO in the in vitro enzyme assay, we used 2,3‐diaminonaphthalene (DAN) as a reporter. DAN reacts with N_2_O_3_ generated from nitric oxide and molecular oxygen and forms a highly fluorescent 2,3‐naphthotriazole that can be detected fluorometrically (Figure 4 A).^21^ In addition to gramibactin we tested megapolibactin B and plantaribactin, as well as graminine. Increased fluorescence, and therefore nitric oxide release, could be observed for all peptides and the free amino acid (Figure 4 B–D). Notably, NO release was not detected when adding the synthetic, isosteric hydroxamate analogue instead of the diazeniumdiolate (Figure 4 E), which clearly demonstrates that NO originates from the administered diazeniumdiolate probe. Furthermore, these results revealed that both, reactive oxygen species and proteins from the plant root are necessary for releasing NO.

**Figure 4:**
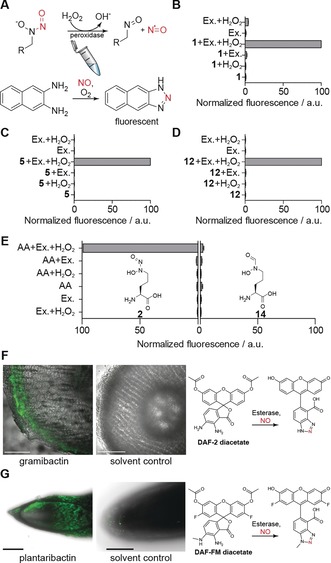
Nitric oxide release from diazeniumdiolate‐containing compounds in vitro and in vivo. A) Proposed reaction mechanism of enzymatic nitric oxide release. B–D) Normalized fluorescence observed after treating **1**, **5**, and **12** with extracted corn root proteins (Ex.) and hydrogen peroxide in the presence of 2,3‐diaminonaphthalene. E) Comparison of NO release activity of **2** and its hydroxamate analogue **14**. F) Confocal laser scanning microscopy for in vivo imaging of NO release in cut corn roots after treatment with **1** using DAF2‐DA. G) Spinning disc confocal microscopy for in vivo imaging of NO release from **12** with DAF‐FM DA in hydroponically grown rice roots. Pictures represent summed intensity over a *z*‐stack measured through the root tip. Fluorescence indicates presence of nitric oxide. Scale bars equivalent to 200 μm.

To verify the postulated NO release in planta, we treated intact corn seedlings with diaminofluoresceine‐2 diacetate (DAF‐2 DA), a cell‐permeable probe that captures NO with formation of a highly fluorescent triazole. Investigating sections of root tips by fluorescence microscopy we detected the strongest fluorescence, and thus highest concentration of NO, in the outer root sections (Figure 4 F). To test the universality of this process, we also evaluated the capability of plantaribactin to release NO in rice plant roots. Instead of measuring cut root sections, we employed spinning disc confocal microscopy (SDCM). In accord with the results obtained for corn plant roots, we detected strong fluorescence, indicating the presence of elevated NO levels, in outer plant tissue of roots treated with plantaribactin and stained with the cell‐permeable probe DAF‐FM DA (Figure 4 G). Furthermore, by MS analysis of rice plants treated with plantaribactin, we detected an increase of a species that corresponds to a congener lacking nitric oxide (Supporting Information, Figure S13). These results unequivocally show that diazeniumdiolate siderophores are viable NO donors in planta.

The release of nitric oxide by a metabolite from plant‐associated and rhizosphere bacteria is remarkable because NO represents an important plant hormone regulating many different functions in the plant, including growth, defense mechanisms, and formation of symbioses.^22^ In the context of siderophores it is remarkable that NO also plays a key role in iron homeostasis and improves internal iron availability, likely by formation of iron‐nitrosyl complexes.^23^ From a translational point of view it is noteworthy that exogenously supplied NO‐donors were found to increase plant fitness, root growth, and tolerance towards stress.^24^ In the light of these beneficial traits of NO from the host perspective, it is intriguing that NO‐releasing metabolites are also produced by plant pathogens. Yet, NO might also support microbial pathogenicity, by promoting root growth at the site of infection to further increase colonizable areas. Furthermore, for most pathogens in vivo survival crucially depends on the resistance against oxidative stress from the host's immune response. In several *Bacillus* strains, this resistance is conferred by nitric oxide.^25^

These findings are important as they not only provide the first insight into the genetic basis for diazeniumdiolate formation in gramibactin biosynthesis but also show that diazeniumdiolate siderophore pathways are widespread in bacteria that are associated with plants or are found in disease‐suppressing soils. Furthermore, NO‐donating siderophores are a fresh addition to the growing body of knowledge on multifunctional chelators. Beyond ecological studies and evolutionary considerations, our work may inspire the use of NO‐donating siderophores or the producer strains in agriculture.^26^

## Conflict of interest

The authors declare no conflict of interest.

## Supporting information

As a service to our authors and readers, this journal provides supporting information supplied by the authors. Such materials are peer reviewed and may be re‐organized for online delivery, but are not copy‐edited or typeset. Technical support issues arising from supporting information (other than missing files) should be addressed to the authors.

Supplementary
