# Supplementary material for: Genomics‐Driven Discovery of NO‐Donating Diazeniumdiolate Siderophores in Diverse Plant‐Associated Bacteria
Source: Angew Chem Int Ed Engl. 2019 Aug 5;58(37):13024–9. doi: 10.1002/anie.201906326 (PMC6771848; doi:10.1002/anie.201906326)
Supplement: Supplementary file 1 — Supplementary [file ANIE-58-13024-s001.pdf]

Angewandte Chemie
Eine Zeitschrift der Gesellschaft Deutscher Chemiker

Supporting Information

Genomics-Driven Discovery of NO-Donating Diazeniumdiolate Siderophores in Diverse Plant-Associated Bacteria

*Ron Hermenau, Jule L. Mehl, Keishi Ishida, Benjamin Dose, Sacha J. Pidot, Timothy P. Stinear, and Christian Hertweck**

anie_201906326_sm_miscellaneous_information.pdf

Table of Contents

List of Tables 4
General Analytical Procedures 5
General Cultivation Procedures....5
MM9 Media Preparation 5
General extraction procedure 5
Genome sequencing 5
Preparation of Knock-out Strains of *P. graminis* C4D1M 5
Chemical complementation of *P. graminis* knock out strains 6
Creation of structure similarity networks....6
Identification of gramibactin as a metabolite of *Paraburkholderia caledonica*....6
Isolation of megapolibactins 8
Isolation of plantaribactin....8
Marfeys Analysis 8
Elucidation of absolute configuration of 3-hydroxy fatty acids in megapolibactins 9
Elucidation of serine absolute configurations in plantaribactin 9
Identification of fatty acid in plantaribactin....10
Preparation of Knock-out Strain of *B. plantarii* DSM9509 11
Detection of plantaribactin in *B. glumae* cultures 13
Corn Cultivation Medium 15
Rice cultivation medium....15
Cultivation of Corn Plants 16
Cultivation of rice plants 16
In vitro NO-Release Assay 16
In planta NO Release Assay 16
Synthetic Procedures 18
- Synthesis of L-graminine....18
- Synthesis of N^2-Benzyloxycarbonyl-N^5-formyl-N^5-hydroxy-l-ornithine 19
- Synthesis of N^5-Formyl-N^5-hydroxy-l-ornithine 20

Structure Elucidation 21
- Megapolibactin A 21
- Megapolibactin B 23
- Megapolibactin C 25
- Megapolibactin D 27
- Megapolibactin E 27
- Megapolibactin F 29
- Megapolibactin A_{Cyc} 31
- Megapolibactin B_{Cyc} 31
- Plantaribactin 32

Analysis of putative biosynthetic gene clusters 34
Prediction of A-domain substrate specificity....37
Spectra of New Compounds....39
References 61

List of Figures

Figure S1. PCR confirmation of knock-out mutants of *P. graminis* 7
Figure S2. Detection of gramibactin in *P. caledonica* culture supernatants 8
Figure S3. MSMS spectra of gramibactin and gramibactin B 8
Figure S4. Deduced structure of gramibactin B....8
Figure S5. MSMS fragmentations of linear and cyclic megapolibactins showing characteristic NO losses 9
Figure S6. Elucidation of absolute configuration of serine residues in plantaribactin 11
Figure S7. GC analysis of the fatty acid incorporated in plantaribactin 12
Figure S8. PCR confirmation of knock-out mutants of *B. plantarii* 14
Figure S9. HPLC profiles of culture supernatants obtained from *B. plantarii* wt and knockout strains 14
Figure S10. Detection of plantaribactin and gladiobactin in cultures of *B.* glumae and *B.* gladioli 15
Figure S11. *In planta* nitric oxide imaging of corn root sections stained with DAF-2 DA....17
Figure S12. *In planta* nitric oxide imaging of rice roots stained with DAF-FM DA 18
Figure S13. Semiquantification of plantaribactin cleavage products in hydroponic solutions 18
Figure S14. Structure of megapolibactin A....22
Figure S15. MSMS fragmentation of megapolibactin A....22
Figure S16. Structure of megapolibactin B....24
Figure S17. MSMS fragmentation of megapolibactin B....24
Figure S18. Structure of megapolibactin C....26
Figure S19. MSMS fragmentation of megapolibactin C 26
Figure S20. Structure of megapolibactin D....28
Figure S21. Structure of megapolibactin E....28
Figure S22. MSMS fragmentation of megapolibactin E....28
Figure S23. Structure of megapolibactin F 30
Figure S24. MSMS fragmentation of megapolibactin F 30
Figure S25. Structure of megapolibactin A_{Cyc} 32
Figure S26. Structure of megapolibactin B_{Cyc}....32
Figure S27. Key COSY, HMBC, and NOESY correlations....33
Figure S28. Detected MSMS fragments....33
Figure S29. Putative biosynthetic gene cluster found in the genome of *P. caledonica*....35
Figure S30. Putative biosynthetic gene cluster found in the genome of *P. megapolitana.* 36
Figure S31. Putative biosynthetic gene cluster found in the genome of *B. plantarii.* 37
Figure S32. ^{1}H NMR Spectrum of L-graminine....40
Figure S33. IR spectrum and UV spectrum of megapolibactin A. 40
Figure S34. ^{1}H NMR spectrum of megapolibactin A 41
Figure S35. ^{1}H-^{1}H-COSY spectrum of megapolibactin A 41
Figure S36. HSQC spectrum of megapolibactin A 42
Figure S37. HMBC spectrum of megapolibactin A 42
Figure S38. ^{13}C NMR spectrum of megapolibactin A 43
Figure S39. NOESY spectrum of megapolibactin A 43
Figure S40. IR spectrum and UV spectrum of megapolibactin B. 44
Figure S41. ^{1}H NMR spectrum of megapolibactin B 44
Figure S42. ^{1}H-^{1}H-COSY spectrum of megapolibactin B 45
Figure S43. HSQC spectrum of megapolibactin B 45
Figure S44. HMBC spectrum of megapolibactin B. 46
Figure S45. ^{13}C NMR spectrum of megapolibactin B 46
Figure S46. NOESY spectrum of megapolibactin B. 47
Figure S47. IR spectrum and UV spectrum of megapolibactin C. 47
Figure S48. ^{1}H NMR spectrum of megapolibactin C 48
Figure S49. ^{1}H-^{1}H-COSY spectrum of megapolibactin C 48
Figure S50. HSQC spectrum of megapolibactin C. 49
Figure S51. HMBC spectrum of megapolibactin C....49
Figure S52. ^{13}C NMR spectrum of megapolibactin C 50
Figure S53. NOESY spectrum of megapolibactin C....50
Figure S54. IR spectrum and UV spectrum of megapolibactin E. 51
Figure S55. ^{1}H NMR spectrum of megapolibactin E. 51
Figure S56. ^{1}H-^{1}H-COSY spectrum of megapolibactin E 52
Figure S57. HSQC spectrum of megapolibactin E 52
Figure S58. HMBC spectrum of megapolibactin E. 53
Figure S59. ^{13}C NMR spectrum of megapolibactin E. 53

Figure S60. NOESY spectrum of megapolibactin E. 54
Figure S61. IR spectrum and UV spectrum of megapolibactin F. 54
Figure S62. ^{1}H NMR spectrum of megapolibactin F....................55
Figure S63. ^{1}H-^{1}H-COSY spectrum of megapolibactin F 55
Figure S64. HSQC spectrum of megapolibactin F 56
Figure S65. HMBC spectrum of megapolibactin F 56
Figure S66. ^{13}C NMR spectrum of megapolibactin F 57
Figure S67. NOESY spectrum of megapolibactin F 57
Figure S68. IR spectrum and UV spectrum of plantaribactin. 58
Figure S69. ^{1}H NMR spectrum of plantaribactin....................58
Figure S70. ^{1}H-^{1}H-COSY spectrum of plantaribactin 59
Figure S71. HSQC spectrum of plantaribactin. 59
Figure S72. HMBC spectrum of plantaribactin 60
Figure S73. ^{13}C NMR spectrum of plantaribactin 60
Figure S74. NOESY spectrum of plantaribactin 61
Figure S75. Comparison of MS/MS spectra of plantaribactin and gladiobactin 61

List of Tables

Table S1: Marfey's analysis of plantaribactin....................9
Table S2: Marfey's analysis of megapolibactins. 9
Table S3: Primers used in this study. Restriction sites are underlined. 12
Table S4: Plasmids used in this study. 12
Table S5: Bacterial strains used in this study. 12
Table S6: Components of corn cultivation medium. 15
Table S7: Components of rice cultivation medium....................15
Table S8: NMR data of megapolibactin A. 3-HDA: 3-Hydroxydecanoic acid....................22
Table S9: NMR data of megapolibactin B. 3-HDoA: 3-Hydroxydodecanoic acid....................24
Table S10: NMR data of megapolibactin C. 3-HtDA: 3-Hydroxytetradecanoic acid. 26
Table S11: NMR data of megapolibactin E. 3-HDoA: 3-Hydroxydodecanoic acid....................28
Table S12: NMR data of megapolibactin F. 3-HtDA: 3-Hydroxytetradecanoic acid....................30
Table S13: NMR data of plantaribactin. 33
Table S14. Annotation of deduced proteins from *cal* gene cluster....................34
Table S15. Annotation of deduced proteins from *meg* gene cluster. 35
Table S16. Annotation of deduced proteins from *plb* gene cluster. 36
Table S17. Predicted A-domain substrate specificity from the *cal* NRPS in the *P. caledonica* genome. 37
Table S18. Predicted A-domain substrate specificity from the *meg* NRPS in the *P. megapolitana* genome....................37
Table S19. Predicted A-domain substrate specificity from the *plb* NRPS in the *B. plantarii* genome....................37
Table S20. Predicted A-domain substrate specificity from the *plb* homologous NRPS in the *B. gladioli* pv. agaricicola genome.........38

General Analytical Procedures

NMR spectra were measured on Bruker Avance DRX 500 MHz or 600 MHz spectrometers (600 MHz with cryo probe) in DMSO-d_6. Spectra were referenced to the residual solvent peak. UV spectra were obtained on a Shimadzu UV-1800 spectrometer. A Jasco Fourier Transform Infrared Spectrometer 4100 was used to measure the infrared spectra using the ATR technique. For LC-MS measurements an Agilent 1100 coupled to a Bruker HCTultra PTM Discovery System with electrospray ion source using a Phenomenex Synergi 4u Hydro-RP 80A (250 × 4.6 mm, 4 µm) and an elution gradient [solvent A: H_2O + 0.1% HCOOH, solvent B: MeCN, gradient: 0.5% to 99.5% in 30 min, flow rate 1 mL min^{-1}] was used. LC-HRMS measurements were carried out on a Thermo Fisher Scientific QExactive Orbitrap with an electrospray ion source using a Thermo Accucore C_{18} column (100 × 2.1 mm; 2.6 µm) and an elution gradient [solvent A: H_2O + 0.1% HCOOH, solvent B: acetonitrile +0.1% HCOOH, gradient: 5% B for 1 min, 5% to 98% B in 15 min, 98% B for 3 min, flow rate: 0.2 mL min^{-1}, injection volume: 5 µL]. Gas-chromatographic measurements were executed on Thermo Trace GC Ultra equipped with CombiPAL autosampler and coupled with FID and Thermo Polaris Q electron impact ion trap mass spectrometer.GC conditions: column SGE BPX5 30 m × 0.25mm ID; carrier gas helium; split injection with split ratio 1:10 and injection volume 10 µL; 1.5mL/min carrier gas flow; temperature profile 0–1 min: 40°C, 1–3 min: heating up to 100°C (30°C/min), 3–28 min: heating up to 350°C (10°C/min).

General Cultivation Procedures

The medium used for production of gramibactin, plantaribactin, and megapolibactins was MM9.[1] For small cultures (2 mL), a pipette tip of cells from plate was added to the respective medium in culture tubes. The cultures were shaken at 150 rpm and at 30 °C for the selected time. For larger cultures (100 mL to 200 mL), a liquid pre-culture was prepared and the OD_{600} measured. Cultures were inoculated with an OD_{600} of 0.5 to 0.8. For large scale cultivations (4 L), pre-cultures were grown overnight and 2% *v/v* of the pre-culture was added to the medium. The cultures were shaken at 120 rpm and 30 °C for 3 days. Cultures of the knockout-strains *Paraburkholderia graminis ΔgrbD* and *ΔgrbE* and *Burkholderia plantarii ΔplbD* were always prepared with addition of 0.1% *v/v* of chloramphenicol (50 mM) in DMSO.

MM9 Media Preparation[2-3]

One liter of MM9 medium was prepared as follows. Solution A [350 g K_2HPO_4 and 100 g KH_2PO_4 dissolved in 1 L ddH_2O] and solution B [29.4 g NaCl, 50 g $(NH_4)_2SO_4$, 5 g $MgSO_4$ dissolved in 1 L ddH_2O] were prepared and autoclaved separately. 2 g of an amino acid mixture [2 g each of L-alanine, L-arginine, L-asparagine, L-aspartate, L-cysteine, L-glutamine, L-glutamate, glycine, L-isoleucine, L-proline, L-serine, L-threonine, L-tyrosine, L-valine, L-phenylalanine] were dissolved in 900 mL ddH_2O and autoclaved. After cooling down, 20 mL of each solution A and B were added. Additionally, 16.7 mL of L-leucine 100 mM, 5 mL of L-histidine 60 mM, 10 mL of L-lysine 100 mM, 10 mL of L-tryptophan 40 mM, 10 mL of L-methionine 40 mM, and 20 mL of glucose 50% (*w/v*) were added. Optionally, 1 mL of a trace element solution [per liter: 40 mg $ZnCl_2$, 200 mg $FeCl_3 \cdot 6\,H_2O$, 10 mg $CuCl_2 \cdot 2\,H_2O$, 10 mg $MnCl_2 \cdot 4\,H_2O$, 10 mg $Na_2B_4O_7 \cdot 10\,H_2O$, 10 mg $(NH_4)_6Mo_7O_{24} \cdot 4\,H_2O$] was added. For preparing agar plates, the amino acid mix was autoclaved after adding solution A and 15 g L^{-1} agar. Remaining supplements were added as described above.

General extraction procedure

For extraction of bacterial culture supernatants, Amberlite® XAD16N resin was used. First, cultures were centrifuged at 8000 rpm for 2-30 min at 20 °C and the supernatant was collected. XAD16N resin was prepared by washing it with acetone and methanol followed by equilibration with distilled water. The equilibrated resin (5% *w/v*) was added to the culture supernatant. The solution was stirred at room temperature for 2 h. The resin was filtered off, washed with water and eluted with methanol. From the methanol fraction, volatiles were removed and the residual aqueous solution was freeze-dried. The extract was dissolved in MeOH for LC-MS analysis or purified further.

Genome sequencing

Single molecule real-time (SMRT) sequencing was performed on a PacBio RS II instrument with subsequent analysis performed using SMRT Analysis v2.3.0.140936. The RS_HGAP_Assembly.3 algorithm was used for *de novo* assembly. Contigs were error corrected and polished using Illumina NextSeq reads that were mapped to each contig with Snippy v4.3.6 (github.com/tseemann/snippy). Illumina Nextera XT libraries were prepared and sequenced on an Illumina Nexseq platform, as per manufacturer's instructions. Assemblies were annotated with Prokka v1.13.1 (Seemann, 2014, Bioinformatics).

Preparation of Knock-out Strains of *P. graminis* C4D1M

Genomic DNA from *P. graminis* C4D1M was isolated as described previously.[1] A gene fragment containing *grbD* was amplified by PCR with the primer pairs Bg-UK-fw/Bg-UK-PacI and Bg-UK-KpnI/Bg-UK-NheI using DeepVent Polymerase (New England Biolabs) followed by Taq DNA Polymerase (New England Biolabs), respectively. The amplicons were purified using the illustra GFX PCR DNA and Gel Band Purification Kit (GE Healthcare Life Sciences) and cloned into pGEM T-easy vector (Promega), resulting in pGEM-Bg-UK-KO1 and pGEM-Bg-UK-KO2, respectively. The PCR product containing the chloramphenicol resistance gene, which was amplified from pACYC184[4] with the primers Cm-fw-KpnI and Cm-rv-PacI using DeepVent Polymerase, was purified using the above-mentioned procedure. The amplicon was cloned into pGEM T-easy vector and the resulting plasmid was restricted with *Pac*I and *Kpn*I. This chloramphenicol resistance cassette gene was cloned into the *Pac*I/*Spe*I restricted pGEM-Bg-UK-KO1 and *Kpn*I/*Nhe*I restricted pGEM-Bg-UK-KO2, generating pGEM-Δ*grbD*.

A gene fragment containing *grbE* was amplified by PCR with the primer pairs grbE-fw/grbE-PacI and grbE-KpnI/grbE-NheI using DeepVent Polymerase followed by Taq DNA Polymerase, respectively. The amplicons were purified using the above-mentioned

procedure and then cloned into pGEM T-easy vector, resulting pGEM-grbE-KO1 and pGEM-grbE-KO2, respectively. The above-mentioned chloramphenicol resistance cassette gene was cloned into the *Pac*I/*Spe*I restricted pGEM-grbE-KO1 and KpnI/NheI restricted pGEM-grbE-KO2, generating pGEM-Δ*grbE*.

P. graminis C4D1M was pre-cultured in LB medium at 30 °C. Overnight cultured cells were inoculated in LB medium (1/100 and 1/50 dilution) and cultured up to OD_{600}=0.4 to 0.6 at 30 °C. The culture broth was centrifuged at 2,500 × *g* and the supernatant was discarded. The cell pellet was resuspended in 300 mM sucrose solution and centrifuged again. After repeating this washing step twice, the washed cells were resuspended in 300 mM sucrose solution and subjected to electroporation (200 kV) with knockout plasmids (ca. 1−5 µg). Transformed cells were pre-cultured in LB broth (1 mL) for 4−6 h at 30 °C with shaking and then plated on either LB or nutrient agar plates with chloramphenicol (25 µg mL^{-1}). After 3 to 5 days, several positive colonies were observed and confirmed by colony PCR. Final PCR confirmation of knock-out mutants was performed using genomic DNA as a template, which was purified with The Wizard® Genomic DNA Purification Kit (Promega).

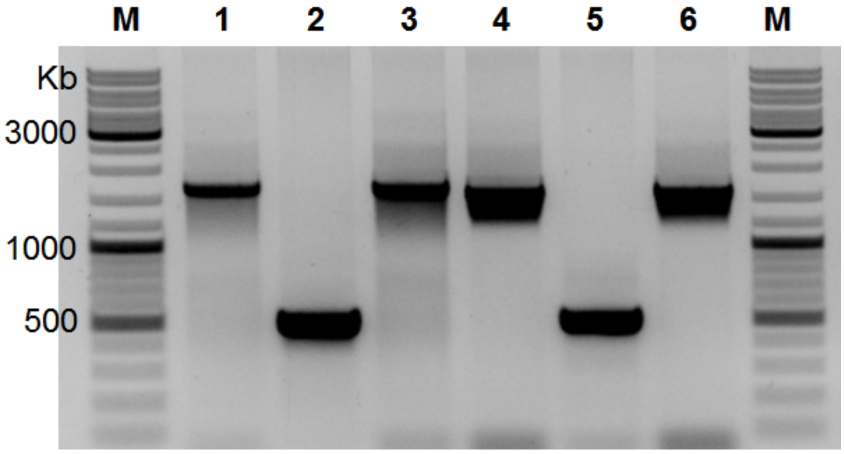

Figure S1. PCR confirmation of knock-out mutants of *P. graminis*. Template DNA: *P. graminis* Δ*grbD* (1), wild-type (2, 5), pGEM-Δ*grbD* (3), *P. graminis* Δ*grbE* (4), pGEM-Δ*grbE* (6). Primer pairs Bg-UK-fw3/Bg-UK-rv3 (lanes 1−3) and grbE-fw2/grbE-rv2 (lanes 4−6). M: marker. The estimated size of amplicons, lane 1; 1,623 bp, 2; 532 bp, 3; 1,623 bp, 4; 1,595 bp, 5; 554 bp, 6; 1,595 bp.

Chemical complementation of *P. graminis* knock out strains

In order to supplement cultures of the knockout-strains *P. graminis ΔgrbD* and *ΔgrbE* with synthetic graminine, the respective amount (final concentration 0.1 to 0.5 mg mL^{-1}) was dissolved 1 mL of MM9 medium. The solution was sterilized by filtration using 33 mm Ezee Syringe Filters (0.22 µm, PVDF) from Elkay and the filter was washed with 1 mL of MM9 medium. The sterile solution was mixed with 0.1% *v/v* of chloramphenicol (50 mM) in DMSO and added to the bacterial culture.

Creation of structure similarity networks (SSN)

The web tool EFI-EST[5] was used to create the sequence similarity network based on the amino acid sequence of GrbD using the standard settings. 210 was used as the alignment score to create the final networks. EFI-GNT was used to retrieve the genome neighbourhoods of each node in the network. Every node without a homologue of *grbE*, the query node, and nodes that had the respective cluster separated on different contigs were removed from the networks.

Identification of gramibactin as a metabolite of *Paraburkholderia caledonica*

P. caledonica DSM17062 cultures were prepared and extracted as described above. Gramibactin isolated from *P. graminis* was used as a standard for comparison (Figure S2). The hydrolysed and thus linear congener of gramibactin was identified using HRMSMS experiments (Figures S3-4).

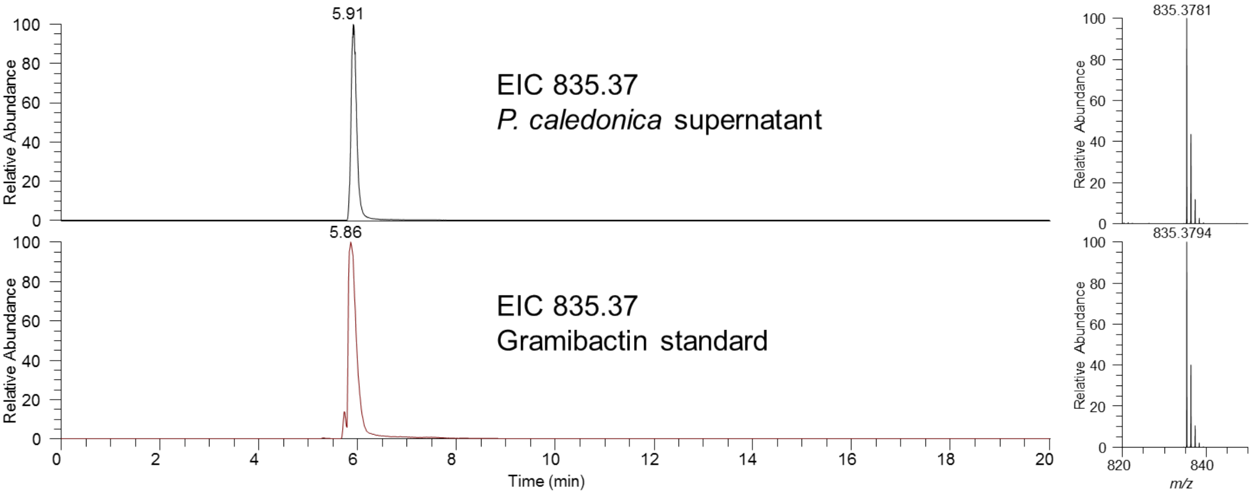

Figure S2. Detection of gramibactin in *P. caledonica* culture supernatants (positive ionisation mode).

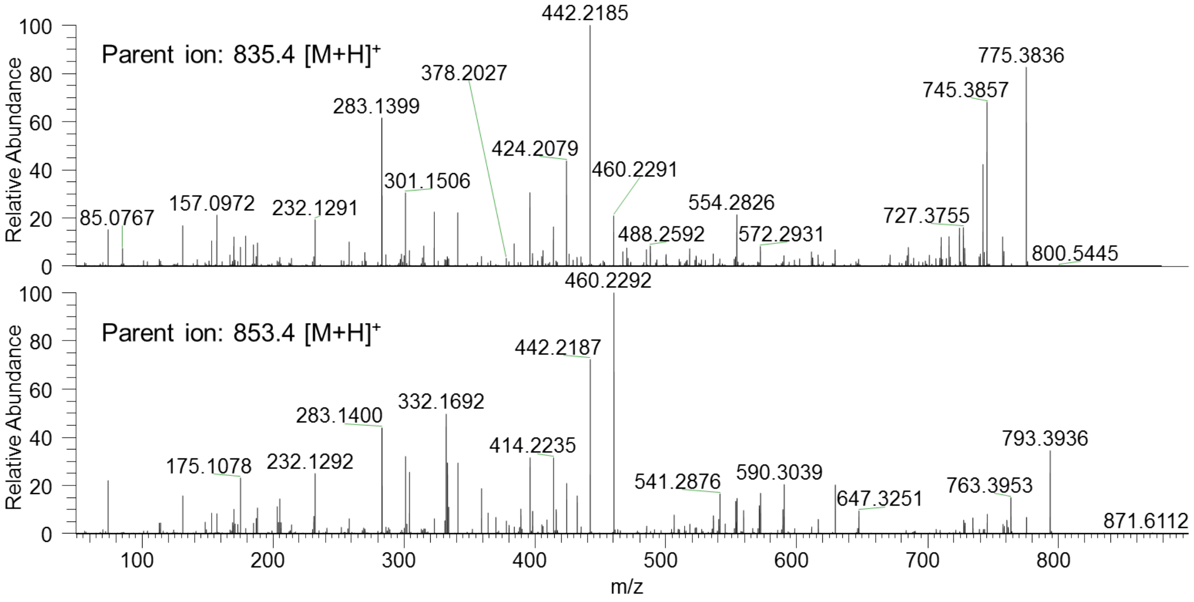

Figure S3. MSMS spectra of *m/z* 835.4 (gramibactin) and *m/z* 853.4 (gramibactin B).

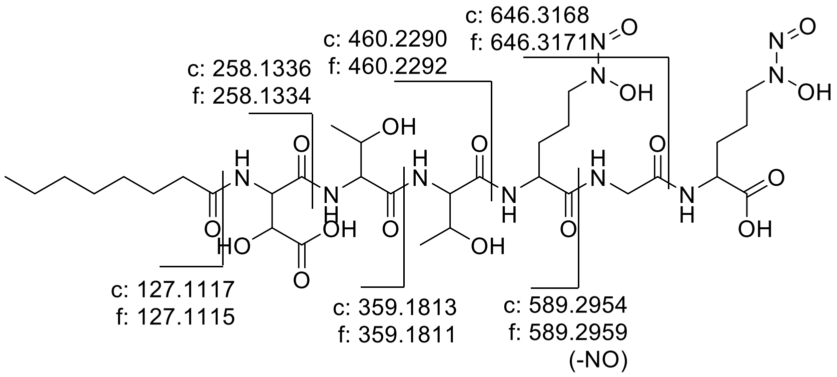

Figure S4. Deduced structure of *m/z* 853.4 corresponds to gramibactin B.

Isolation of megapolibactins

For the purification of megapolibactins, a 4 L-culture of *Paraburkholderia megapolitana* was prepared as described above. After three days, the cultures were extracted as described earlier. The crude extract was fractionated by open column chromatography with Sephadex® LH-20 beads in 3:2 water/methanol (v/v). Fractions containing megapolibactins were combined and evaporated to dryness. They were redissolved in 10% aqueous acetonitrile, centrifuged and the supernatant submitted to preparative HPLC (Synergy FusionRP 80A C18, 250 x 21.2 mm, 10 µm; 18 mL min^{-1}, A: H_2O with 0.1% TFA, B: 0.83% aqueous acetonitrile; 0-5 min: 15% B; 5–35 min: 15–100% B). Obtained fractions were lyophylized and yielded megapolibactins: 11.1 mg megapolibactin A, 20.4 mg megapolibactin B, 43.9 mg megapolibactin C, 0.97 mg megapolibactin D, 5.5 mg megapolibactin E, 15.0 mg megapolibactin F, trace amounts megapolibactin A_{Cyc}, 0.35 mg megapolibactin B_{Cyc}.

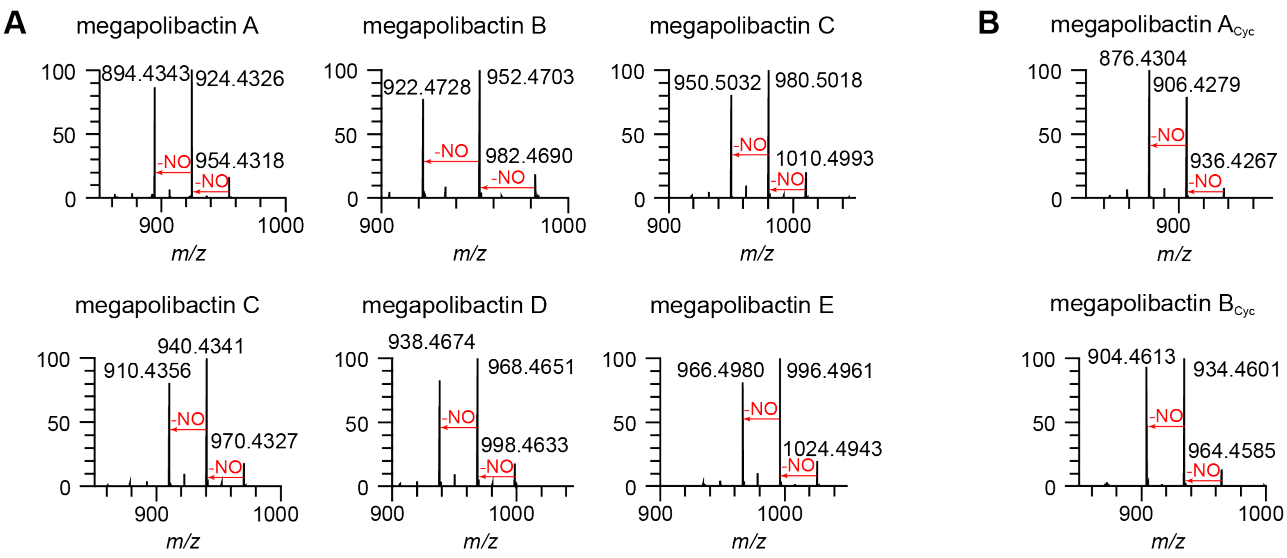

Figure S5. MSMS fragmentations of linear (A) and cyclic (B) megapolibactins showing characteristic NO losses.

Isolation of plantaribactin

B. plantarii was cultured in MM9 medium for 24 h at 28 °C and 120 rpm. Cultures were then centrifuged and the obtained supernatant was extracted as described above. Prepurification was achieved using a Sephadex® LH-20 column eluted with methanol. Fractions containing plantaribactin (based on LC-MS analysis) were pooled and submitted to preparative HPLC (Kromasil 100 C_{18}, 250 x 20 mm, 5 µm; 12 mL min^{-1}, A: H_2O with 0.1% TFA, B: 0.83% aqueous acetonitrile, 0-10 min 10% B, 10-30 min 10–75% B, 30-35 min 75-100% B, R_t = 30.5 min). Obtained fractions were lyophilized to yield a white powder (39 mg L^{-1}).

Marfeys Analysis

For analysis of the stereochemistry of synthesized amino acids or amino acids from isolated natural products, 1-fluoro-2,4-dinitrophenyl-5-L-alaninamide (L-FDAA; Marfey's reagent) was used. The amino acid of interest (0.5 to 1 mg) was dissolved in water (100 µL) and 50 µL of $NaHCO_3$ (1M) were added. L-FDAA was prepared as a 10 mg mL^{-1} solution in acetone shortly before use and 10 µL of this solution were added to the reaction mixture. At 40 °C, the solution was stirred for 1 h. Subsequently, 25 µL of 2 N HCl were added to quench the reaction. 25 µL methanol were added to the reaction mixture and an aliquot was analysed using LC-MS with a Thermo Accucore C_{18} column (100 × 2.1 mm; 2.6 µm) and an elution gradient [solvent A: H_2O + 0.1% HCOOH, solvent B: acetonitrile + 0.1% HCOOH, gradient: 10% B to 20% B in 10 min, 20% to 30% B in 20 min, flow rate: 0.2 mL min^{-1}, injection volume: 5 µL]. To cope with chromatographic instabilities, chromatograms were aligned to R_t of L-FDAA (16.88 min). D,L-*threo*-3-hydroxy aspartic acid and D,L-*erythro*-3-hydroxy aspartic acid were prepared according to literature.[6-7] Their elution order was already shown to be D→L.[8]

In order to analyse the amino acids of isolated natural products, the compounds (0.5 to 1 mg) were hydrolyzed in 20% DCl in D_2O (450 µL). The reaction mixture was stirred at 105 °C for 16 h. The solvents were removed *in vacuo*. And the residue was derivatized as described above. Synthetic L-graminine was hydrolysed in the same way before derivatization with L-FDAA.

Table S1: Marfey's analysis of plantaribactin.

	L-*erythro*-OH-Asp	L-Ser	D-Ser	L-Orn	D-Gra	L-Glu	Gly
**References**	12.07	11.43	11.85	7.74 & 9.30	19.41	14.17	14.38
**Plantaribactin hydrolysate**	12.06	11.44	11.86	8.15 & 9.52	19.31	14.10	14.32

Table S2: Marfey's analysis of megapolibactins.

	D-*threo*-OH-Asp	L-Ser	L-Ala	D-*allo*-Thr	D-Gra	Gly
**References**	7.25	11.43	16.09	13.86	19.41	14.38
**Megapolibactin C hydrolysate**	7.45	11.55	16.09	13.96	19.45	14.46
**Megapolibactin F hydrolysate**	7.36	11.50	---	13.94	19.39	14.43

Elucidation of absolute configuration of 3-hydroxy fatty acids in megapolibactins

Megapolibactin C and F were hydrolysed as described above. The obtained hydrolysate was extracted 4 times with 1 mL chloroform. The combined organic extracts were dried with sodium sulfate and evaporated to dryness. The residue was dissolved in 400 µL dry dichloromethane containing 0.2 mM dimethylaminopyridine. 5 µL *S*-MTPA-Cl was added and the reaction mixture was stirred for 4 h at room temperature. 1 mg *R*-3-hydroxy myristic acid and 1 mg *R,S*-3-hydroxy myristic acid were derivatized in the same way as reference compounds. Reaction mixtures were quenched with 500 µL water and the organic layer was separated. The aqueous phase was extracted 3 times with 800 µL dichloromethane and the combined organic phases were dried with sodium sulfate and evaporated to dryness. The residue was dissolved in methanol and analysed with LC-HRMS (Thermo Accucore C_{18} column (100 × 2.1 mm; 2.6 µm); elution gradient [solvent A: H_2O + 0.1% HCOOH, solvent B: acetonitrile + 0.1% HCOOH, gradient: 73% B for 20 min, flow rate: 0.2 mL min^{-1}, injection volume: 5 µL]). Both hydroxy fatty acids in megapolibactin C and F were found to be *R* configured, we suggest the same configuration for the remaining megapolibactins.

Elucidation of serine absolute configurations in plantaribactin

Marfey's analysis revealed the presence of two serine residues with different absolute configurations. Hydrolysis in DCl/D_2O did not lead to mass shifts in either of the peaks, ruling out that one of the configurations is an artefact of the hydrolysis. Bioinformatic analysis of the identified gene cluster suggests the second serine to be D-configured, due to the prediction of an epimerase domain within this module. To chemically proof this, we supplemented a 100 mL *B. plantarii* culture with19.8 mg 2,3,3-L-serine-d_3 and cultured it at 30°C for 48 h and extracted the culture as described above. The crude extract was submitted to LC-MSMS analysis. If an epimerization to D-serine takes place, the deuterium label in α-position would be exchanged by a proton, leading to a mass shift of 1. Parent ions with *M*+2 (deuterated D-serine), *M*+3 (deuterated L-serine), *M*+5 (deuterated D- and L-serine) were selected and fragmented. Fragment ions with 0, 1, and 2 serines were compared (Figure S6) with special interest in the isotopes of a fragment with only serine 2 remaining (*m/z* 645). The occurrence of a strong *m/z* 647 and a weak *m/z* 648 (caused by ^{13}C) obtained from a parent ion with two labelled serines incorporated indicates that serine 2 lost one deuterium due to epimerization. We conclude that serine 1 is L-configured and serine 2 D-configured. This is in accordance with the bioinformatic analysis of the gene cluster.

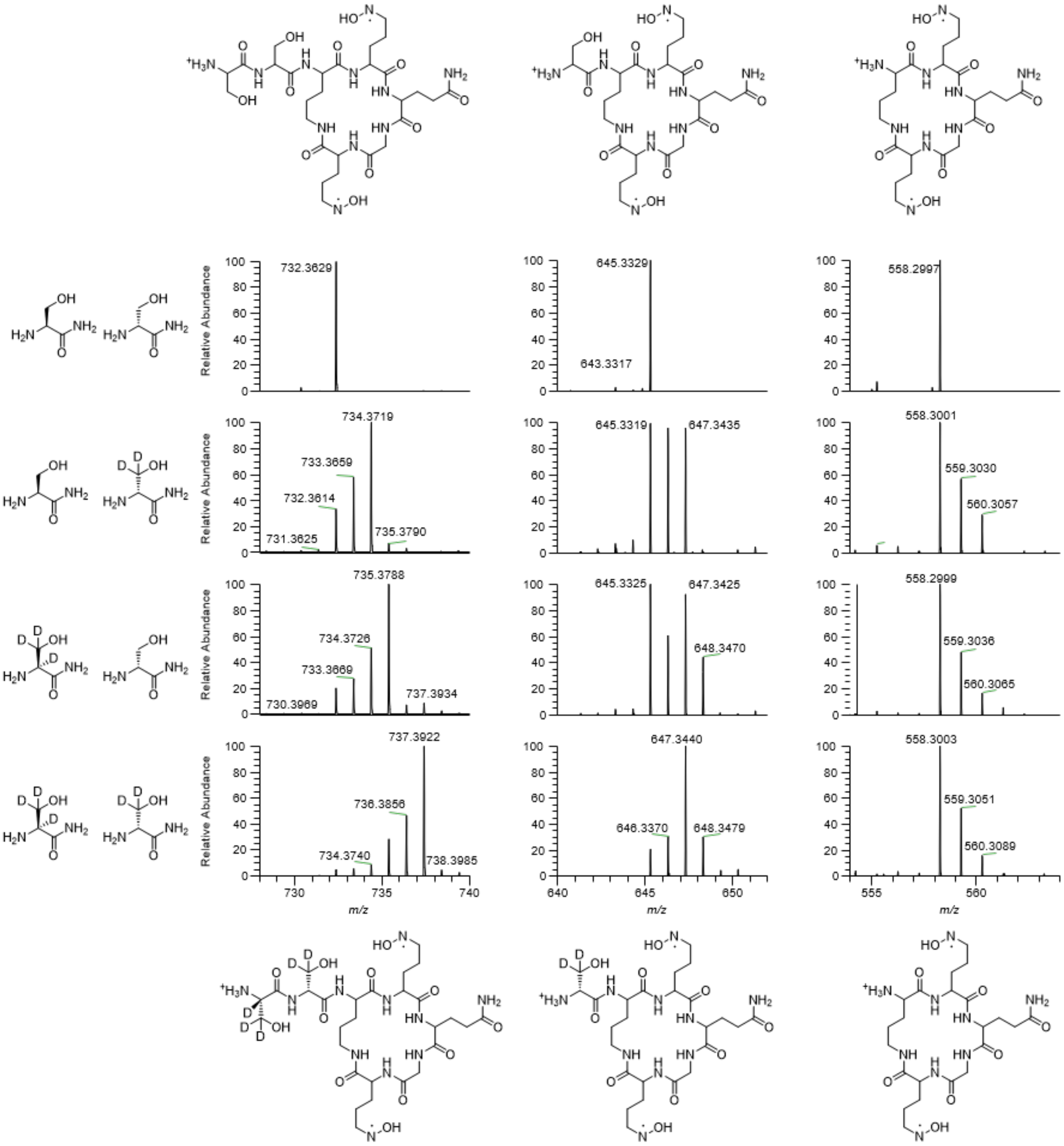

Figure S6. Elucidation of absolute configuration of serine residues in plantaribactin. Fragments with 0, 1, and 2 serines (columns) were obtained from parent ions indicating incorporation of 0, 1, or 2 deuterated serines (rows).

Identification of fatty acid in plantaribactin

Plantaribactin (1.8 mg) was hydrolysed as described above. The hydrolysate was extracted twice with 0.5 mL chloroform. Combined organic phases were dried with sodium sulfate and evaporated to dryness. The residue was dissolved in 0.5 mL anhydrous methanol and 45 µL TMS-diazomethane (2 M in hexane) was added and the resulting yellow solution was stirred 10 min at room temperature. 3 µL formic acid were added and the colourless solution evaporated to dryness after adding 1 mL toluene. The obtained residue was dissolved in chloroform and submitted to GC-MS analyses. Reference fatty acids (octanoic acid, decanoic acid, undecanoic acid, dodecanoic acid, tridecanoic acid, tetradecanoic acid) were derivatized in the same way. The fatty acid incorporated in plantaribactin was identified to be dodecanoic acid (Figure S7).

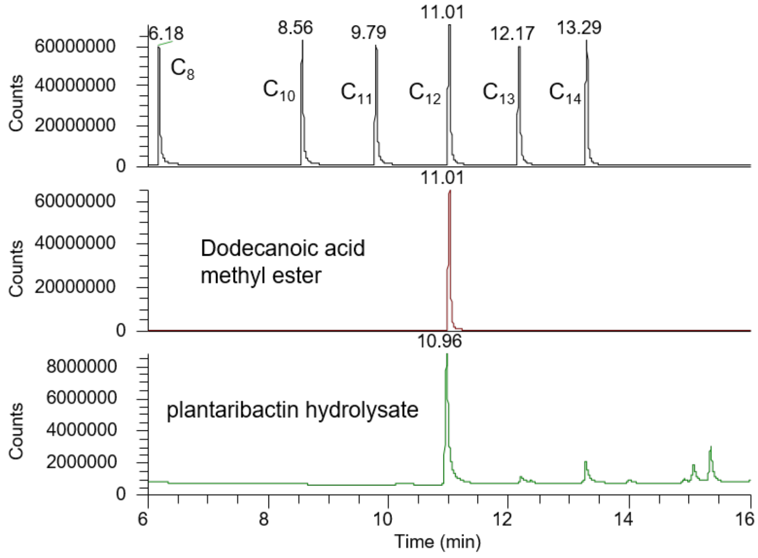

Figure S7. GC profile of reference fatty acid methyl esters, dodecanoic acid methyl ester and fatty acid methyl ester obtained from derivatizing a plantaribactin hydrolysate.

Preparation of Knock-out Strain of *B. plantarii* DSM9509

Genomic DNA of *B. plantarii* DSM9509 was purified using The Wizard® Genomic DNA Purification Kit. A gene fragment containing *plbD* was amplified by PCR with the primer pairs PlaNRPS-fw/PlaNRPS-PacI and PlaNRPS-KpnI/PlaNRPS-NheI using DeepVent Polymerase followed by Taq DNA Polymerase, respectively. The amplicons were purified with illustra GFX PCR DNA and Gel Band Purification Kit and then cloned into pGEM T-easy vector, resulting in pGEM-PlaNRPS-KO1 and pGEM-PlaNRPS-KO2, respectively. The above-mentioned chloramphenicol resistance gene was cloned into the PacI/SpeI restricted pGEM-PlaNRPS-KO1 and *Kpn*I/*Nhe*I-restricted pGEM-PlaNRPS-KO2, generating pGEM-ΔPlaNRPS.

Due to obtaining no double crossover mutant using this knock-out plasmid, pGEM-ΔPlaNRPS was restricted with *Psp*OMI/*Sbf*I and obtained knock-out gene fragment was cloned into *Psp*OMI/*Pst*I-restricted pGL42a_T251A[9], which possesses PheS as counter-selection marker for double crossover, generating pGL42a_T251A-ΔPlaNRPS.

B. plantarii DSM9509 was pre-cultured in LB medium overnight at 30 °C. Overnight cultures were inoculated in LB medium (1/100 and 1/50 dilution) and cultured up to OD_{600}=0.4 to 0.6 at 30 °C. The culture broth was centrifuged at 2,500 × *g* and the supernatant was removed. The cell pellet was resuspended in 300 mM sucrose solution and centrifuged again. After repeating this washing step twice, the washed cells were resuspended in 300 mM sucrose solution and subjected to electroporation (200 kV) with knockout plasmids (*ca*. 1−5 µg). Transformed cells were pre-cultured in LB broth (1 mL) for 4−6 h at 30 °C with shaking and then plated on LB agar plates with chloromphenicol (25 µg mL^{-1}). After 3 to 5 days, several positive colonies were observed and plated on MM9 agar plates with chloramphenicol (25 µg mL^{-1}) and 4-chloro D,L-phenylalanine (2 mg mL^{-1}). After confirmation by colony PCR (Figure S8A), selected colonies were further plated on new MM9 agar plates with chloramphenicol (25 µg mL^{-1}) and 4-chloro D,L-phenylalanine (2 mg mL^{-1}). Final PCR confirmation (Figure S8B) of knock-out mutants was performed using genomic DNA as a template, which was purified with The Wizard® Genomic DNA Purification Kit.

Table S3: Primers used in this study. Restriction sites are underlined.

Primer	Nucleotide sequence (5' to 3')	Source or reference
Bg-UK-fw	TTT CAT CGA CAC GGC GCG CG	This study
Bg-UK-PacI	GGTTTAATTAA ACT GCC AGT ACG CGT GCT GC	This study
Bg-UK-KpnI	GGTGGTACC CCT GTT GTA CGC ACT CGC GC	This study
Bg-UK-NheI	GGTGCTAGC GCA GCG TCG CGT ATG AAG CG	This study
grbE-fw	GGC CGA AGC CGA ATG GCT CG	This study
grbE-PacI	GGTTTAATTAA GCG TCG CGT ATG AAG CGT CC	This study
grbE-KpnI	GGTGGTACC TTC GGC ACG ATG GCG GAG CG	This study
grbE-NheI	GGTGCTAGC CGA TGC GAC GAA TCG TGC GC	This study
Bg-UK-fw3	TGG AGC GAC ACC TTC GAG CG	This study
Bg-UK-rv3	CCT TGC ACA TAC TGC GCA CG	This study
grbE-fw2	CGA CAG TAT CGA CGT CCC GG	This study
grbE-rv2	GGG TCG ATG TCG TGC AAC CG	This study
Cml-fw-KpnI	GGTGGTACC CCC GTC AGT AGC TGA ACA GG	[1]
Cml-rv-PacI	GGTTTAATTAA AAC GAC CCT GCC CTG AAC CG	[1]
PlaNRPS-fw	TCG TCA ACG AAC TCG GCG CC	This study
PlaNRPS-PacI	GGTTTAATTAA TGC TGC CCT TCG CAA TGC GC	This study
PlaNRPS-KpnI	GGTGGTACC CAT CTG CAT GAC GCG CTC GC	This study
PlaNRPS-NheI	GGTGCTAGC TGA TCT GCT GGT CGA GGC GG	This study
PlaNRPS-fw3	CGA TCT CGT GCT CGA CGC CC	This study
PlaNRPS-rv3	AAC AGC CCG CCG CAT TGC CG	This study

Table S4: Plasmids used in this study.

Plasmid	Relevant characteristics	Source or reference
pGEM T-easy	TA cloning vector; f1, Amp^R	Promega
pACYC184	General cloning vector; p15A, Tet^R, Cm^R	Invitrogen
pGEM-Δ*grbD*	pGEM T-easy containing *grbD* insertional Cm^R cassette	This study
pGEM-Δ*grbE*	pGEM T-easy containing *grbE* insertional Cm^R cassette	This study
pGEM-Δ*PlaNRPS*	pGEM T-easy containing *plbD* insertional Cm^R cassette	This study
pGL42a_T251A	Modified pGL42a with T251A	[10]
pGL42a_T251A- Δ*PlaNRPS*	pGL42a_T251A containing *plbD* insertional Cm^R cassette	This study

Table S5: Bacterial strains used in this study.

Species	Strain	Relevant characteristics	Source or reference
E. coli	TOP10	General cloning host strain	Invitrogen
	XL1-Blue	General cloning host strain	Stratagene
P. graminis	C4D1M	Wild type; environmental isolate	[11]
	C4D1M Δ*grbD*	Cm^R cassette inserted into *grbD*	This study
	C4D1M Δ*grbE*	Cm^R cassette inserted into *grbE*	This study
B. plantarii	DSM9509	Wild type; environmental isolate	[12]
	DSM9509 Δ*PlaNRPS*	Cm^R cassette inserted into *plbD*	This study
P. caledonica	DSM17062	Wild type; environmental isolate	[13]
B. glumae	DSM9512	Wild type; environmental isolate	[14]
B. gladioli	pv. agaricicola	Wild type; environmental isolate	[15]
	pv. cocovenenans	Wild type; environmental isolate	[16]
	HKI0739	Wild type; environmental isolate	[17]

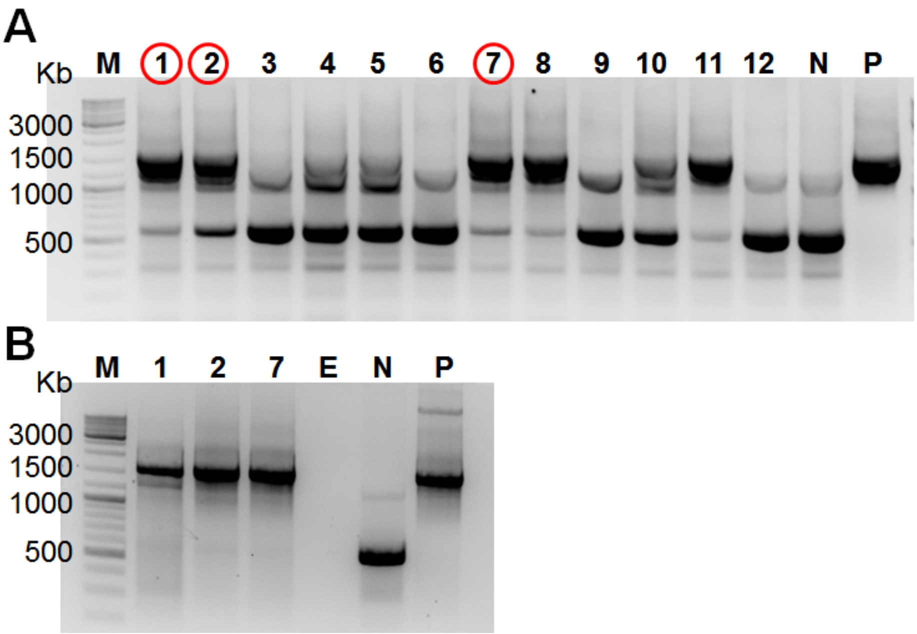

Figure S8. PCR confirmation of knock-out mutants of *B. plantarii*. A. The first colony PCR confirmation of knock-out mutants of *B. plantarii*. Template DNA: *B. plantarii* Δ*plbD* (1−12), wild-type (N), pGL42a_T251A Δ*PlaNRPS* (P). Primer pair PlaNRPS-fw3/PlaNRPS-rv3 and the estimated size of amplicons, wild-type including single crossover mutant; 554 bp and double crossover mutant including single crossover mutant; 1,595 bp. M; marker. Red circle indicates selected mutants for further counter selection on agar plate. B. The final PCR confirmation of selected knock-out mutants of *B. plantarii*, after the second counter selection by 4-chlorophenylalanine, using the same primer pair as that of A. Template DNA: *B. plantarii* Δ*plbD* (1, 2, 7), water (E), wild-type (N), pGL42a_T251A Δ*PlaNRPS* (P).

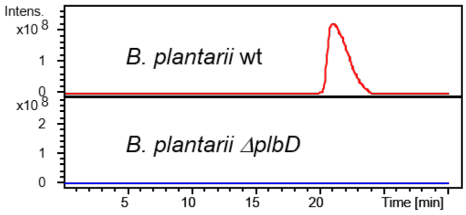

Figure S9. HPLC profiles (EIC 1103.8, negative mode) of culture supernatants obtained from *B. plantarii* wt and knockout strains showing the absence of plantaribactin in the knockout mutant.

Detection of plantaribactin in *B. glumae* cultures

B. glumae DSM9512 cultures were prepared as mentioned before and the obtained supernatants were extracted as described above. EIC traces of crude extracts from *B. plantarii* and *B. glumae* cultures were compared (Figure S10A).

Detection of gladiobactin in *Burkholderia gladioli* strains

To test whether *B. gladioli* strains harboring a homologue of the *plb* gene cluster produce plantaribactin-like siderophores, we cultured *B. gladioli* pv. agaricicola, *B. gladioli* pv. cocovenenans, and *B. gladioli* HKI0739 in MM9 medium (as described above). The obtained crude extract was analyzed by LC-MS. Compared to plantaribactin the retention times of the *B. gladioli* siderophores were marginally shifted (by ~0.3 min), and the observed *m/z* (HRMS) were about 0.03 higher than the *m/z* of plantaribactin (Figure S10B), which indicated slight differences in their structures. Since all investigated *B. gladioli* strains produced the same compound, we named it gladiobactin and focused on one of the extracts for a more detailed analysis. MS/MS experiments on the respective ion detected in the extract of *B. gladioli* pv. agaricicola revealed the presence of a lysine residue in lieu of glutamine present in plantaribactin (Figure S10C). The observed mass of gladiobactin and its iron complex fits to the calculations (HRMS gladiobactin: 1105.5836 (calc. for $C_{45}H_{81}N_{14}O_{18}^{+}$: 1105.5848) Fe-gladiobactin: 1158.4967 (calc. for $C_{45}H_{78}FeN_{14}O_{18}^{+}$: 1158.4962). Bioinformatic analysis of the putatively lysine incorporating modules detected in *B. gladioli* strains shows differences in the Stachelhaus code at 2 positions (Table AS1), and the predicted substrate is in agreement with the results from HR-MS/MS analysis (Figure S10C).

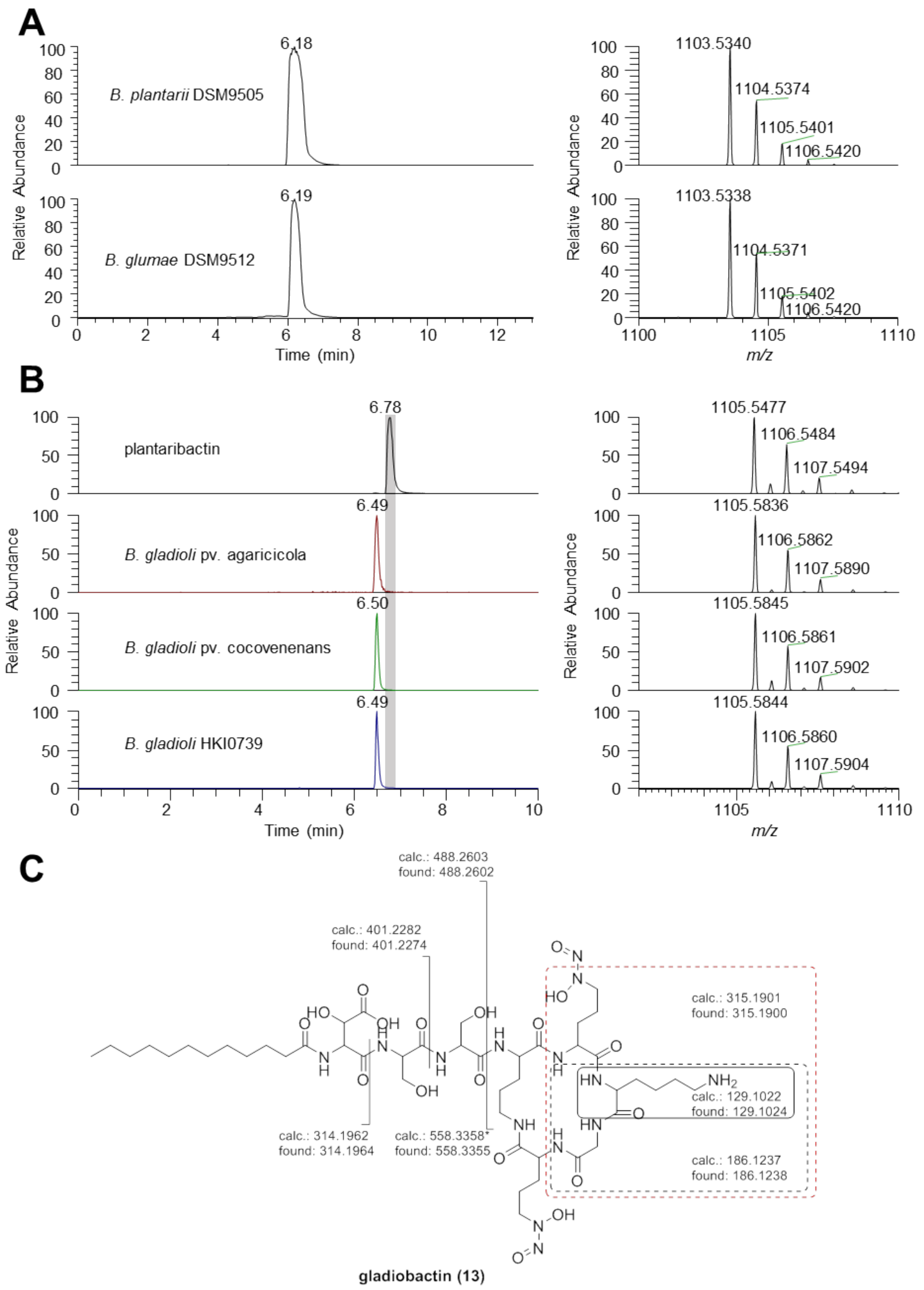

Figure S10. Detection of plantaribactin and gladiobactin in cultures of *B.* glumae and *B.* gladioli. **A)** HPLC profiles (EIC 1105.54, positive mode (measured on a QExactive HF-X) of extracts from culture supernatants obtained from *B. plantarii* wt and *B. glumae*. Isotope patterns indicate that plantaribactin is produced by both strains. **B)** HPLC profiles (EIC 1105.5, positive mode) of extracts from culture supernatants of different *B. gladioli* strains compared to a reference of plantaribactin (measured on a QExactive). Retention time shifts and differences in HRMS indicate the presence of a different compound produced by *B. gladioli* strains. **C)** Structure of gladiobactin as elucidated by given characteristic fragments detected in HR-MS/MS experiments. * indicates fragments with two additional losses of NO.

Corn Cultivation Medium

The solutions listed below were prepared and autoclaved separately. Distilled water was also autoclaved and the sterilized solutions were added.

Table S6: Components of corn cultivation medium.

Component	Composition (final conc.)	For 1 L of medium
**Trace element solution (1000x)**	1.55 mg mL^{-1} $ZnSO_4$ x $7H_2O$ (5.4 µM)<br>1.55 mg mL^{-1} $MnSO_4$ x H_2O (9.18 µM)<br>2.84 mg mL^{-1} H_3BO_3 (46 µM)<br>2.25 mg mL^{-1} $CuSO_4$ x $5H_2O$ (9 µM)<br>0.48 mg mL^{-1} Na_2MoO_4 x $2H_2O$ (2 µM)	1 mL
**Part A**	100.5 g L^{-1} $MgSO_4$ x $7H_2O$ (4.06 mM)<br>13.5 g L^{-1} KH_2PO_4 (1 mM)	10 mL
**Part B**	183 g L^{-1} $Ca(NO_3)_2$ x $4H_2O$ (7.75 mM)<br>53 g L^{-1} KNO_3 (5.25 mM)	10 mL
**H_2O**		979 mL

Rice cultivation medium[18]

The solutions were combined as listed below, pH was adjusted to 5.7 and autoclaved.

Table S7: Components of rice cultivation medium.

Stock solution #	Component	Stock conc.	For 1 L of medium
**1**	NH_4NO_3	1 M	1 mL
**2**	NaH_2PO_4 x 2 H_2O	0.6 M	1 mL
**3**	K_2SO_4	0.3 M	1 mL
**4**	$CaCl_2$ x 2 H_2O	0.2 M	1 mL
**5**	$MgCl_2$ x 6 H_2O	0.4 M	1 mL
**6**	H_3BO_3	500 mM	0.1 mL
	$MnSO_4$ x 5 H_2O	90 mM	
	$CuSO_4$ x 5 H_2O	3 mM	
	$ZnSO_4$ x 7 H_2O	7 mM	
	Na_2MoO_4 x 2 H_2O	1 mM	
**-**	H_2O		994.9 mL

Cultivation of Corn Plants

Commercially available maize seeds (*Zea mays* L. ssp. saccharata; Kiepenkerl) were surface-sterilized in 4.5% *v/v* sodium hypochlorite for 10 min and subsequently rinsed 5 times with 50 mL sterile distilled water. Seeds were germinated in rolled filter papers soaked with saturated $CaSO_4$ solution for 3 days at 30 °C in the dark. Seedlings were grown hydroponically (under a fluorescent lamp 16 h light, 8 h darkness) in falcon tubes containing 45 mL of Corn Cultivation medium for up to 10 days while keeping the roots dark. Roots were then harvested for *in vitro* NO release assays.

Cultivation of Rice Plants

Rice seeds (*Oryza sativa* 'Arborio Bianco', magicgardenseeds) were peeled and surface sterilized in 70% EtOH for 60 seconds and subsequently in 3% NaOCl solution for 15 min. After removing the liquid, seed were washed thoroughly with sterile water. Sterile seeds were then germinated in distilled water for 3 days in the dark. Germinated seeds were transferred to culture tubes filled with glass rings as solid support and 3 mL rice cultivation medium. Tubes were kept at room temperature under fluorescent light (16 h light, 8 h darkness).

***In vitro* NO-Release Assay**

To study the NO release in a corn root extract, the fluorescent probe 2,3-diaminonaphtalene (DAN, dissolved in DMF) was used. For the *in vitro* assay, approximately one week old maize plants were harvested and the roots were rinsed with distilled water, separated from their shoot and frozen in liquid nitrogen. They were then ground with mortar and pestle and the plant material was suspended in 66 mM NaH_2PO_4 buffer (pH 6; 1 mL g^{-1} root). The extract was centrifuged at 4 °C and max. speed for 10 min. 20 µL of the supernatant were used for the *in vitro* assay. Assays were carried out in 1.5 mL Eppendorf tubes with the following final concentrations: 2 mM of the test-compound (4 mM for amino acids test-compounds), 1.2 mM H_2O_2, 0.2 mM DAN. Buffer was added to a final volume of 51.5 µL and the reactions were mixed. After incubation for 20 min in the dark, the reactions were stopped by addition of 250 µL 10 mM NaOH and 280 µL of the solutions were transferred to a black 96-well plate. Fluorescence was measured using a Varioskan LUX microplate reader (Thermo Fisher) ($\lambda_{Ex.}$ = 375 nm, $\lambda_{Em.}$ = 415 nm). Every assay was performed in triplicates and every replicate was measured three times. Each experiment (same root extract; measured in one session) was normalized to the maximal mean of all replicates (set to 100). Bar diagrams in figure 4 show means of these normalized replicates with standard deviation between the normalized replicates.

***In planta* NO Release Assay**

Young corn seedlings (2-3 days old, germinated as above) were placed in a vial containing 4 mL standard corn cultivation medium with 10 µM 4,5-diaminofluoresceine-2 diacetate (DAF-2 DA) and incubated at room temperature for 30 min. Seedlings were then removed, and the root was rinsed with deionized water before placing them in nutrient solution containing 100 µM gramibactin (n=3) or no additive (control, n=2). After 1.5–2 h, roots were placed on styrofoam as a solid support and were cut with a wet razorblade. Upon NO release formed triazolofluorescein was visualized using a Zeiss CLSM 710 confocal laser-scanning microscope (Jena, Germany), and Zen software (Zeiss) has been used to generate the images with λ_{Ex} = 485 nm and λ_{Em} = 538 nm. The exact same parameters have been used for all images.

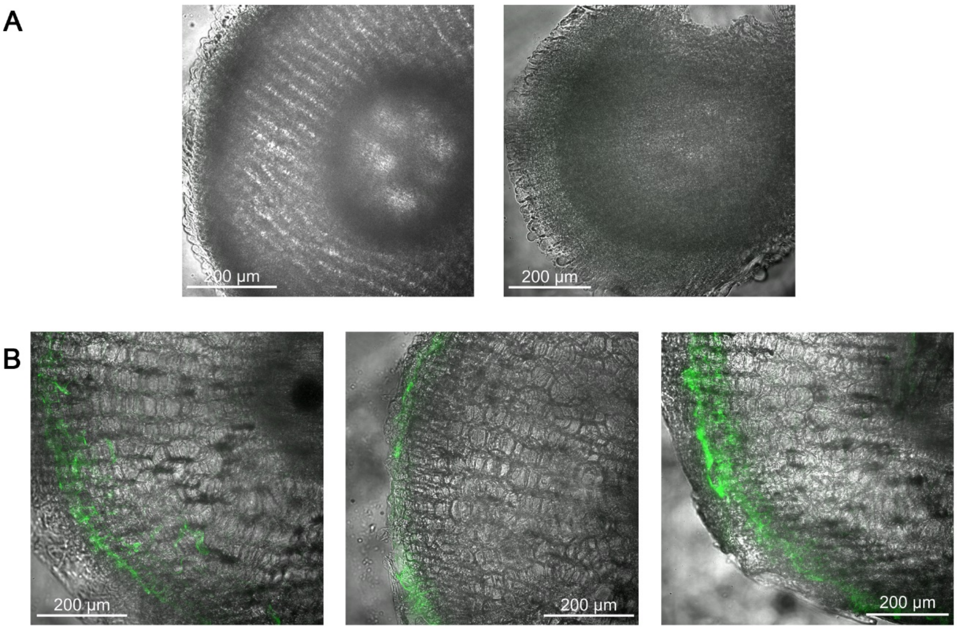

Figure S11. *In planta* nitric oxide imaging of corn root sections stained with DAF-2 DA. Green fluorescence indicates nitric oxide. A) Control roots, treated with corn cultivation medium. B) Roots treated with 100 µM gramibactin in corn cultivation medium.

Rice seedlings (germinated as described above) were cultured in rice culture medium (containing 0.8% acetonitrile and 100 µM plantaribactin; n=3) or rice medium (containing 0.8% acetonitrile, as control, n=3). After 7 days, plants were removed from the medium and roots were washed with 20 mM HEPES (pH 7.5). Staining solution was prepared freshly (10 µM DAF-FM DA in 20 mM HEPES (pH 7.5)) and roots were incubated in the staining solution for 1 h in the dark. Afterwards roots were washed again with HEPES buffer and analysed using a Zeiss Axio Observer 7 Spinning Disk Confocal Microscope (SDCM, ZEISS, Jena, Germany) with λ_{Ex} = 493 nm and λ_{Em} = 517 nm. Z-Stacks were recorded through the root tip and images were processed using Fiji. Contrast was adjusted in all images to same levels and intensities over each Z-stack was summed.

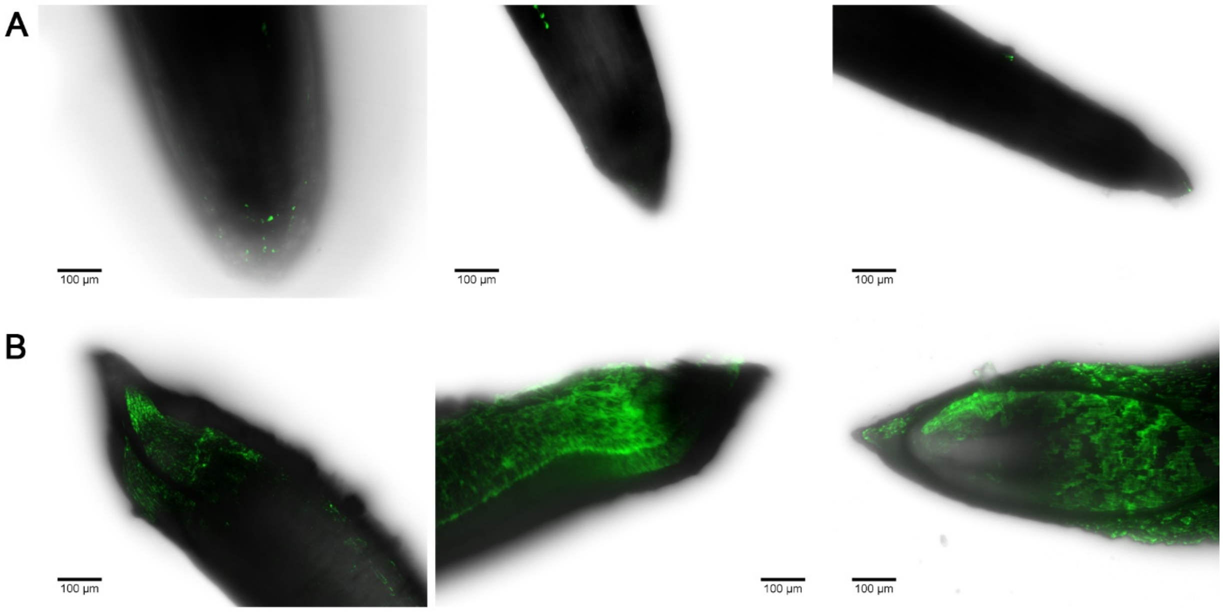

Figure S12. *In planta* nitric oxide imaging of rice roots stained with DAF-FM DA. Green fluorescence indicates nitric oxide. A) Control roots, treated with rice cultivation medium. B) Roots treated with 100 µM plantaribactin in rice cultivation medium.

To prove that the released nitric oxide originates from the used diazeniumdiolate, we incubated five hydroponically grown rice plants in a falcon tube containing 10 mL of fresh rice culture medium and 100 µM plantaribactin. As a control, 10 mL of the same treatment solution were incubated without rice plants. After 22 h, 2 mL of both liquids were loaded on Sep-Pak® cartridges (C_{18}, Waters). After washing the cartridges with 2 mL water, elution was performed with 5 mL methanol. The solvent was evaporated, and the solid residue dissolved in 200 µL methanol prior to analysis using LC-HRMS. EICs for plantaribactin (*m/z* 1105.5477 $[M+H]^+$ and respective compounds resulting from cleavage of nitric oxide (*m/z* 1074.5419 $[M\text{-NO+H}]^+$; *m/z* 1043.5360 $[M\text{-2NO+H}]^+$) were integrated and normalized to the plant-free sample. We found a decrease in plantaribactin levels and an increase of the species with one cleaved nitric oxide. The amount of plantaribactin with 2 molecules of nitric oxide cleaved remained the same. Together with *in vitro* assays shown in figure 4E this demonstrates that the stained NO in rice plant roots indeed originates from plantaribactin.

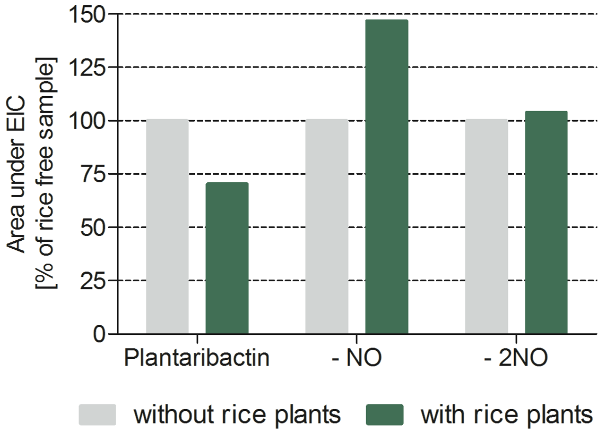

Figure S13. Semiquantification of plantaribactin cleavage products in hydroponic solutions with or without rice plants after 22h of incubation.

Synthetic Procedures

Synthesis of L-graminine (2)

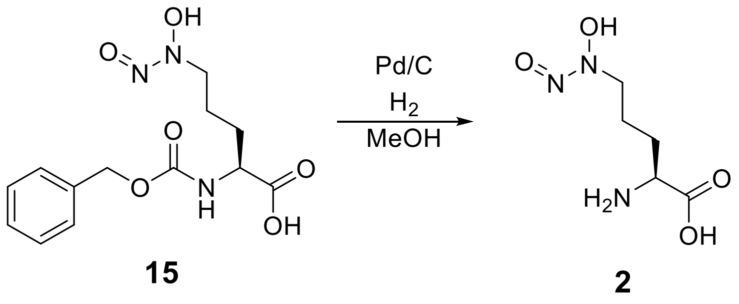

L-graminine (**2**) was synthesized from Cbz-L-graminine (**15**)[1]. The synthesis was carried out analogously to a procedure described by FELPIN *et al.*[19] To a solution of Cbz-L-graminine (**15**, 38.24 mg, 122.84 µmol) in MeOH (8 mL) was added Pd/C (10 wt. % loading; Pd: 1.31 mg, 12.28 µmol, 0.1 eq.). The resulting mixture was stirred under H_2 atmosphere for 20 min. The reaction mixture was filtered and the remaining solid washed with MeOH. Water was used to filter off the desired product. Freeze-drying of the aqueous phase yielded the L-graminine (**2**) (21 mg, 118.54 µmol, 96.5%) as a white fluffy solid.

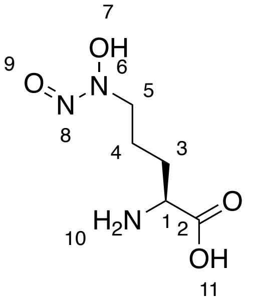

^{1}H-NMR (500 MHz, D_2O/NaOD): δ =3.96 (t, 2H; H-5), 3.19 (t, 1H; H-1), 1.80 (q, 2H; H-4), 1.50 (m, 2H; H-3).

HRMS

m/z ($C_5H_{10}N_3O_4^-$) [M-H]$^-$ = calc.: 176.0677; found: 176.0668

Synthesis of *N*^{2}-Benzyloxycarbonyl-*N*^{5}-formyl-*N*^{5}-hydroxy-l-ornithine (17)

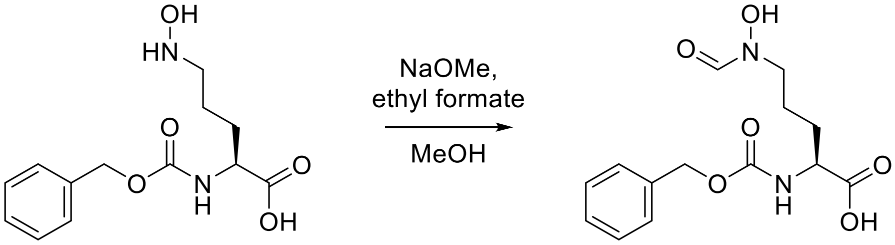

16 **17**

The synthesis was carried out analogously to a procedure described by Gate *et al.*[20]

To the crude hydroxylamine **16** (0.49 g, 1.74 mmol), sodium methoxide in MeOH (5%; 17.4 mL, 8.7 mmol, 5 eq.) and ethyl formate (3.5 mL, 43.5 mmol, 25 eq.) were added. The mixture was stirred under an atmosphere of argon for 24 h at room temperature. Afterwards, the volatiles were removed and the product was dissolved in 0.5 N HCl (20 mL). Extraction with EtOAc (3 x 20 mL) and DCM (3 x 20 mL) and evaporation of the combined organic layers gave crude formylated hydroxylamine (374.5 mg) as a brown oil. The crude product was purified further by preparative HPLC. The resulting yellow oil was freeze-dried to give 257.7 mg of formylated hydroxylamine **17** (0.83 mmol, 47.8%) as a white fluffy solid.

Analytics:

Chemical Formula: $C_{14}H_{18}N_2O_6$

Molecular Weight: 310.3060 g mol^{-1}

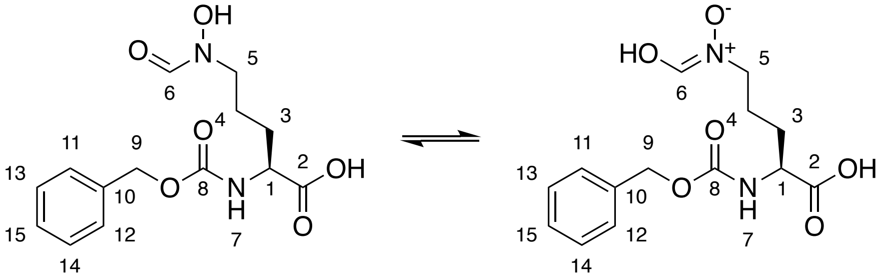

^{1}H-NMR (500 MHz, DMSO- d_6): δ = 8.22 (s, 1/2H, H-6)*, 7.87 (s, 1/2H, H-6)*, 7.52 (d, 1H, H-7), 7.31 (m, 5H, H-11 to H-15), 5.02 (s, 2H, H-9), 3.94 (m, 1H, H-1), 3.39 (m, 2H, H-5), 1.59 (m, 4H, H-3, H-4).

^{13}C-NMR (125 MHz, DMSO-d_6): δ = 173.68 (1C, C-2), 161.69 (1/2C, C-6)*, 157.07 (1/2C, C-6)*, 156.09 (1C, C-8), 136.95 (1C, C-10), 128.29 (2C, C-13, C-14), 127.75 (1C, C-15), 127.65 (2C, C-11, C-12), 65.36 (1C, C-9), 53.50 (1/2C, C-1)*, 49.77 (1/2C, C-1)*, 48.62 (1/2C, C-5)*, 45.23 (1/2C, C-5)*, 27.88 (1/2C, C-3)*, 27.54 (1/2C, C-3)*, 23.51 (1/2C, C-4)*, 22.86 (1/2C, C-4)*.

* signals split because of tautomerism (see above)

LC-HRESIMS

m/z ($C_{14}H_{17}N_2O_6^-$) [M-H]$^-$ = calc.: 309.1092; found: 309.1092

Synthesis of *N*⁵-Formyl-*N*⁵-hydroxy-l-ornithine (14)

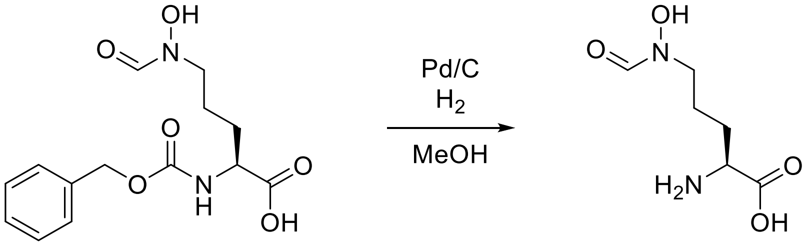

17 **14**

The synthesis was carried out analogously to a procedure described by Lin and Miller.[21]

The formylated hydroxylamine **17** (174.9 mg, 0.56 mmol) was dissolved in dry MeOH (12 mL). Palladium on activated charcoal (5%; 120.0 mg, 56.4 µmol, 0.1 eq.) was added to the solution. Under stirring, H_2 was passed through the reaction mixture for 20 min. Subsequently, Pd/C was filtered off and washed with MeOH and water. The flow-through was filtered with a Whatman® 0.45µ syringe filter and then concentrated and freeze-dried to give formyl graminine (**14**) (95.8 mg, 543.8 µmol, 96.5%) as white fluffy solid.

Analytics:

Chemical Formula: $C_6H_{12}N_2O_4$

Molecular Weight: 176.1720 g mol^{-1}

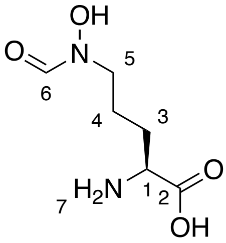

^{1}H-NMR (600 MHz, D_2O/NaOD): δ = 7.13 (s, 1H, H-6), 2.89 (t, 2H, H-5), 2.73 (t, 1H, H-1), 1.06 (m, 4H, H-3, H-4),

^{13}C-NMR (150 MHz, D_2O/NaOD): δ = 183.16 (1C, C-2), 154.10 (1C, C-6), 55.26 (1C, C-1), 52.36 (1C, C-5), 31.31 (1C, C-3), 22.46 (1C, C-4).

LC-HRESIMS

m/z ($C_6H_{11}N_2O_4^-$) [M–H]$^-$ = calc.: 175.0724; found: 175.0712

Characteristic IR signals

3478 cm^{-1} (m, -NH_2), 3387 cm^{-1} (m, O=C-N), 3000 cm^{-1} (m, C–H), 1672 cm^{-1} (s, O=C-N), 1633 cm^{-1} (s, O=C–O$^-$), 1605 cm^{-1} (m, N-H),1496 cm^{-1} (s, N-O), 1355 cm^{-1} (s, O–H).

UV signals

λ_{max} (pH 2): 201 nm

λ_{max} (pH 12): 229 nm

Structure Elucidation

Megapolibactin A

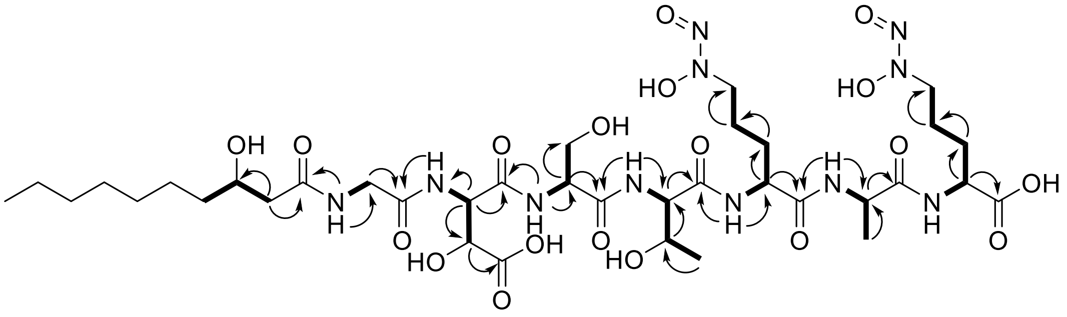

Figure S14. Structure of megapolibactin A as elucidated from 1D- and 2D-NMR experiments. Arrows: HMBC-couplings; bold: ^{1}H-^{1}H-COSY-couplings.

Chemical Formula: $C_{36}H_{63}N_{11}O_{19}$

HRMS

m/z ($C_{36}H_{64}N_{11}O_{19}^{+}$) $[M+H]^{+}$ = calc.: 954.4374; found: 954.4374
m/z ($C_{36}H_{59}FeN_{11}O_{19}^{-}$) $[M–H]^{-}$ = calc.: 1005.3344; found: 1005.3364

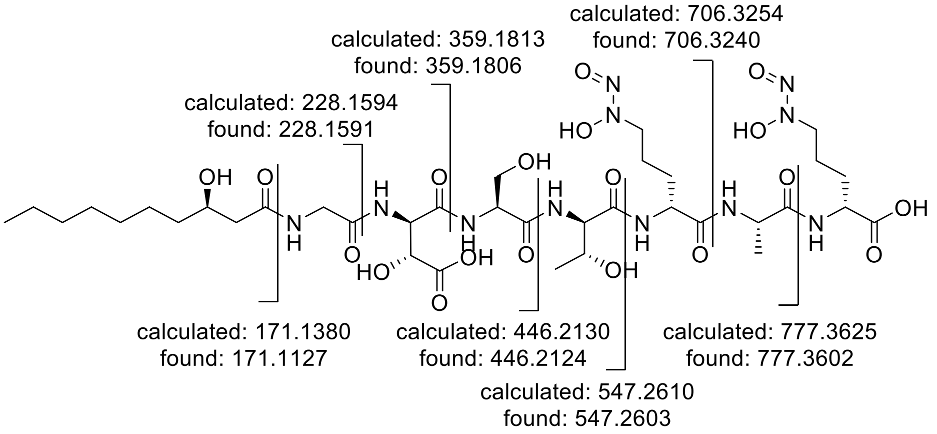

Figure S15. MSMS fragmentation of megapolibactin A. Absolute configuration deduced from comparison with megapolibactin C.

NMR (600 MHz, DMSO-d_6):

Table S8: NMR data of megapolibactin A. 3-HDA: 3-Hydroxydecanoic acid.

Residue	Position	δ_C (m)	δ_H (m, J[Hz])
**Gly**	NH	—	8.36 (d, 5.5)
	CO	169.33 (C)	—
	C_α	42.38 (CH_2)	3.75 (m)
**OH-Asp**	NH	—	8.02 (d, 7.0)
	CO	169.15 (C)	—
	C_α	55.55 (CH)	4.70 (dd, 9.0, 2.6)
	C_β	70.15 (CH)	4.48 (d, 2.5)
	COOH	173.07 (C)	—
**Ser**	NH	—	7.72 (d, 6.5)
	CO	169.51 (C)	—
	C_α	55.62 (CH)	3.75 (m)
	C_β	61.72 (CH_2)	
**Thr**	NH	—	7.83 (d, 8.7)
	CO	170.55 (C)	—
	C_α	58.21 (CH)	4.27 (m)
	C_β	67.80 (CH)	3.75 (m)
	C_γ	20.23 (CH_3)	1.08 (d, 6.1)
**Gra1**	NH	—	8.28 (d, 7.6)
	CO	170.72 (C)	—
	C_α	52.41 (CH)	4.27 (m)
	C_β	28.10 (CH_2)	1.58 (m)
	C_γ	23.03 (CH_2)	1.78 (m)
	C_δ	61.34 (CH_2)	3.75 (m)
**Ala**	NH	—	8.02 (d, 8.9)
	CO	172.04 (C)	—
	C_α	48.25 (CH)	4.27 (m)
	C_β	18.48 (CH_3)	1.17 (d, 7.1)
**Gra2**	NH	—	8.11 (d, 8.2)
	COOH	172.87 (C)	—
	C_α	50.90 (CH)	4.27 (m)
	C_β	27.93 (CH_2)	1.78 (m)
	C_γ	22.83 (CH_2)	1.78 (m)
	C_δ	61.10 (CH_2)	4.07 (m)
**3-HDA**	CO	172.36 (C)	—
	C_α	43.30 (CH_2)	2.24 (d, 6.5)
	C_β	67.46 (CH)	3.75 (m)
	C_γ	36.99 (CH_2)	1.28 (m)
	$C_{\delta-\theta}$	31.27 (CH_2)	1.28 (m)
		29.08 (CH_2)	1.28 (m)
		28.74 (CH_2)	1.28 (m)
		25.04 (CH_2)	1.28 (m)
		22.10 (CH_2)	1.28 (m)
	C_ι	13.98 (CH_3)	0.85 (t, 6.9)

Megapolibactin B

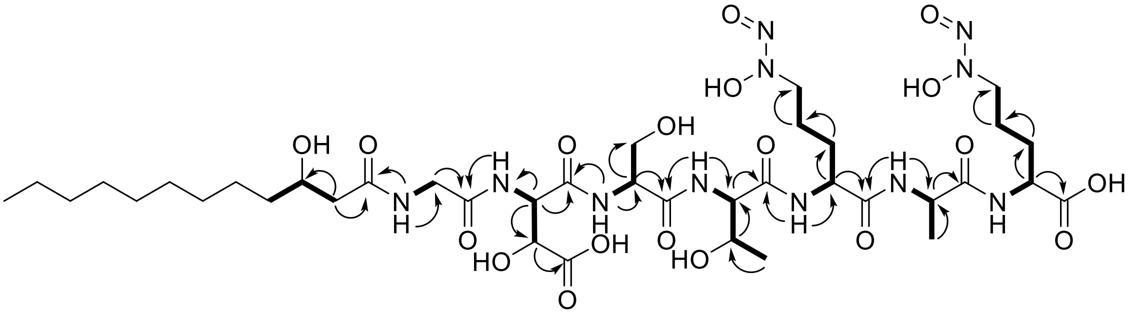

Figure S16. Structure of megapolibactin B as elucidated from 1D- and 2D-NMR experiments. Arrows: HMBC-couplings; bold: ^1H-^1H-COSY-couplings.

Chemical Formula: $C_{38}H_{67}N_{11}O_{19}$

HRMS

m/z ($C_{38}H_{68}N_{11}O_{19}^+$) $[M+H]^+$ = calc.: 982.4687; found: 982.4686

m/z ($C_{38}H_{63}FeN_{11}O_{19}^-$) $[M–H]^-$ = calc.: 1033.3657; found: 1033.3639

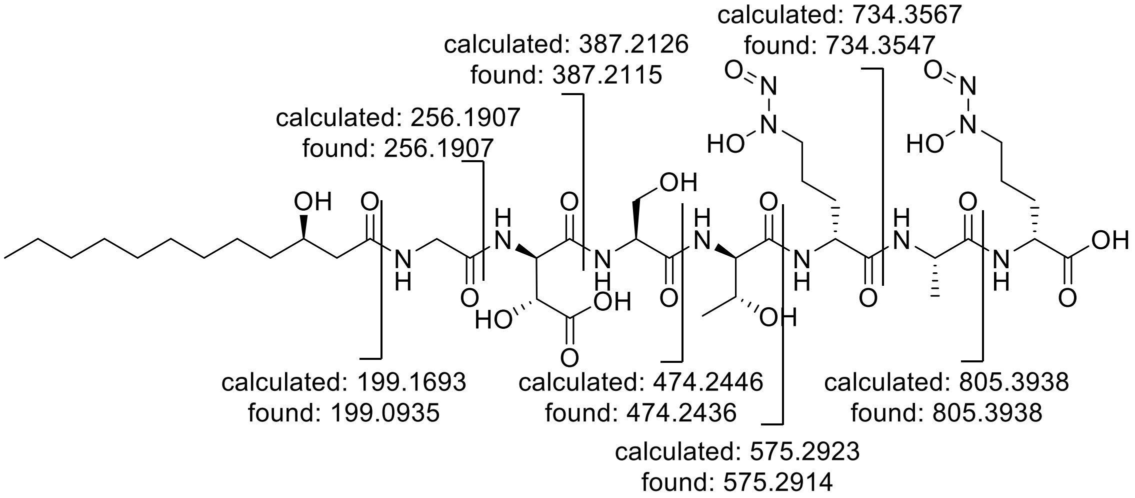

Figure S17. MSMS fragmentation of megapolibactin B. Absolute configurations suggested based on comparison with megapolibactin C.

NMR (600 MHz, DMSO-d_6):

Table S9: NMR data of megapolibactin B. 3-HDoA: 3-Hydroxydodecanoic acid.

Residue	Position	δ_C (m)	δ_H (m. J[Hz])
**Gly**	NH	—	8.35 (d, 5.6)
	CO	169.45 (C)	—
	C_α	42.48 (CH_2)	3.69 (m)
**OH-Asp**	NH	—	8.01 (d, 5.9)
	CO	169.26 (C)	—
	C_α	55.65 (CH)	4.70 (dd, 9.5, 2.3)
	C_β	70.23 (CH)	4.49 (d, 2.3)
	COOH	173.16 (C)	—
**Ser**	NH	—	7.71 (d, 7.9)
	CO	169.63 (C)	—
	C_α	55.72 (CH_2)	4.25 (m)
	C_β	61.79 (CH_2)	3,69 (m)
**Thr**	NH	—	7.81 (d, 9.1)
	CO	170.67 (C)	—
	C_α	58.32 (CH)	4.25 (m)
	C_β	67.87 (CH)	3.69 (m)
	C_γ	20.31 (CH_3)	1.07 (d, 6.2)
**Gra1**	NH	—	8.27 (d, 8.0)
	CO	170.84 (C)	—
	C_α	52.51 (CH)	4.25 (m)
	C_β	28.16 (CH_2)	1.57 (m)
	C_γ	23.12 (CH_2)	1.76 (m)
	C_δ	61.44 (CH_2)	3.69 (m)
**Ala**	NH	—	8.01 (d, 9.1)
	CO	172.17 (C)	—
	C_α	48.37 (CH)	4.25 (m)
	C_β	18.53 (CH_3)	1.18 (d, 7.3)
**Gra2**	NH	—	8.10 (d, 8.5)
	COOH	172.96 (C)	—
	C_α	51.00 (CH)	4.25 (m)
	C_β	28.00 (CH_2)	1.76 (m)
	C_γ	22.91 (CH_2)	1.76 (m)
	C_δ	61.19 (CH_2)	4.05 (m)
**3-HDoA**	CO	172.50 (C)	—
	C_α	43.38 (CH_2)	2.22 (d, 6.7)
	C_β	67.56 (CH)	3.69 (m)
	C_γ	37.07 (CH_2)	1.30 (m)
	$C_{\delta-\kappa}$	31.39 (CH_2)	1.30 (m)
		29.20 (CH_2)	1.30 (m)
		29.18 (CH_2)	1.30 (m)
		29.08 (CH_2)	1.30 (m)
		28.80 (CH_2)	1.30 (m)
		25.13 (CH_2)	1.30 (m)
		22.19 (CH_2)	1.30 (m)
	C_λ	14.06 (CH_3)	0.85 (t, 7.1)

Megapolibactin C

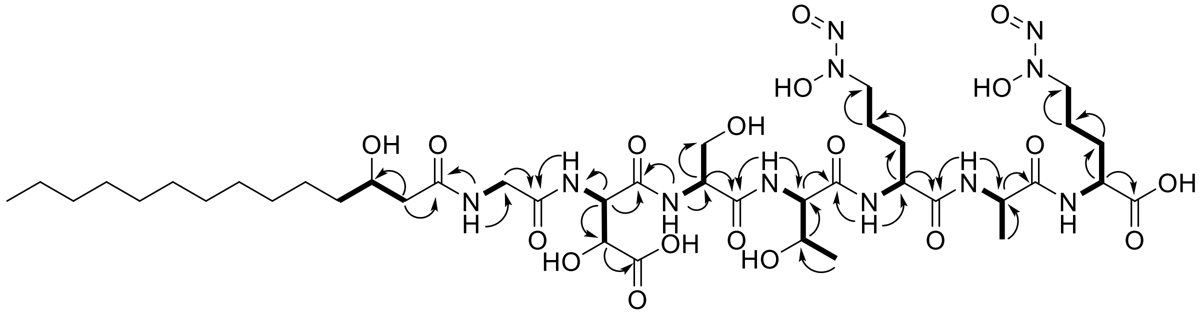

Figure S18. Structure of megapolibactin C as elucidated from 1D- and 2D-NMR experiments. Arrows: HMBC-couplings; bold: ^{1}H-^{1}H-COSY-couplings.

Chemical Formula: $C_{40}H_{71}N_{11}O_{19}$

HRMS

m/z ($C_{40}H_{72}N_{11}O_{19}^{+}$) $[M+H]^{+}$ = calc.:1010.5000 ; found: 1010.5002
m/z ($C_{40}H_{67}FeN_{11}O_{19}^{-}$) $[M–H]^{-}$ = calc.: 1061.3970; found: 1061.3971

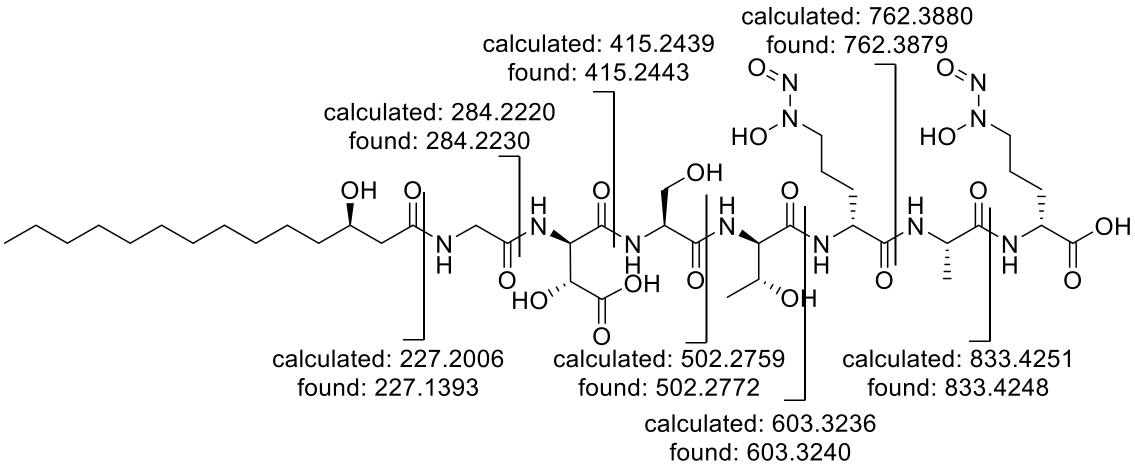

Figure S19. MSMS fragmentation of megapolibactin C. Absolute configurations determined as described above.

NMR (600 MHz, DMSO-d_6):

Table S10: NMR data of megapolibactin C. 3-HtDA: 3-Hydroxytetradecanoic acid.

Residue	Position	δ_C (m)	δ_H (m, J[Hz])
**Gly**	NH	—	8.33 (d, 5.4)
	CO	169.32 (C)	—
	C_α	42.37 (CH_2)	3.73 (m)
**OH-Asp**	NH	—	8.02 (m)
	CO	169.13 (C)	—
	C_α	55.53 (CH)	4.68 (dd, 9.0, 2.3)
	C_β	70.15 (CH)	4.48 (d, 2.6)
	COOH	173.07 (C)	—
**Ser**	NH	—	7.69 (d, 7.5)
	CO	169.49 (C)	—
	C_α	55.62 (CH)	4.25 (m)
	C_β	61.73 (CH_2)	3.73 (m)
**Thr**	NH	—	7.81 (d, 9.1)
	CO	170.54 (C)	—
	C_α	58.20 (CH)	4.25 (m)
	C_β	67.81 (CH)	3.73 (m)
	C_γ	20.23 (CH_3)	1.07 (d, 6.2)
**Gra1**	NH	—	8.26 (d, 7.7)
	CO	170.72 (C)	—
	C_α	52.41 (CH)	4.25 (m)
	C_β	28.11 (CH_2)	1.56 (m)
	C_γ	23.04 (CH_2)	1.77 (m)
	C_δ	61.34 (CH_2)	3.73 (m)
**Ala**	NH	—	7.98 (m)
	CO	172.02 (C)	—
	C_α	48.23 (CH)	4.25 (m)
	C_β	18.50 (CH_3)	1.17 (d, 7.1)
**Gra2**	NH	—	8.09 (d, 8.2)
	COOH	172.88 (C)	—
	C_α	50.89 (CH)	4.25 (m)
	C_β	27.94 (CH_2)	1.77 (m)
	C_γ	22.83 (CH_2)	1.77 (m)
	C_δ	61.09 (CH_2)	4.04 (m)
**3-HtDA**	CO	172.33 (C)	—
	C_α	43.30 (CH_2)	2.21 (d, 6.5)
	C_β	67.45 (CH)	3.73 (m)
	C_γ	37.02 (CH_2)	1.29 (m)
	$C_{\delta-\mu}$	39.14 (CH_2)	1.29 (m)
		31.30 (CH_2)	1.29 (m)
		29.12 (CH_2)	1.29 (m)
		29.08 (CH_2)	1.29 (m)
		29.06 (CH_2)	1.29 (m)
		29.04 (CH_2)	1.29 (m)
		28.72 (CH_2)	1.29 (m)
		25.06 (CH_2)	1.29 (m)
		22.10 (CH_2)	1.29 (m)
	C_ν	13.97 (CH_3)	0.84 (t, 7.0)

Megapolibactin D

Chemical Formula: $C_{36}H_{63}N_{11}O_{20}$

HRMS

m/z ($C_{36}H_{64}N_{11}O_{20}^{+}$) $[M+H]^{+}$ = calc.: 970.4324 ; found: 970.4323

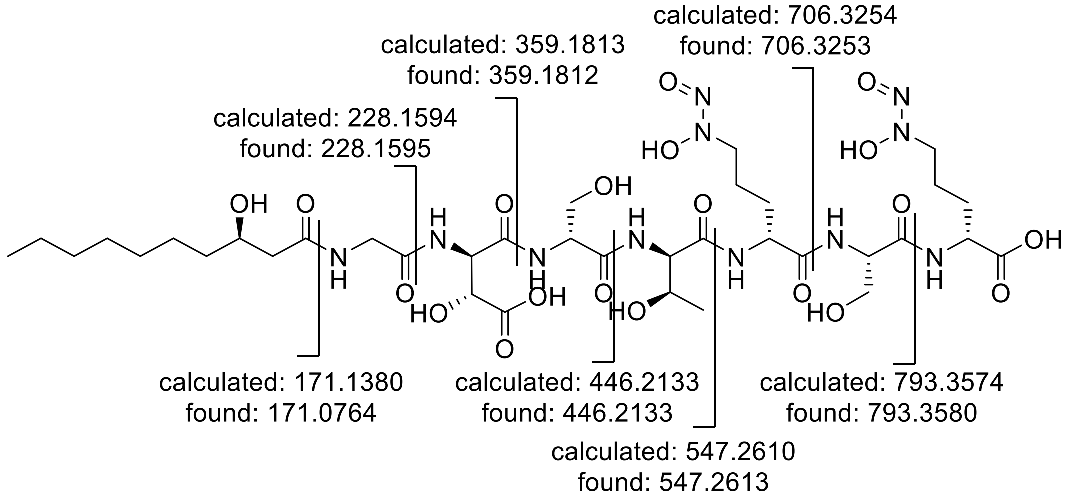

Figure S20. Structure of megapolibactin D as deduced from MSMS experiments. Absolute configurations suggested as in megapolibactin F.

Megapolibactin E

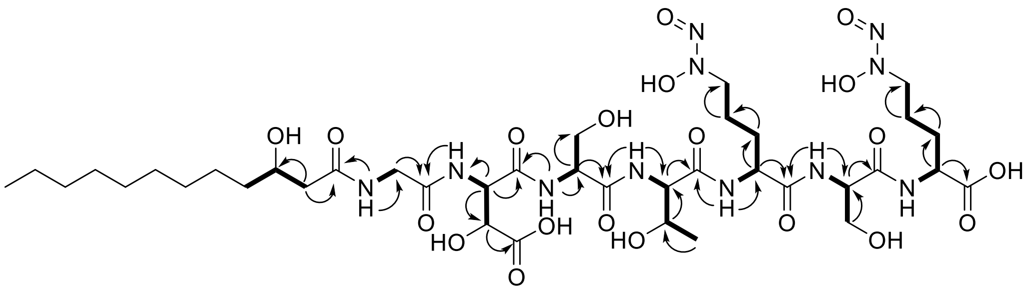

Figure S21. Structure of megapolibactin E as elucidated from 1D- and 2D-NMR experiments. Arrows: HMBC-couplings; bold: ^{1}H-^{1}H-COSY-couplings.

Chemical Formula: $C_{38}H_{67}N_{11}O_{20}$

HRMS

m/z ($C_{38}H_{68}N_{11}O_{20}^{+}$) $[M+H]^{+}$ = calc.: 998.4637; found: 998.4633

m/z ($C_{38}H_{63}FeN_{11}O_{20}^{-}$) $[M–H]^{-}$ = calc.: 1049.3606; found: 1049.3615

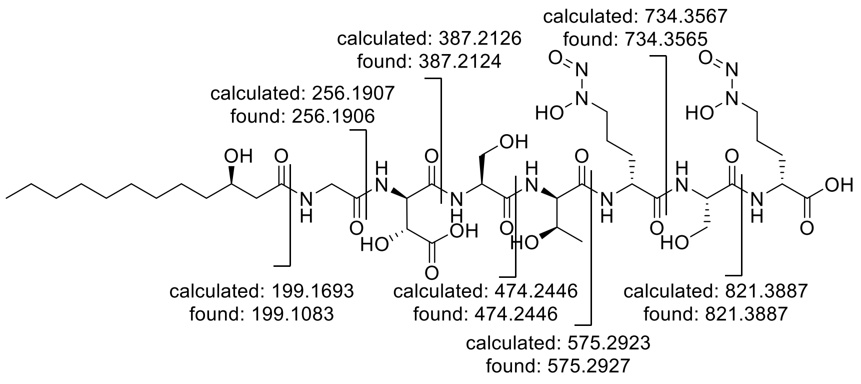

Figure S22. MSMS fragmentation of megapolibactin E. Absolute configuration deduced from comparison with megapolibactin F.

NMR (600 MHz, DMSO-d_6):

Table S11: NMR data of megapolibactin E. 3-HDoA: 3-Hydroxydodecanoic acid.

Residue	Position	δ_C (m)	δ_H (m, J[Hz])
**Gly**	NH	—	8.32 (d, 5.5)
	CO	169.35 (C)	—
	C_α	42.39 (CH_2)	3.73 (m)
**OH-Asp**	NH	—	7.99 (m)
	CO	169.17 (C)	—
	C_α	55.54 (CH)	4.68 (dd, 9.1, 2.4)
	C_β	70.16 (CH)	4.48 (d, 2.1)
	COOH	173.08 (C)	—
**Ser1**	NH	—	7.69 (d, 7.5)
	CO	169.56 (C)	—
	C_α	55.63 (CH_2)	4.20 (m)
	C_β	61.74 (CH_2)	3,73 (m)
**Thr**	NH	—	7.81 (d, 8.7)
	CO	170.57 (C)	—
	C_α	58.18 (CH)	4.20 (m)
	C_β	67.70 (CH)	3.73 (m)
	C_γ	20.05 (CH_3)	1.05 (d, 6.1)
**Gra1**	NH	—	8.26 (d, 7.3)
	CO	171.22 (C)	—
	C_α	52.58 (CH)	4.20 (m)
	C_β	28.28 (CH_2)	1.57 (m)
	C_γ	23.01 (CH_2)	1.76 (m)
	C_δ	61.41 (CH_2)	3.73 (m)
**Ser2**	NH	—	7.99 (m)
	CO	169.68 (C)	—
	C_α	54.84 (CH)	4.20 (m)
	C_β	61.82 (CH_3)	3.50 (m)
**Gra2**	NH	—	8.13 (d, 8.0)
	COOH	172.91 (C)	—
	C_α	51.09 (CH)	4.29 (m)
	C_β	27.88 (CH_2)	1.76 (m)
	C_γ	22.76 (CH_2)	1.76 (m)
	C_δ	61.13 (CH_2)	4.04 (m)
**3-HDoA**	CO	172.36 (C)	—
	C_α	43.33 (CH_2)	2.21 (d, 6.7)
	C_β	67.48 (CH_2)	3.73 (m)
	C_γ	37.02 (CH_2)	1.27 (m)
	$C_{\delta-\kappa}$	31.33 (CH_2)	1.27 (m)
		29.15 (CH_2)	1.27 (m)
		29.13 (CH_2)	1.27 (m)
		29.03 (CH_2)	1.27 (m)
		28.75 (CH_2)	1.27 (m)
		25.08 (CH_2)	1.27 (m)
		22.13 (CH_2)	1.27 (m)
	C_λ	14.00 (CH_3)	0.84 (t, 7.1)

Megapolibactin F

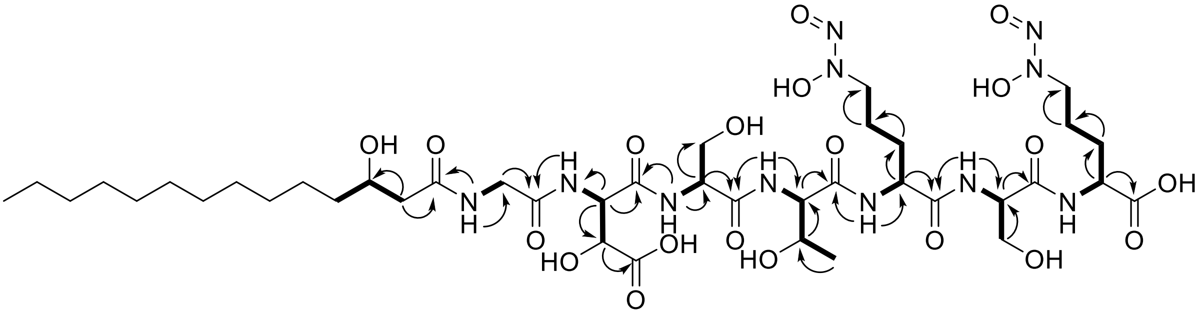

Figure S23. Structure of megapolibactin F as elucidated from 1D- and 2D-NMR experiments. Arrows: HMBC-couplings; bold: ^{1}H-^{1}H-COSY-couplings.

Chemical Formula: $C_{40}H_{72}N_{11}O_{20}$

HRMS

m/z ($C_{40}H_{73}N_{11}O_{20}^{+}$) $[M+H]^{+}$ = calc.: 1026.4950; found: 1026.4946
m/z ($C_{40}H_{67}FeN_{11}O_{20}^{-}$) $[M–H]^{-}$ = calc.: 1077.3919; found: 1077.3922

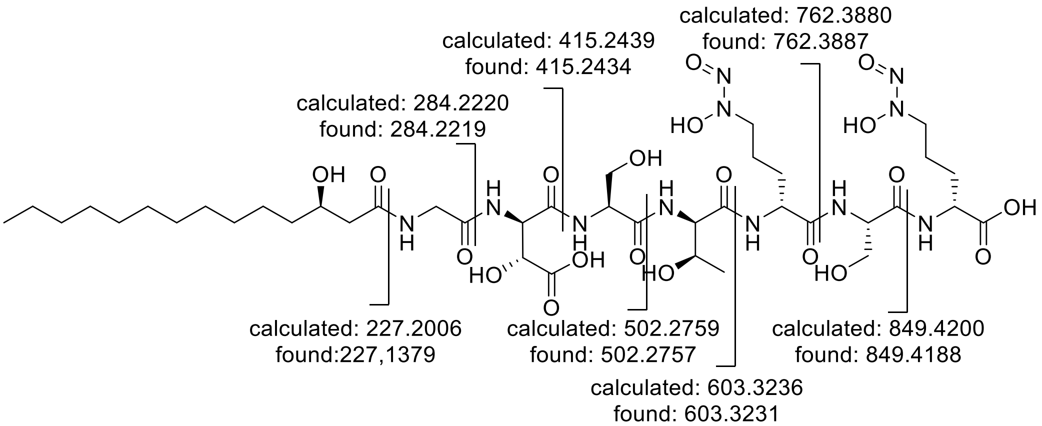

Figure S24. MSMS fragmentation of megapolibactin F. Absolute configuration determined as described above.

NMR (600 MHz, DMSO-d_6):

Table S12: NMR data of megapolibactin F. 3-HtDA: 3-Hydroxytetradecanoic acid.

Residue	Position	δ_C (m)	δ_H (m, J[Hz])
**Gly**	NH	—	8.32 (d, 5.5)
	CO	169.31 (C)	—
	C_α	42.36 (CH_2)	3.72 (m)
**OH-Asp**	NH	—	8.00 (m)
	CO	169.13 (C)	—
	C_α	55.51 (CH)	4.68 (dd, 9.0, 2.5)
	C_β	70.14 (CH)	4.49 (d, 2.5)
	COOH	173.06 (C)	—
**Ser1**	NH	—	7.69 (d, 7.6)
	CO	169.52 (C)	—
	C_α	55.60 (CH_2)	4.29 (m)
	C_β	61.73 (CH_2)	3,72 (m)
**Thr**	NH	—	7.81 (d, 8.7)
	CO	169.65 (C)	—
	C_α	58.15 (CH)	4.29 (m)
	C_β	67.69 (CH)	3.72 (m)
	C_γ	20.03 (CH_3)	1.05 (d, 6.2)
**Gra1**	NH	—	8.26 (d, 7.4)
	CO	170.53 (C)	—
	C_α	52.56 (CH)	4.29 (m)
	C_β	28.27 (CH_2)	1.57 (m)
	C_γ	22.99 (CH_2)	1.77 (m)
	C_δ	61.39 (CH_2)	3.72 (m)
**Ser2**	NH	—	7.99 (m)
	CO	171.19 (C)	—
	C_α	54.81 (CH)	4.29 (m)
	C_β	61.81 (CH_3)	3.50 (m)
**Gra2**	NH	—	8.13 (d, 8.0)
	COOH	172.89 (C)	—
	C_α	51.06 (CH)	4.29 (m)
	C_β	27.86 (CH_2)	1.77 (m)
	C_γ	22.74 (CH_2)	1.77 (m)
	C_δ	61.11 (CH_2)	4.04 (m)
**3-HtDA**	CO	172.31 (C)	—
	C_α	43.31 (CH_2)	2.21 (d, 6.5)
	C_β	67.45 (CH_2)	3.73 (m)
	C_γ	37.01 (CH_2)	1.27 (m)
	$C_{\delta-\mu}$	31.30 (CH_2)	1.27 (m)
		29.11 (CH_2)	1.27 (m)
		29.08 (CH_2)	1.27 (m)
		29.06 (CH_2)	1.27 (m)
		29.04 (CH_2)	1.27 (m)
		28.72 (CH_2)	1.27 (m)
		28.27 (CH_2)	1.27 (m)
		25.07 (CH_2)	1.27 (m)
		22.10 (CH_2)	1.27 (m)
	C_ν	13.97 (CH_3)	0.84 (t, 7.1)

Megapolibactin A_{Cyc}

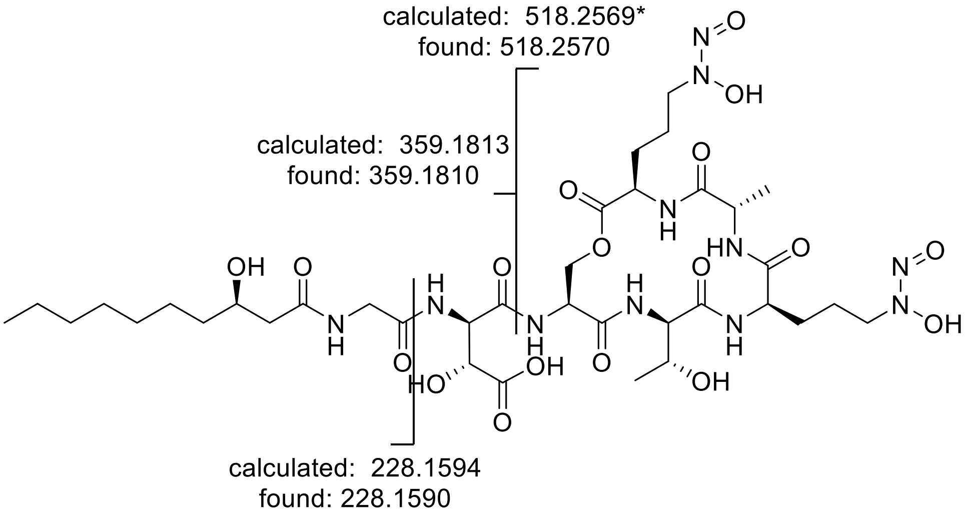

Figure S25. Structure of megapolibactin A_{Cyc} as deduced from MSMS experiments. Absolute configurations are based on comparison with megapolibactin C. * indicates fragments with additional loss of 2 NO.

Chemical Formula: $C_{36}H_{61}N_{11}O_{18}$

HRMS

m/z ($C_{36}H_{62}N_{11}O_{18}^{+}$) $[M+H]^{+}$ = calc.: 936.4269; found: 936.4262
m/z ($C_{36}H_{59}FeN_{11}O_{18}^{+}$) $[M+H]^{+}$ = calc.: 989.3383; found: 989.3383

Megapolibactin B_{Cyc}

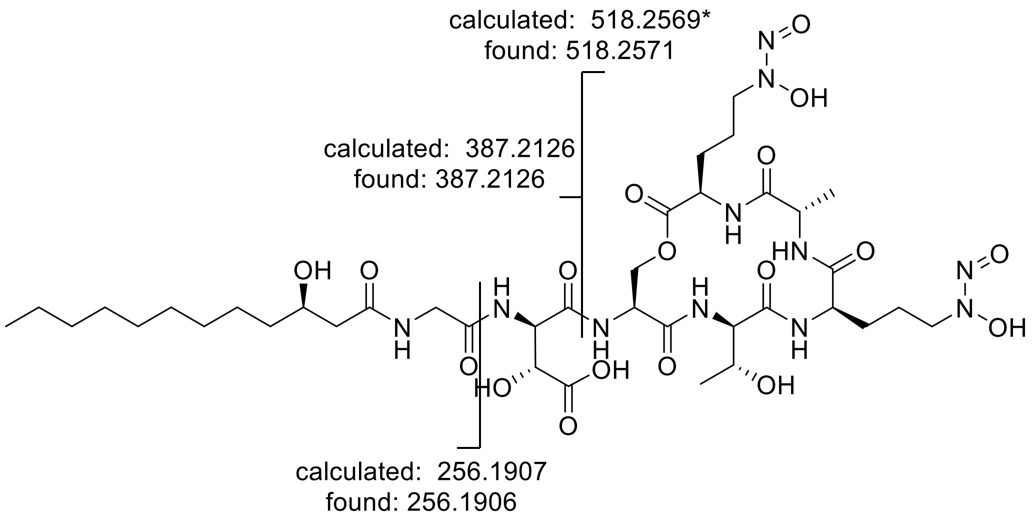

Figure S26. Structure of megapolibactin B_{Cyc} as deduced from MSMS experiments. Absolute configurations are based on comparison with megapolibactin C. * indicates fragments with additional loss of 2 NO.

Chemical Formula: $C_{38}H_{65}N_{11}O_{18}$

HRMS

m/z ($C_{38}H_{66}N_{11}O_{18}^{+}$) $[M+H]^{+}$ = calc.: 964.4582; found: 964.4574
m/z ($C_{38}H_{63}FeN_{11}O_{18}^{+}$) $[M+H]^{+}$ = calc.: 1017.3696; found: 1017.3697

Plantaribactin

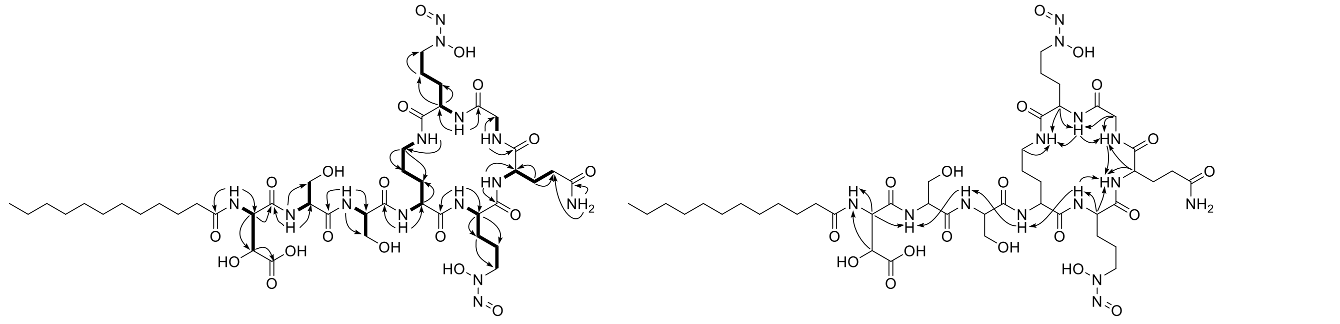

Figure S27. Key COSY and HMBC correlations (left) that led to the identification of respective amino acid components. Key NOESY correlations (right) led to the connection of the amino acid partial structures and to the final amino acid sequence.

Chemical Formula: $C_{44}H_{76}N_{14}O_{19}$

HRMS

m/z ($C_{44}H_{77}N_{14}O_{19}^{+}$) $[M+H]^{+}$ = calc.: 1105.5484; found: 1105.5483
m/z ($C_{44}H_{74}FeN_{14}O_{19}^{+}$) $[M+H]^{+}$ = calc.: 1158.4599; found: 1158.4608
m/z ($C_{44}H_{74}GaN_{14}O_{19}^{+}$) $[M+H]^{+}$ = calc.: 1171.4505; found: 1171.4505

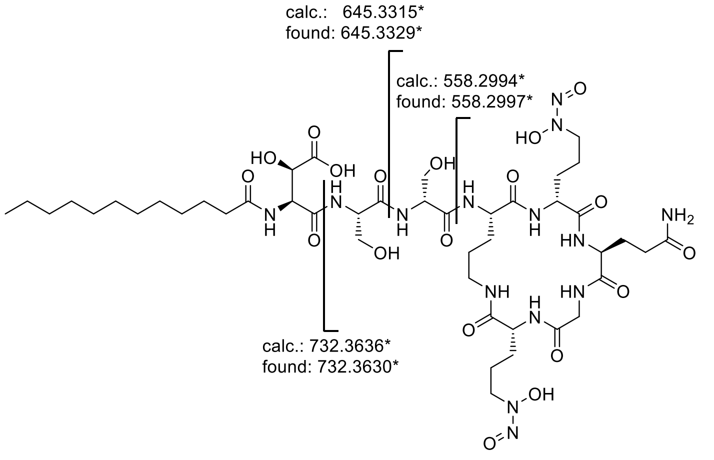

Figure S28. Detected MSMS fragments. * with 2 losses of NO

NMR (600 MHz, DMSO-d_6/THF-d_8 (1:3; *v*:*v*):

Table S13: NMR data of plantaribactin.

Residue	Position	δ_C	δ_H
**HO-Asp**	NH	-	8.10
	CO	169.5	-
	C_α	55.5	4.85
	C_β	71.7	4.16
	COOH	173.4	-
**Ser1**	NH	-	8.1
	CO	170.5	-
	C_α	56.4	4.26
	C_β	61.7	3.80
**Ser2**	NH	-	7.91
	CO	170.5	-
	C_α	56.3	4.21
	C_β	61.7	3.72
**Orn**	NH	-	7.78
	CO	172.1	-
	C_α	53.9	4.22
	C_β	28.5	1.61
	C_γ	25.5	1.55
	C_δ	37.8	3.39 & 2.99
**Gra1**	NH	-	7.56
	CO	172.2	-
	C_α	52.4	4.39
	C_β	27.9	1.73
	C_γ	22.9	1.89
	C_δ	61.2	4.14
**Gln**	NH	-	8.60
	CO	172.0	-
	C_α	53.6	4.19
	C_β	27.0	2.09 & 1.89
	C_γ	31.4	2,21
	**C**ON**H**$_2$	173.9	7.20 & 6.65
**Gly**	NH	-	8.20
	CO	169.1	-
	C_α	42.9	3.77
**Gra2**	NH	-	7.88
	CO	171.9	-
	C_α	52.7	4.26
	C_β	27.9	1.85
	C_γ	23.5	1.97
	C_δ	61.2	4.14

Analysis of putative biosynthetic gene clusters

P. caledonica

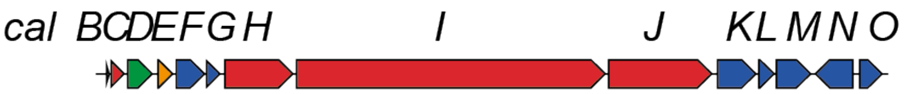

Figure S29. Putative biosynthetic gene cluster found in the genome of *P. caledonica*.

Table S14. Annotation of deduced proteins from *cal* gene cluster.

Protein	Size (aa)	Putative Function	Sequence (identity/similarity)	Query coverage	Accession No.
CalB	77	MbtH-like protein	MbtH-like protein (66%/80%) *Pseudomonas aeruginosa*	84%	2GPF_A
CalC	269	Thioesterase	Thioesterase (32%/44%) *Streptomyces coelicolor*	89%	3QMV_A
CalD	478	Oxidoreductase	SznF (27%/41%) *Streptomyces achromogenes ssp. streptozoticus*	85%	6M9S_A
CalE	300	unknown function	-		
CalF	548	Transporter	Pcat1 (26%/42%) *Hungateiclostridium thermocellum*	81%	4RY2_A
CalG	272	Transporter	Hmuv (35%/53%) *Yersinia pestis*	91%	4G1U_C
CalH	1273	NRPS (FAAL-T-C)	-		
CalI	5639	NRPS (A-T-C-A-T-C-A-T-C-A-T-E-C-A-T)	-		
CalJ	1911	NRPS (C-A-T-E)	-		
CalK	730	TonB dependent Transporter	FetA (40%/56%) *Pseudomonas fluorescens*	90%	3QLB_A
CalL	315	ferric siderophore binding protein	FhuD (34%/49%) *Escherichia coli*	82%	1ESZ_A
CalM	663	Transporter	Hmuv (35%/51%) *Yersinia pestis*	82%	4G1U_A
CalN	714	ferric hydroxamate receptor	FhuA (26%/40%) *Escherichia coli*	90%	1QJQ_A
CalO	426	unknown function	-		

P. megapolitana

In the publicly available genome sequence of *P. megapolitana* DSM23488 (NCBI Accession number NZ_FOQU00000000) misses parts of *megI* and *megJ* in the gene cluster. They are however located on a distinct contig and contain the genetic information for 2 NRPS modules. PCR experiments led to the assumption, that this contig was not correctly assembled and is actually part of the *meg* cluster. We therefore decided to resequence the genome of this strain. The following data originates from this sequencing approach and the predicted NRPS architecture is in accordance with the identified megapolibactins in term of collinearity.

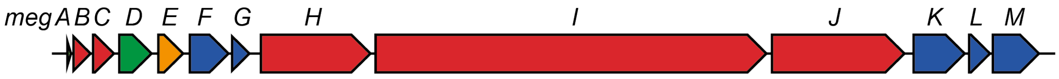

Figure S30. Putative biosynthetic gene cluster found in the genome of *P. megapolitana*.

Table S15. Annotation of deduced proteins from *meg* gene cluster.

Protein	Size (aa)	Putative Function	Sequence (identity/similarity)	Query coverage	Accession No.
MegA	81	MbtH-like protein	MbtH-like protein (72%/83%) *Pseudomonas aeruginosa*	74%	2PST_X
MegB	270	Thioesterase Type II	RifR (35%/51%) *Amycolatopsis mediterranei*	86%	3FLB_A
MegC	322	Dioxygenase	(31%/45%) *Arabidopsis thaliana*	83%	1Y0Z_A
MegD	478	Oxidoreductase	SznF (26%/40%) *Streptomyces achromogenes ssp. streptozoticus*	85%	6M9S_A
MegE	258	unknown function	-		
MegF	565	Transporter	Pcat1 (26%/44%) *Hungateiclostridium thermocellum*	85%	4RY2_A
MegG	279	Transporter	Hmuv (35%/53%) *Yersinia pestis*	91%	4G1U_C
MegH	1533	NRPS (C-A-T-C)	-		
MegI	5362	NRPS (A-T-C-A-T-C-A-T-C-A-T-E-C-A-T)	-		
MegJ	1843	NRPS (C-A-T-E-TE)	-		
MegK	738	TonB dependent Transporter	FetA (40%/56%) *Pseudomonas fluorescens*	93%	3QLB_A
MegL	324	ferric siderophore binding protein	FhuD (31%/49%) *Escherichia coli*	80%	1ESZ_A
MegM	668	Transporter	Hmuv (36%/51%) *Yersinia pestis*	83%	4G1U_A

B. plantarii

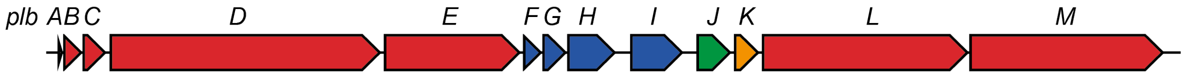

Figure S31. Putative biosynthetic gene cluster found in the genome of *B. plantarii*.

Table S16. Annotation of deduced proteins from *plb* gene cluster.

Protein	Size (aa)	Putative Function	Sequence (identity/similarity)	Query coverage	Accession No.
PlbA	75	MbtH-like protein	MbtH-like protein (68%/86%) *Pseudomonas aeruginosa*	80%	2PST_X
PlbB	268	Thioesterase Type II	RifR (36%/51%) *Amycolatopsis mediterranei*	94%	3FLB_A
PlbC	330	Dioxygenase	(28%/42%) *Arabidopsis thaliana*	92%	1Y0Z_A
PlbD	3717	NRPS (C-A-T-E-C-A-T-C-A-T)	-		
PlbE	1876	NRPS (C-A-T-E-TE)	-		
PlbF	264	Transporter	Hmuv (34%/54%) *Yersinia pestis*	92%	4G1U_C
PlbG	339	ferric siderophore binding protein	FhuD (32%/47%) *Escherichia coli*	79%	1ESZ_A
PlbH	670	Transporter	Hmuu (34%/51%) *Yersinia pestis*	85%	4G1U_A
PlbI	731	TonB dependent Transporter	FetA (26%/40%) *Pseudomonas fluorescens*	93%	3QLB_A
PlbJ	478	Oxidoreductase	SznF (26%/39%) *Streptomyces achromogenes ssp. streptozoticus*	85%	6M9S_A
PlbK	352	Unknown function	-		
PblL	2834	NRPS (FAAL-T-C-A-T-C-A-T)	-		
PblM	2667	NRPS (C-A-T-E-C-A-T)	-		

Prediction of A-domain substrate specificity

Table S17. Predicted A-domain substrate specificity together with incorporated amino acids from the *cal* NRPS in the *P. caledonica* genome. Gra: graminine.

	Stachelhaus Code								predicted amino acid		found
									NRPS analysis[22]	antiSMASH	
**A1**	D	L	T	K	V	G	H	V	Asp	Asp	3-OH-Asp
**A2**	D	F	W	N	I	G	M	V	Thr	Thr	Thr
**A3**	D	F	W	N	I	G	M	V	Thr	Thr	Thr
**A4**	D	V	H	R	T	G	L	V	HPG	nrp	Gra
**A5**	D	I	L	Q	X	X	L	I	NO HIT	Gly	Gly
**A6**	D	V	H	R	T	G	L	V	HPG	nrp	Gra

Table S18. Predicted A-domain substrate specificity together with incorporated amino acids from the *meg* NRPS in the *P. megapolitana* genome. Gra: graminine.

	Stachelhaus Code								predicted amino acid		
									NRPS analysis[22]	Antismash	found
**A1**	D	I	L	X	L	G	V	I	Gly	Gly	Gly
**A2**	D	L	T	K	L	G	H	V	Asp	nrp	3-OH-Asp
**A3**	D	V	W	N	V	A	M	V	NO HIT	Ser	Ser
**A4**	D	F	W	N	I	G	M	V	Thr	Thr	Thr
**A5**	D	V	H	R	T	G	L	V	HPG	nrp	Gra
**A6**	D	I	N	Q	L	S	M	I	NO HIT	nrp	Ala/Ser
**A7**	D	V	H	R	T	G	L	V	HPG	nrp	Gra

Table S19. Predicted A-domain substrate specificity together with incorporated amino acids from the *plb* NRPS in the *B. plantarii* genome. Gra: graminine.

	Stachelhaus Code								predicted amino acid		
									NRPS analysis[22]	Antismash	found
**A1**	D	L	T	K	I	G	H	V	Asp	X	3-OH-Asp
**A2**	D	V	W	H	V	S	L	I	Ser	Ser	Ser
**A3**	D	V	W	H	V	S	L	I	Ser	Ser	Ser
**A4**	D	G	E	D	X	X	T	V	NO HIT	X	Orn
**A5**	D	V	H	R	T	G	L	V	HPG	X	Gra
**A6**	D	A	E	Y	L	G	T	V	Lys	X	Gln
**A7**	D	I	L	Q	L	G	L	I	Gly	Gly	Gly
**A8**	D	V	H	R	T	G	L	V	HPG	X	Gra

Table S20. Predicted A-domain substrate specificity together with incorporated amino acids from the *plb* homologous NRPS in the *B. gladioli* pv. agaricicola genome. Differences to the codes extracted from modules in the *plb* cluster are highlighted in red. Gra: graminine.

	Stachelhaus Code								predicted amino acid		
									NRPS analysis[22]	Antismash	found
**A1**	D	L	T	K	I	G	H	V	Asp	Asp	3-OH-Asp
**A2**	D	V	W	H	V	S	L	I	Ser	Ser	Ser
**A3**	D	V	W	H	V	S	L	I	Ser	Ser	Ser
**A4**	D	G	E	D	X	X	T	V	NO HIT	Orn	Orn
**A5**	D	V	H	R	T	G	L	V	HPG	X	Gra
**A6**	D	A	E	D	T	G	T	V	Arg	Lys	Lys
**A7**	D	I	L	Q	L	G	L	I	Gly	Gly	Gly
**A8**	D	V	H	A	T	G	L	V	HPG	X	Gra

Spectra of New Compounds

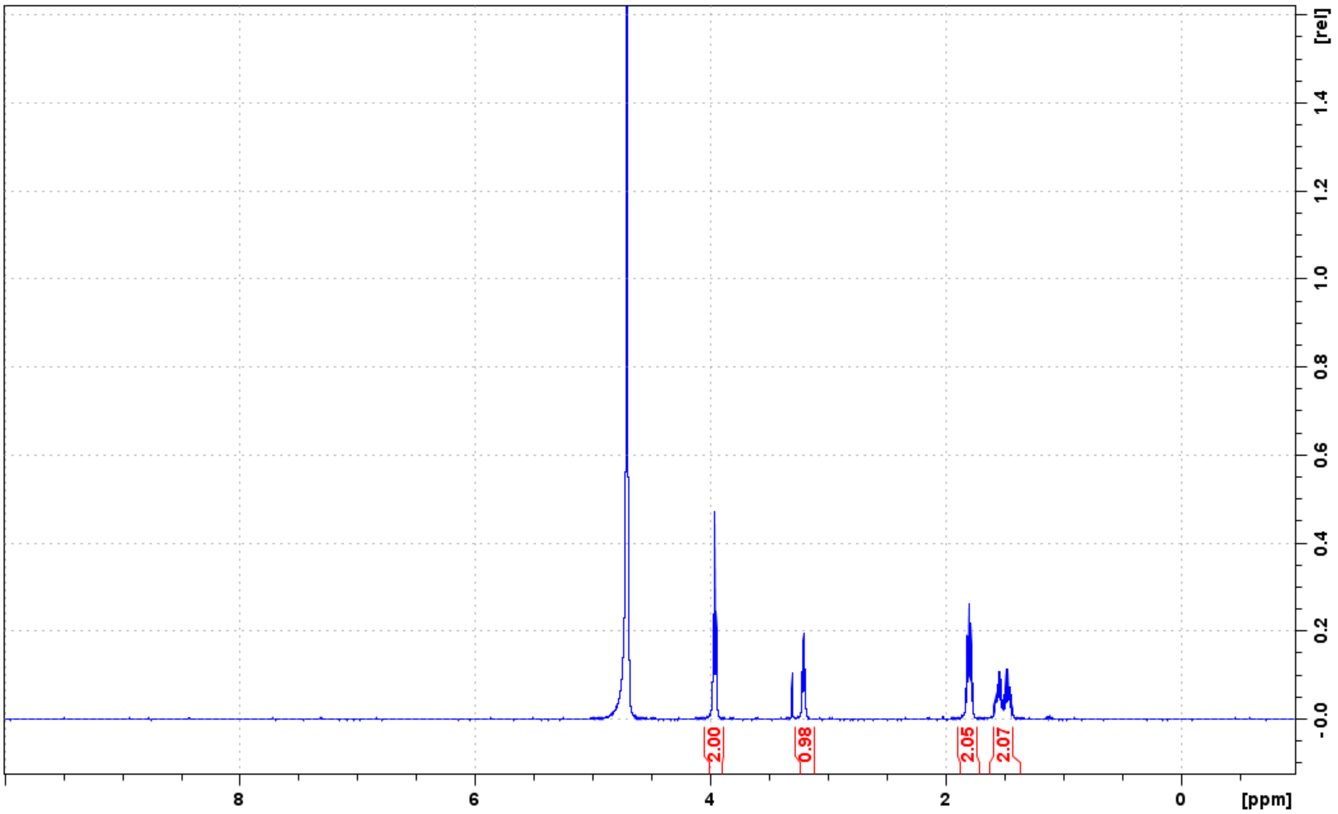

Figure S32. ^{1}H NMR Spectrum of L-graminine (500 MHz, 298K, D_2O/NaOD).

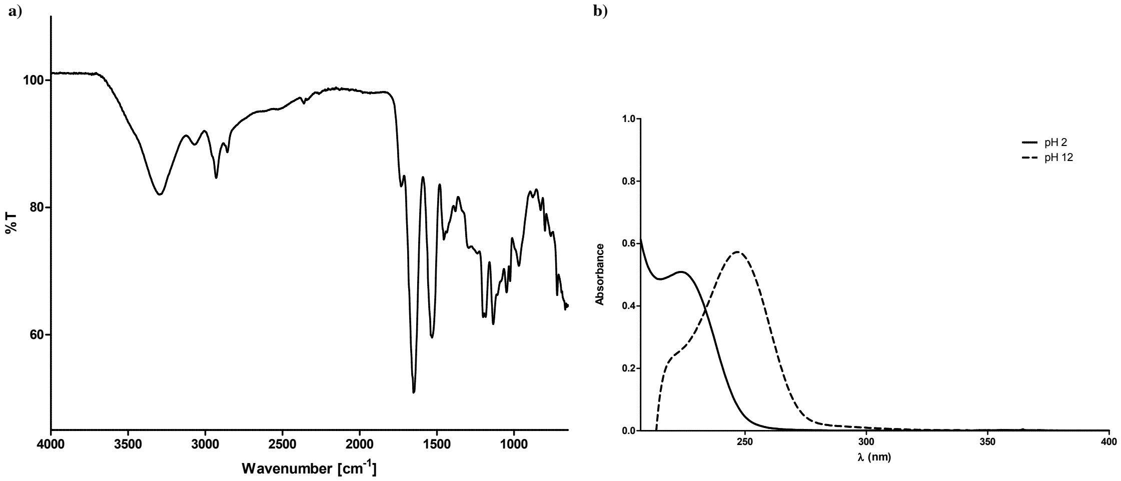

Figure S33. IR spectrum (a) and UV spectrum (b) of megapolibactin A.

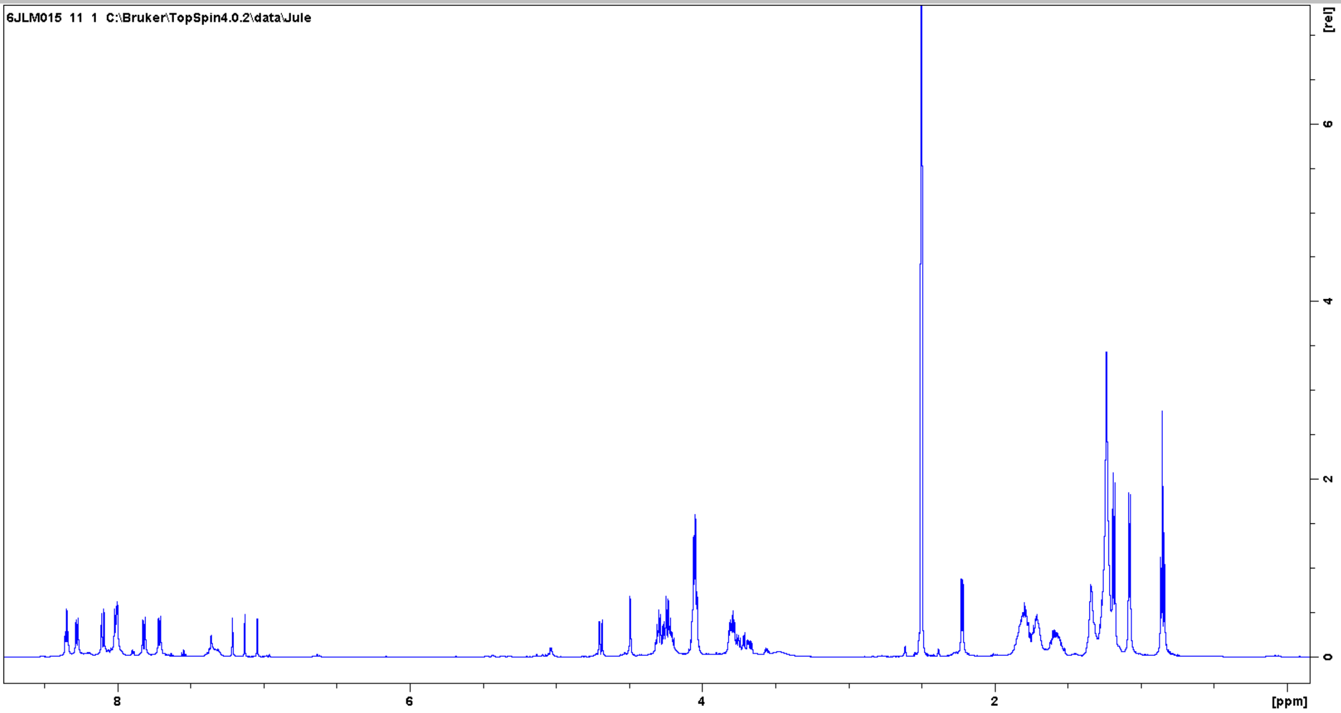

Figure S34. ^{1}H NMR spectrum of megapolibactin A (600 MHz, 298K, DMSO-d_6).

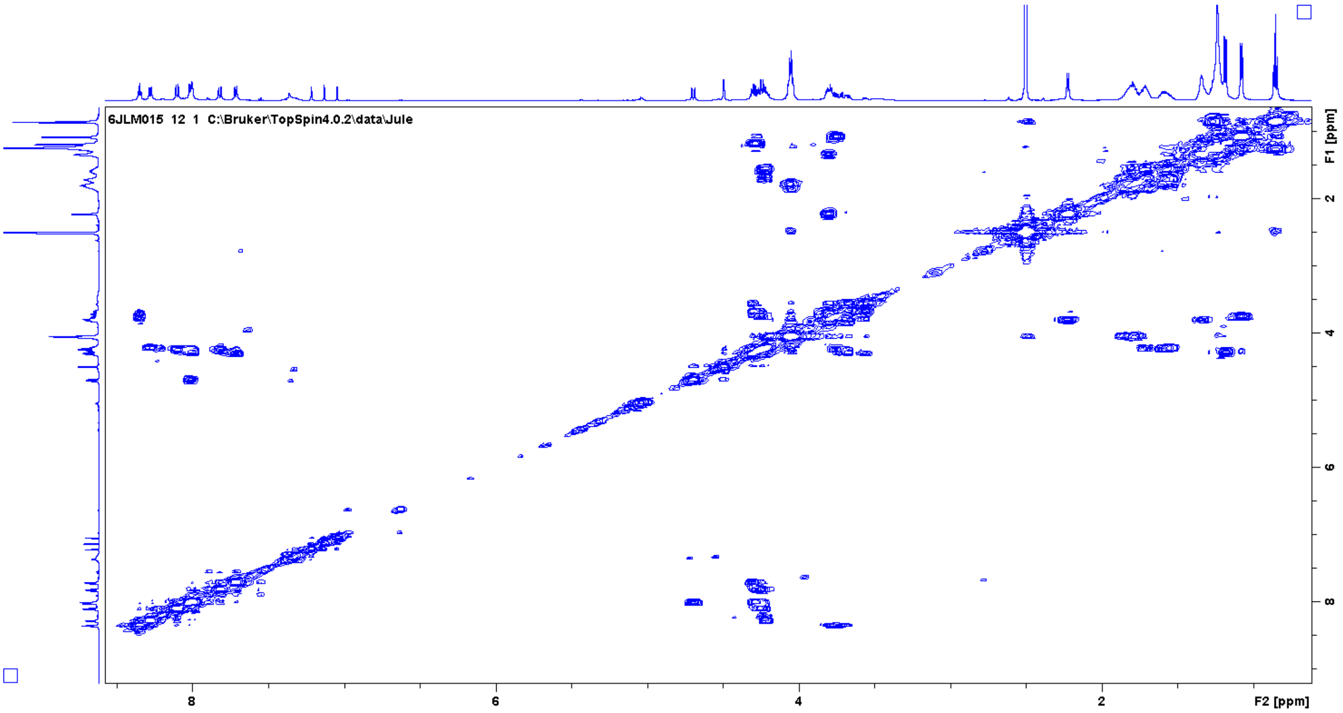

Figure S35. ^{1}H-^{1}H-COSY spectrum of megapolibactin A (600 MHz, 298K, DMSO-d_6).

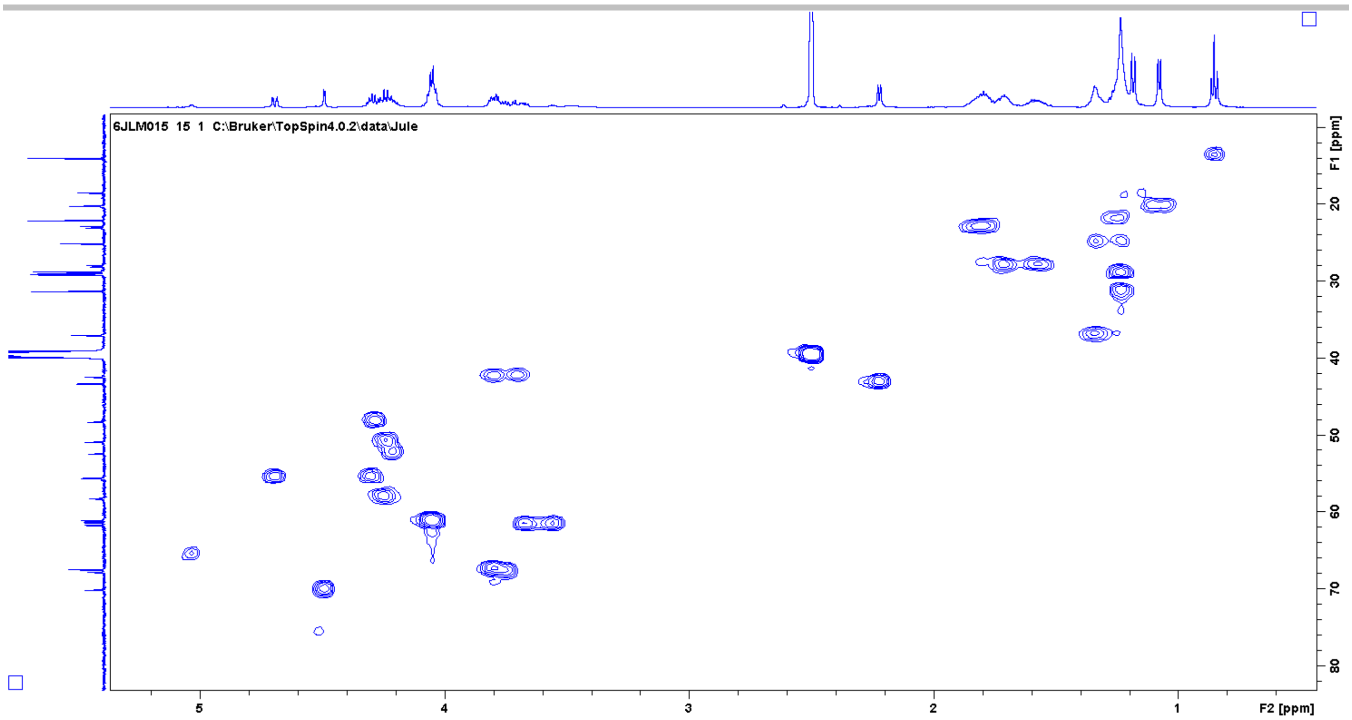

Figure S36. HSQC spectrum of megapolibactin A (600 MHz, 298K, DMSO-d_6).

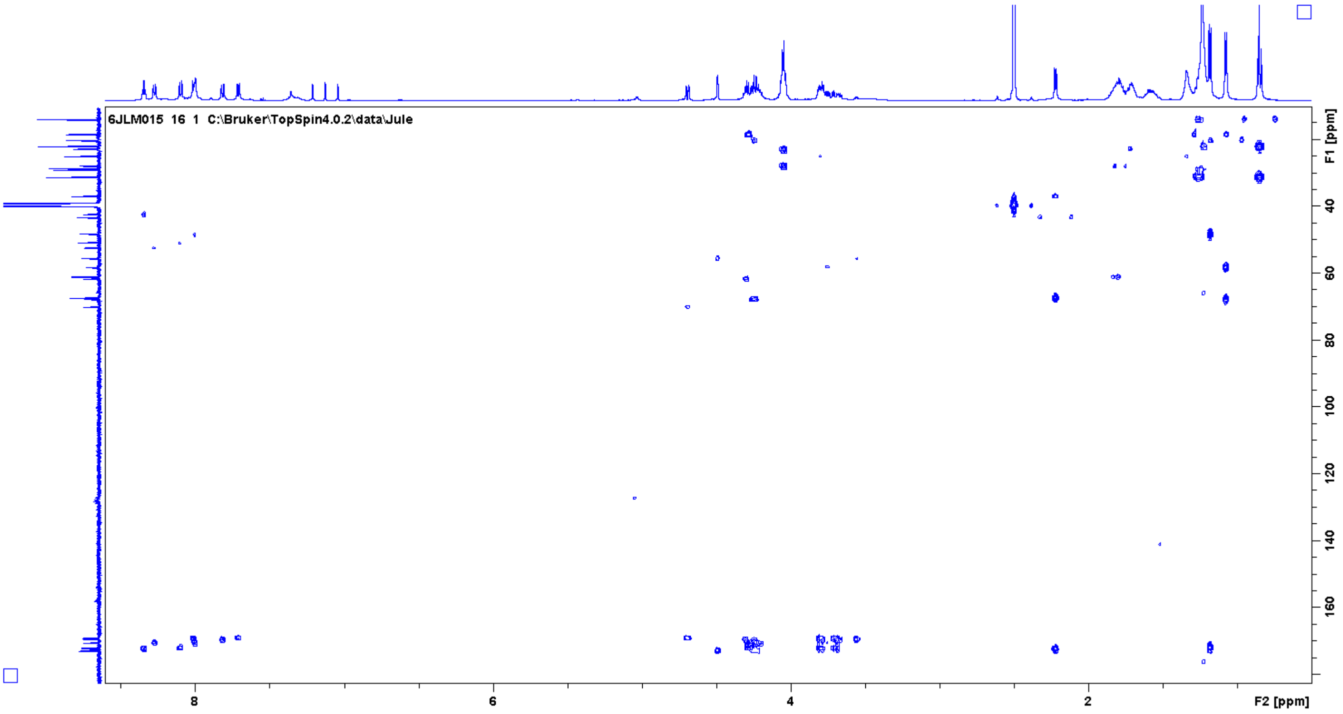

Figure S37. HMBC spectrum of megapolibactin A (600 MHz, 298K, DMSO-d_6).

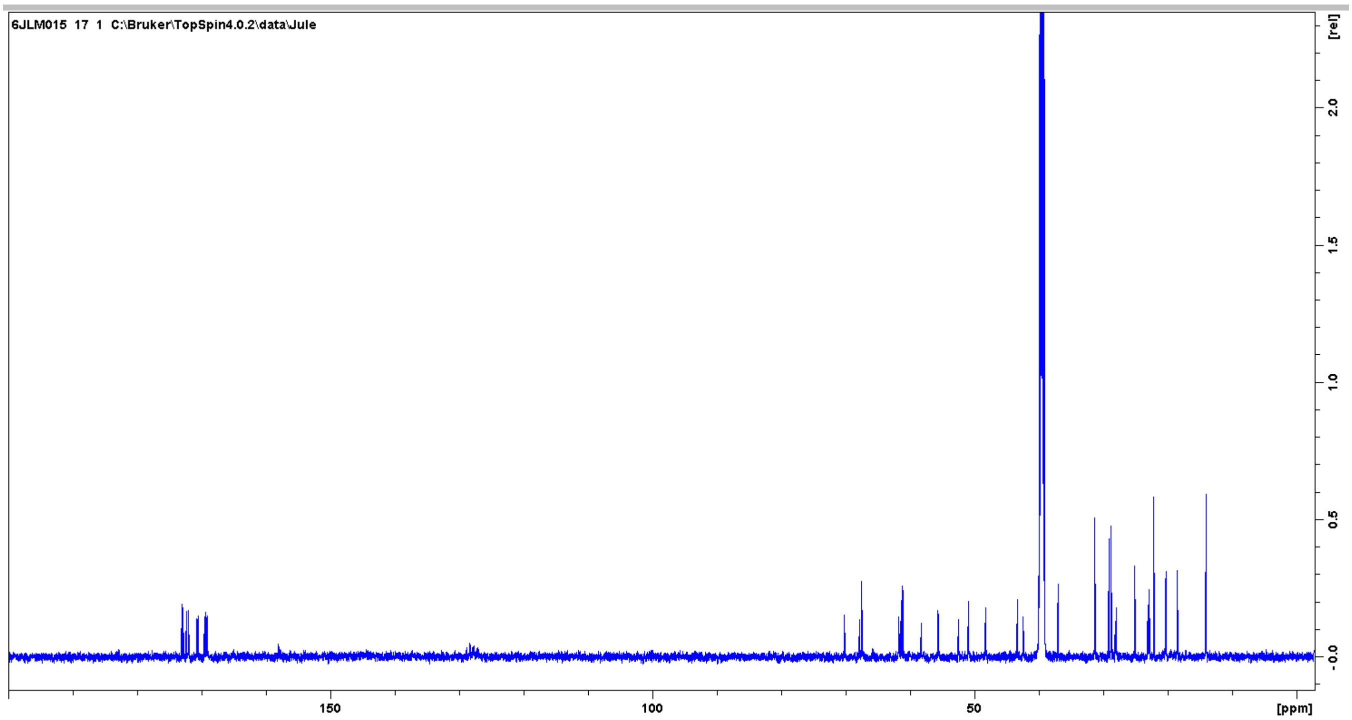

Figure S38. ^{13}C NMR spectrum of megapolibactin A (150 MHz, 298K, DMSO-d_6).

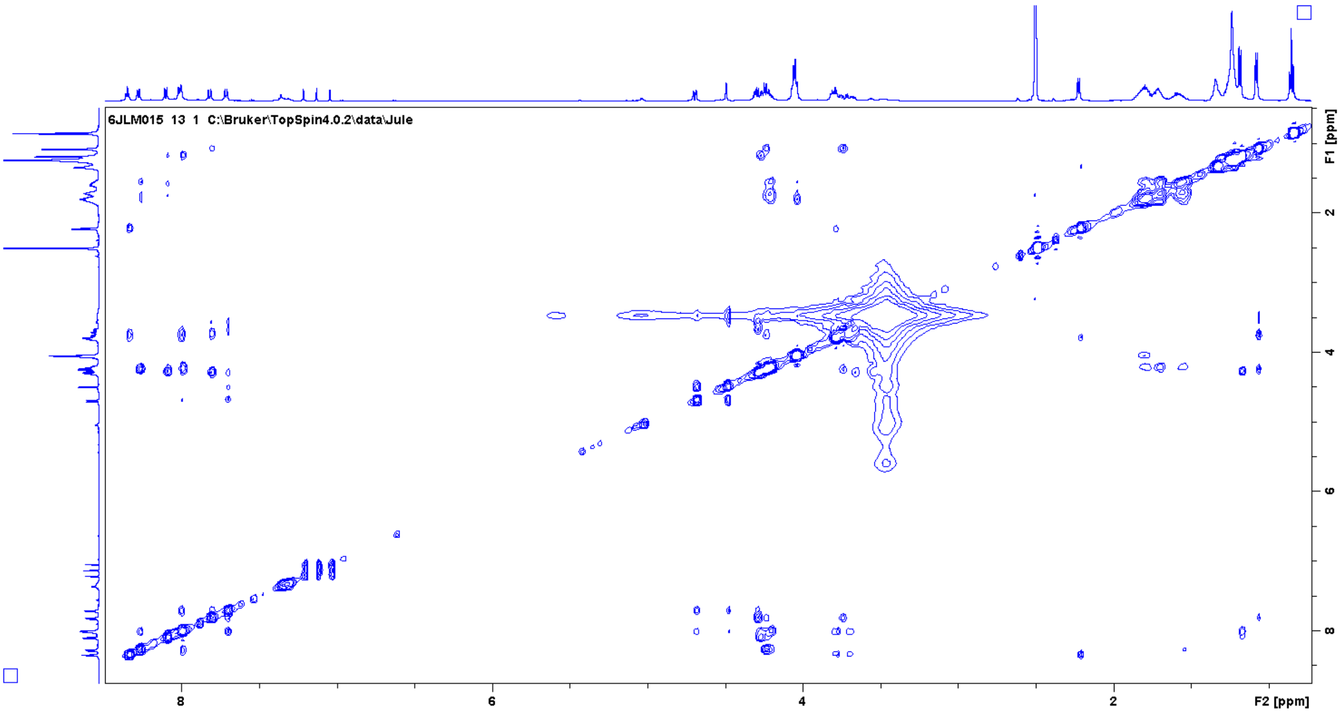

Figure S39. NOESY spectrum of megapolibactin A (600 MHz, 298K, DMSO-d_6).

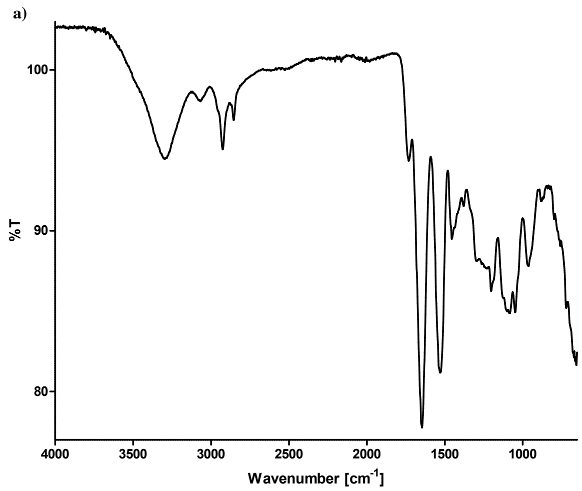

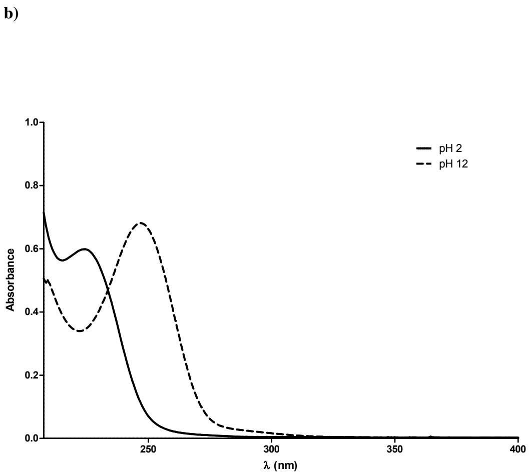

Figure S40. IR spectrum (a) and UV spectrum (b) of megapolibactin B.

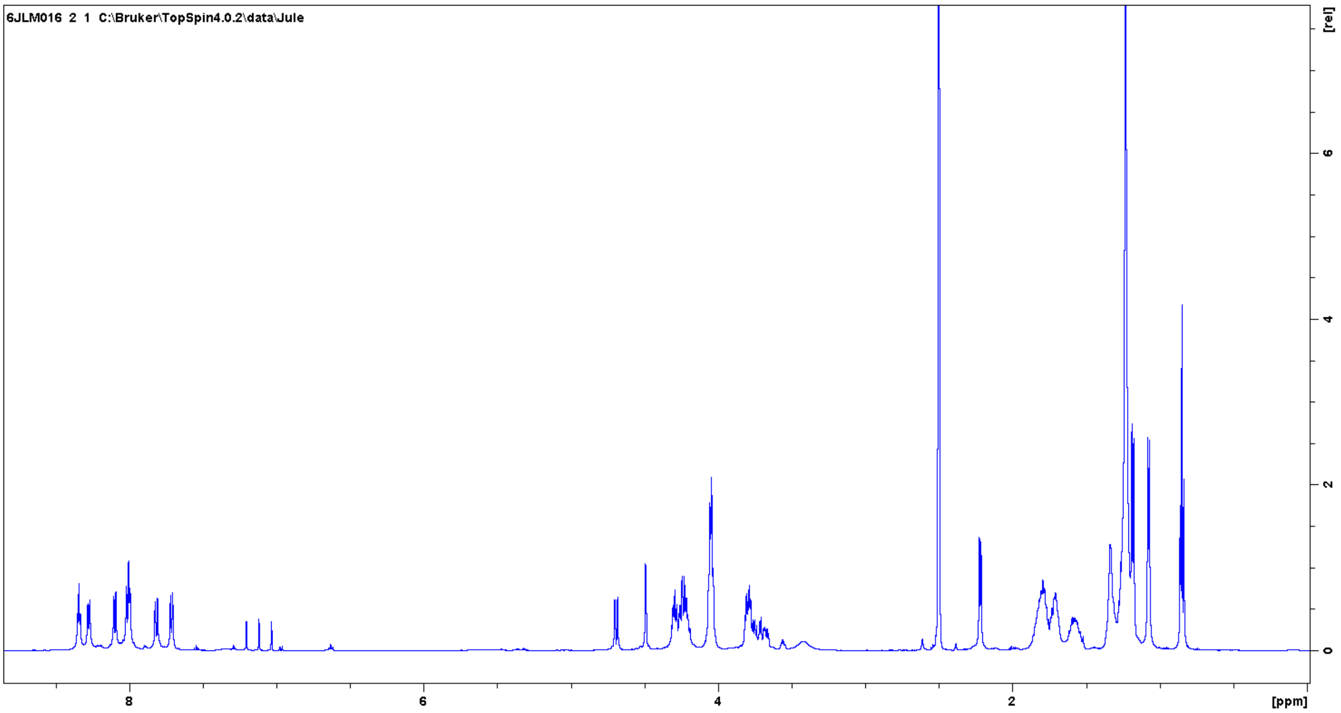

Figure S41. ^{1}H NMR spectrum of megapolibactin B (600 MHz, 298K, DMSO-d_6).

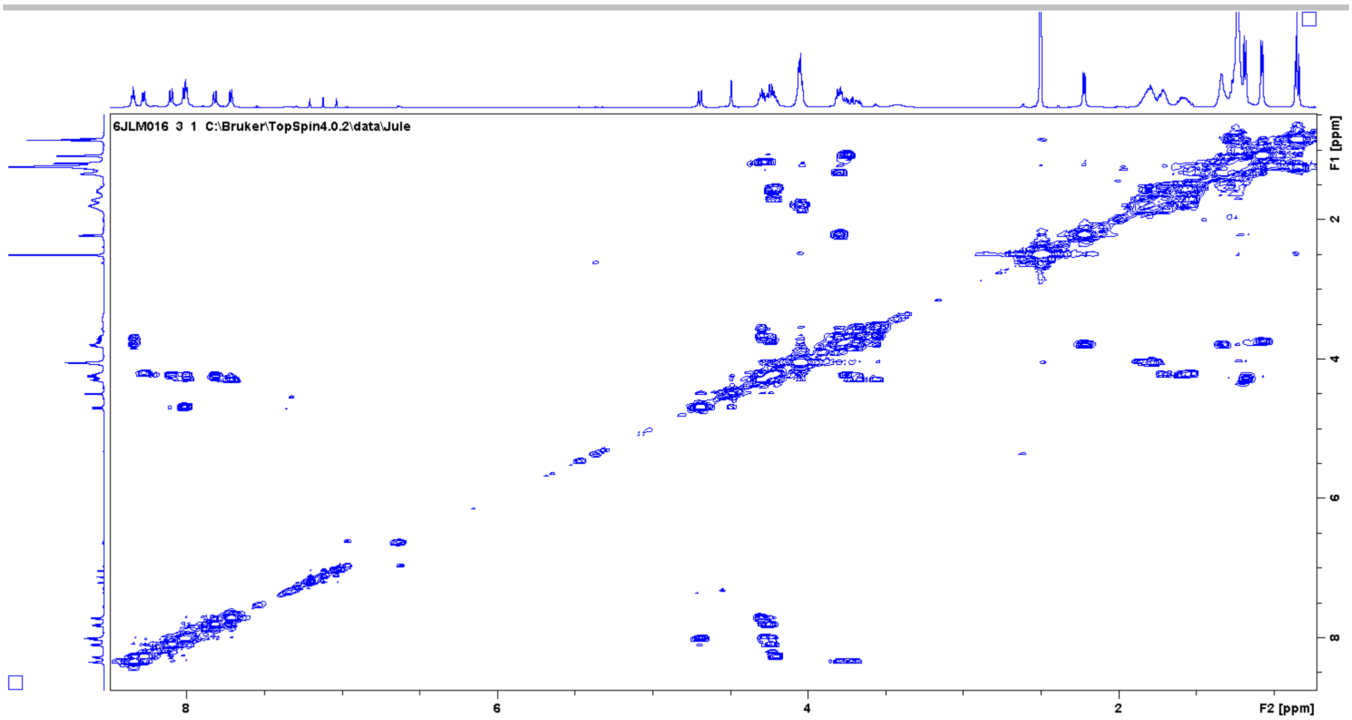

Figure S42. ^{1}H-^{1}H-COSY spectrum of megapolibactin B (600 MHz, 298K, DMSO-d_6).

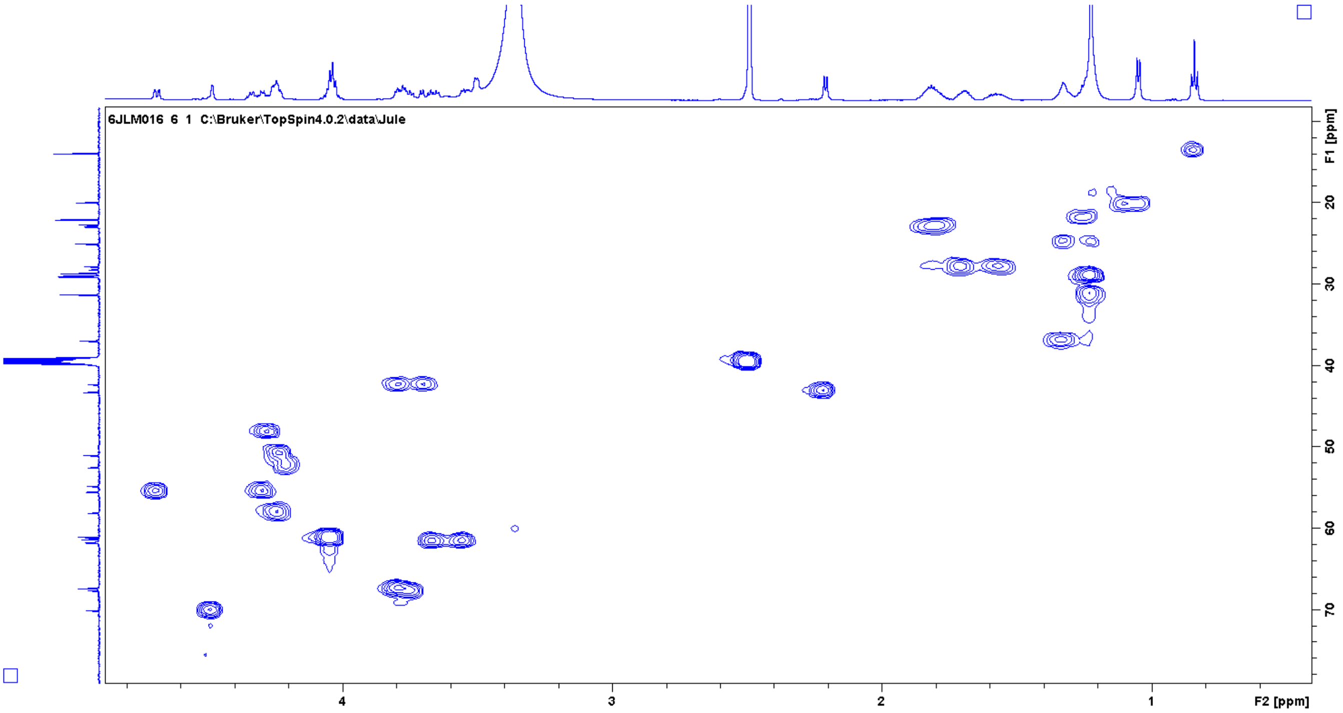

Figure S43. HSQC spectrum of megapolibactin B (600 MHz, 298K, DMSO-d_6).

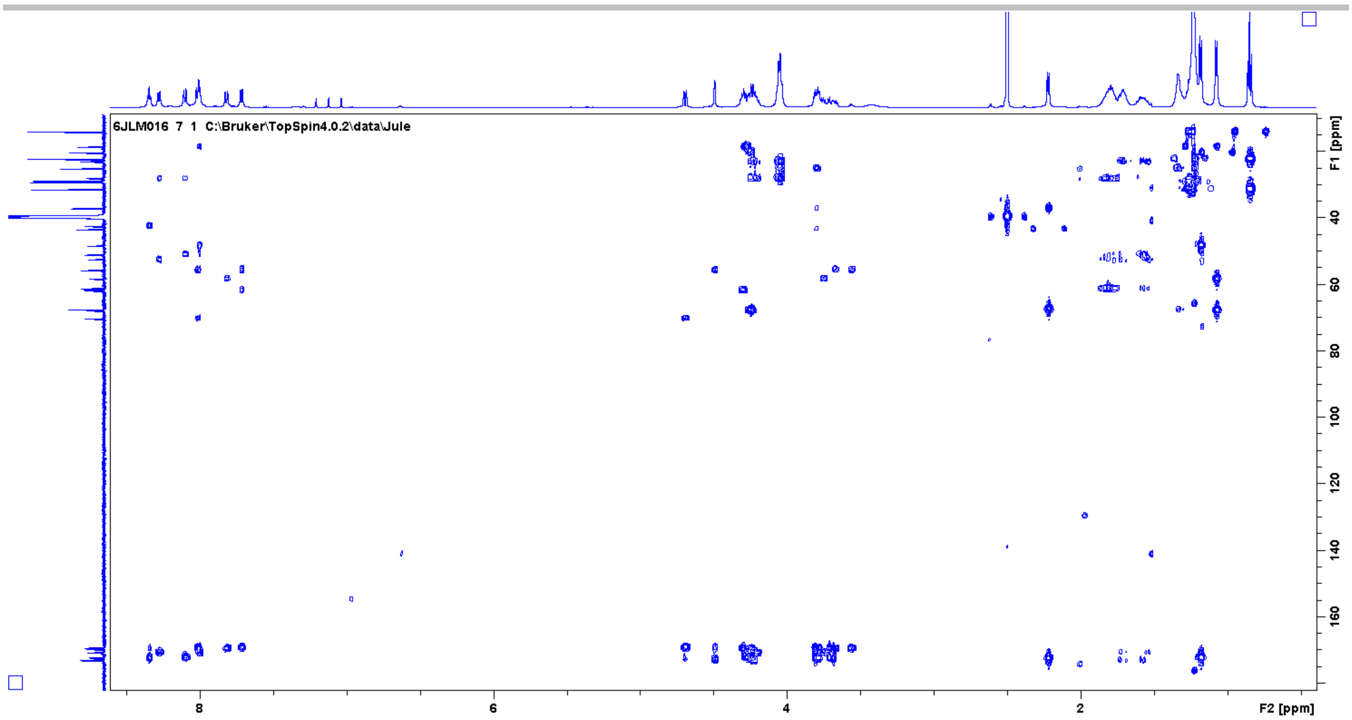

Figure S44. HMBC spectrum of megapolibactin B (600 MHz, 298K, DMSO-d_6).

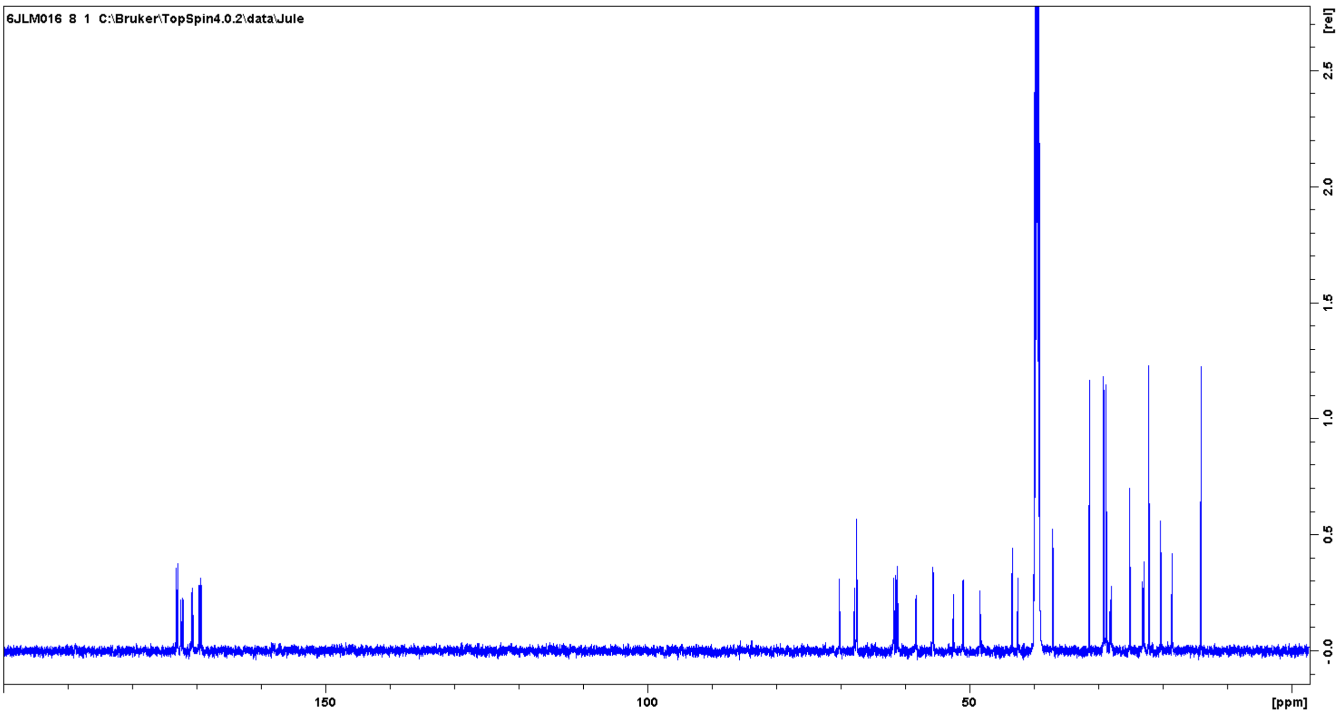

Figure S45. ^{13}C NMR spectrum of megapolibactin B (150 MHz, 298K, DMSO-d_6).

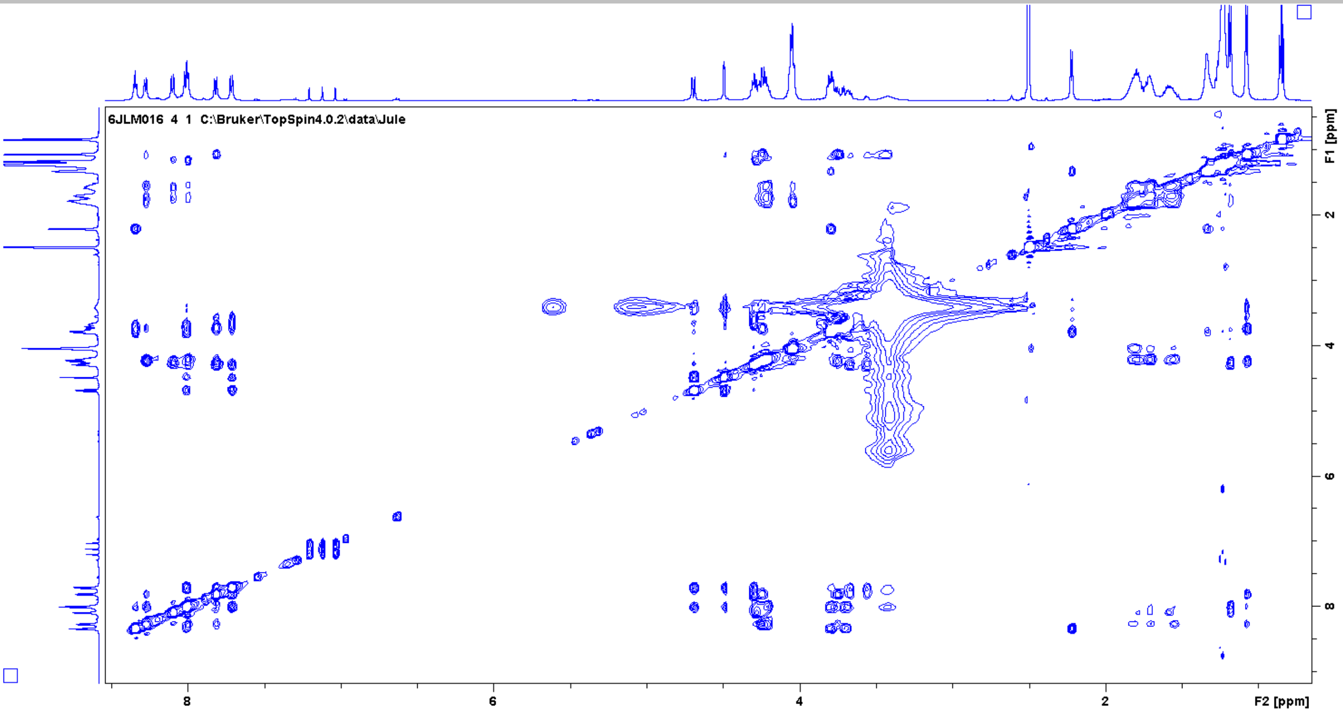

Figure S46. NOESY spectrum of megapolibactin B (600 MHz, 298K, DMSO-d_6).

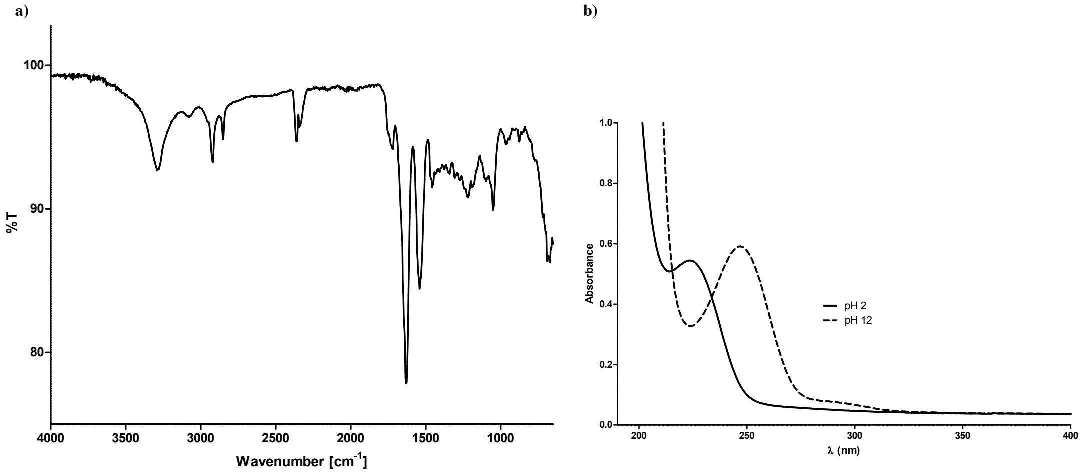

Figure S47. IR spectrum (a) and UV spectrum (b) of megapolibactin C.

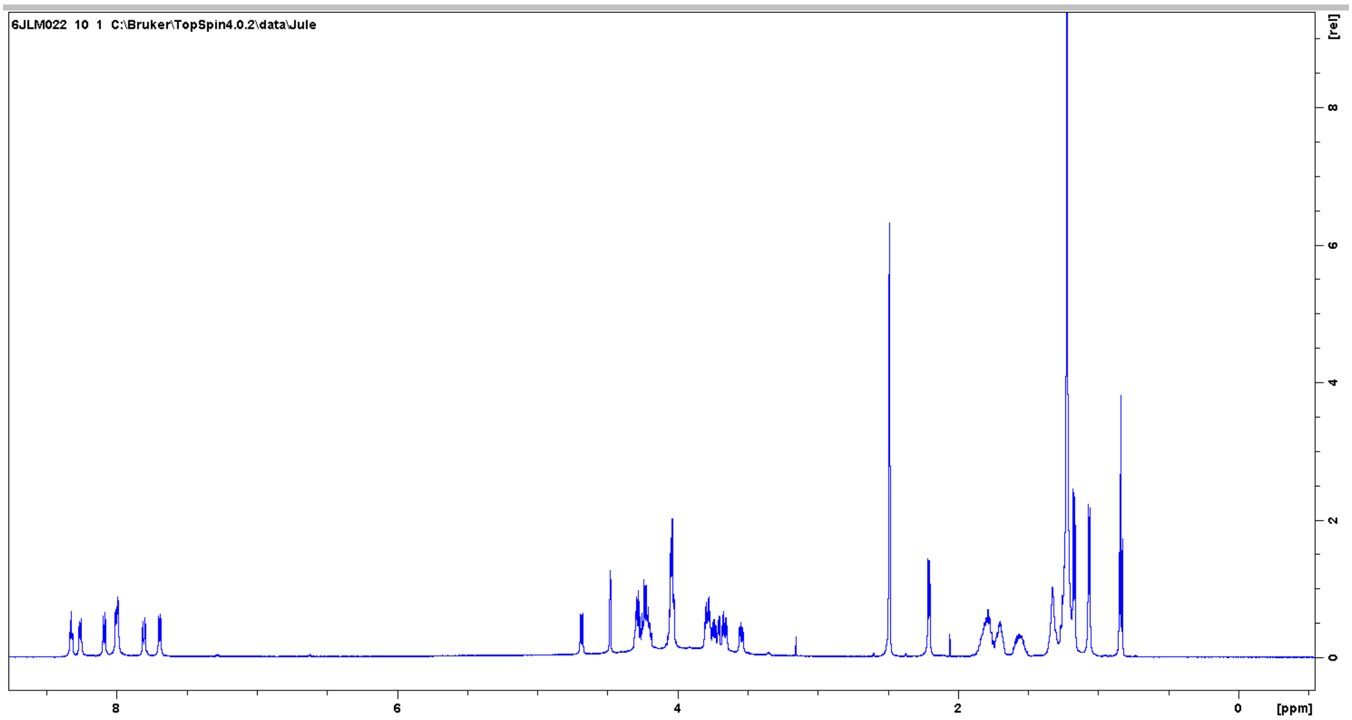

Figure S48. ^{1}H NMR spectrum of megapolibactin C (600 MHz, 298K, DMSO-d_6).

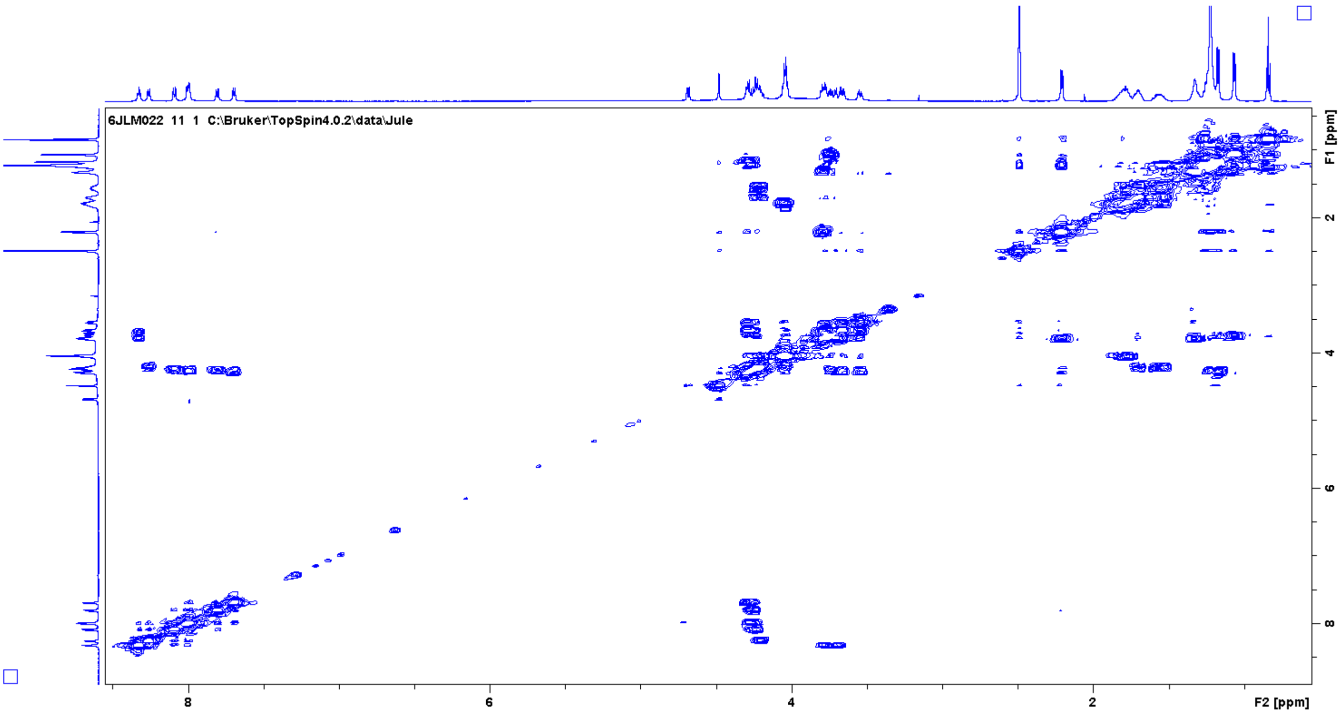

Figure S49. ^{1}H-^{1}H-COSY spectrum of megapolibactin C (600 MHz, 298K, DMSO-d_6).

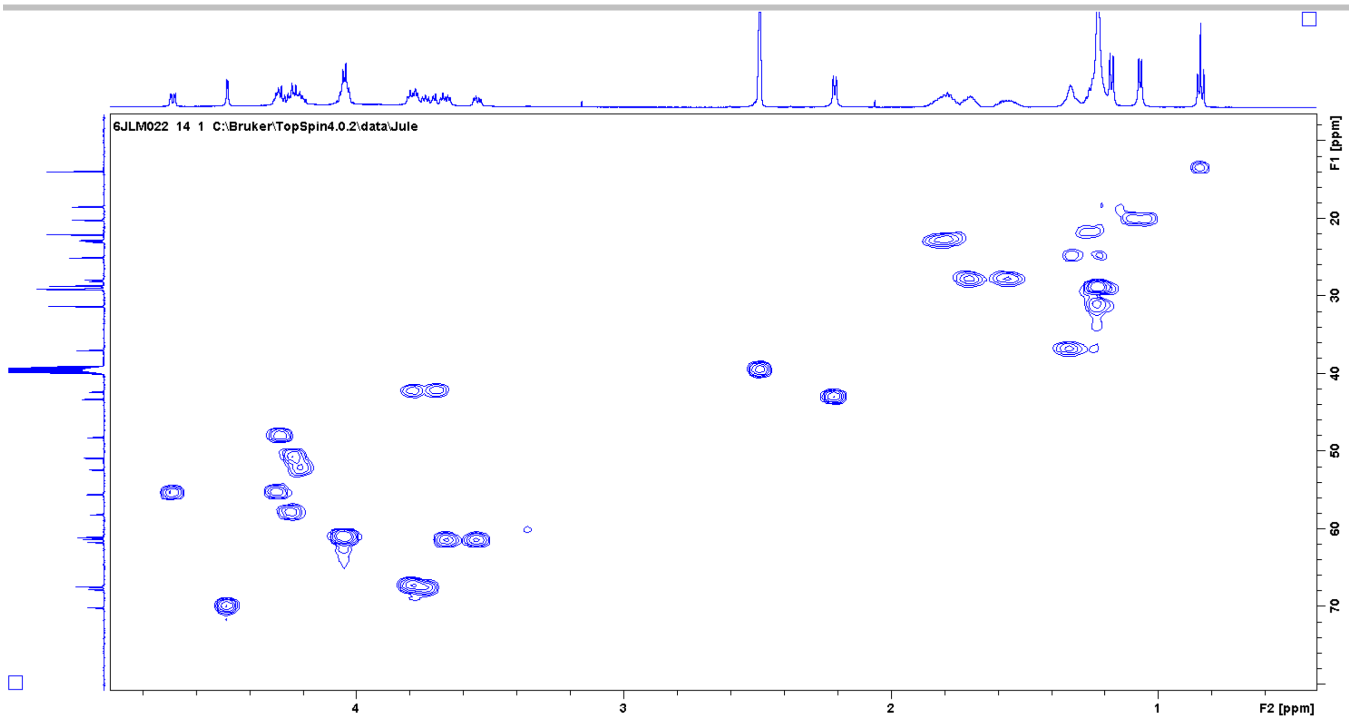

Figure S50. HSQC spectrum of megapolibactin C (600 MHz, 298K, DMSO-d_6).

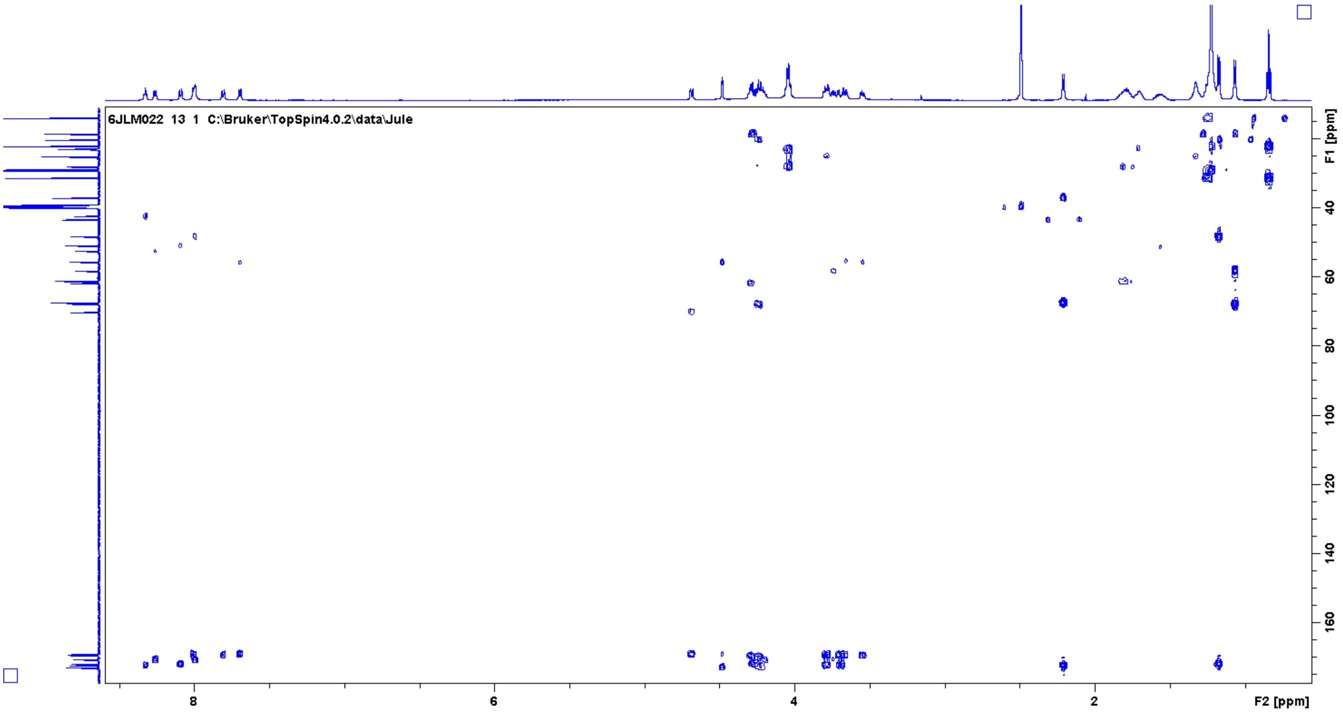

Figure S51. HMBC spectrum of megapolibactin C (600 MHz, 298K, DMSO-d_6).

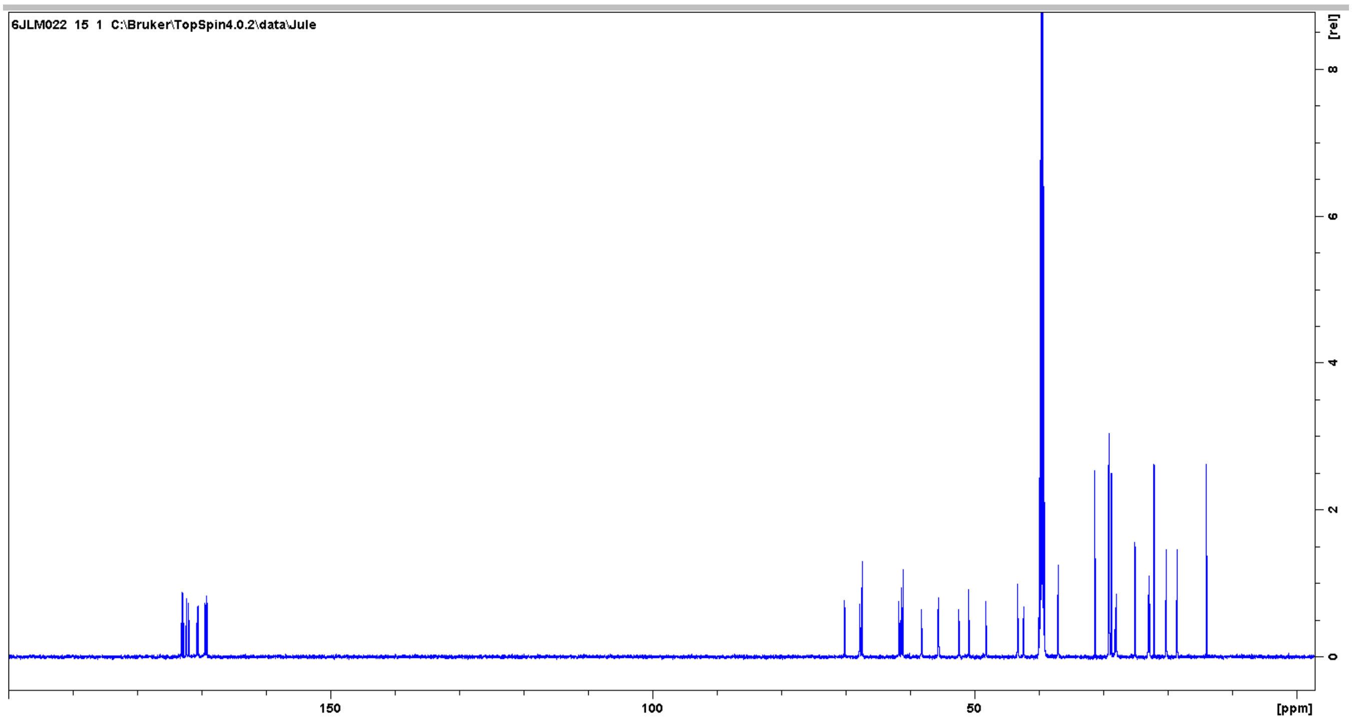

Figure S52. ^{13}C NMR spectrum of megapolibactin C (150 MHz, 298K, DMSO-d_6).

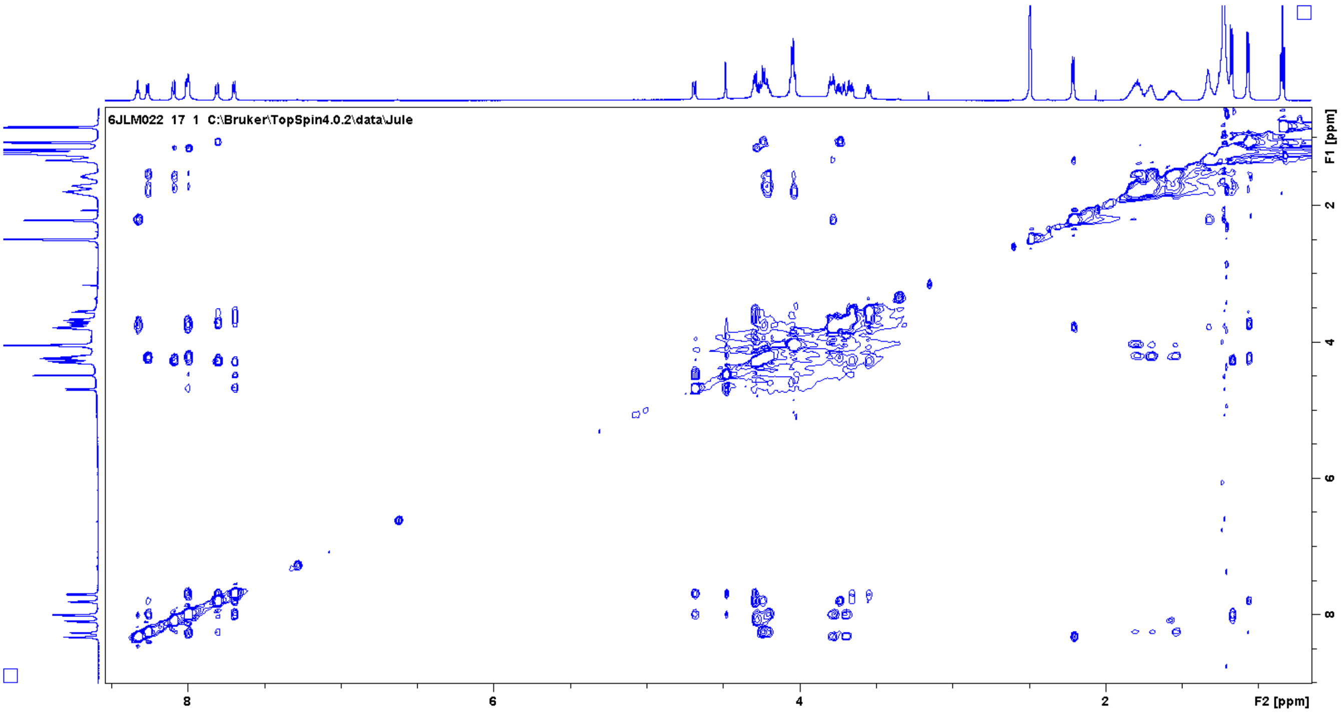

Figure S53. NOESY spectrum of megapolibactin C (600 MHz, 298K, DMSO-d_6).

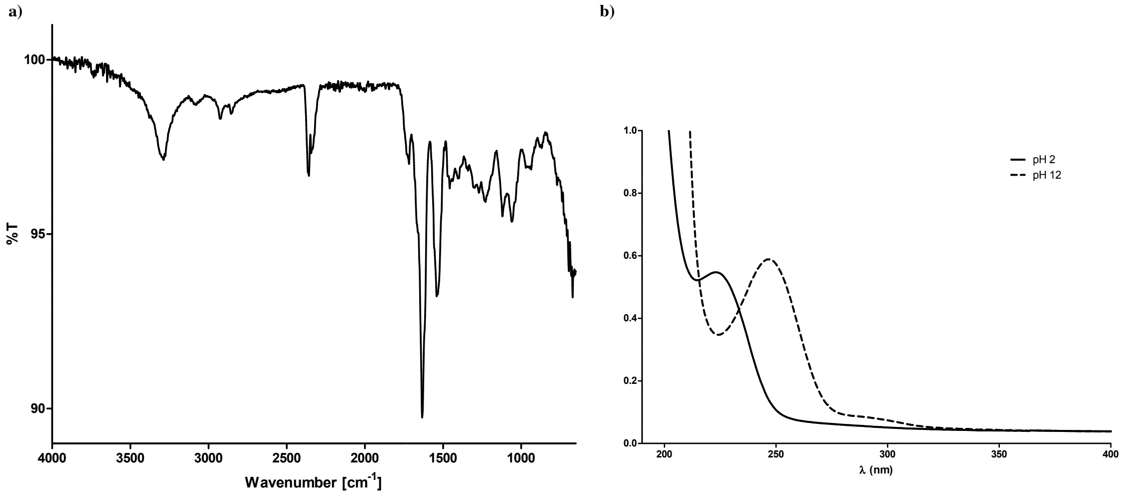

Figure S54. IR spectrum (**a**) and UV spectrum (**b**) of megapolibactin E.

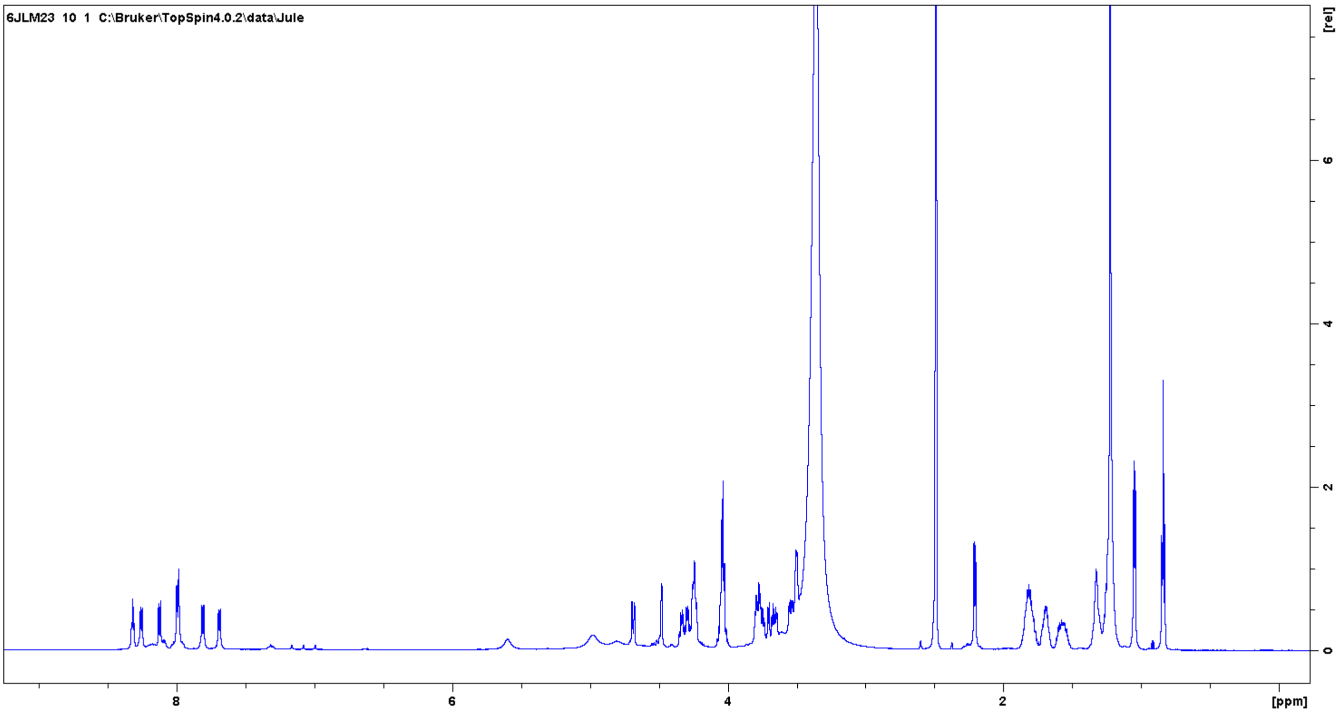

Figure S55. ^{1}H NMR spectrum of megapolibactin E (600 MHz, 298K, DMSO-d_6).

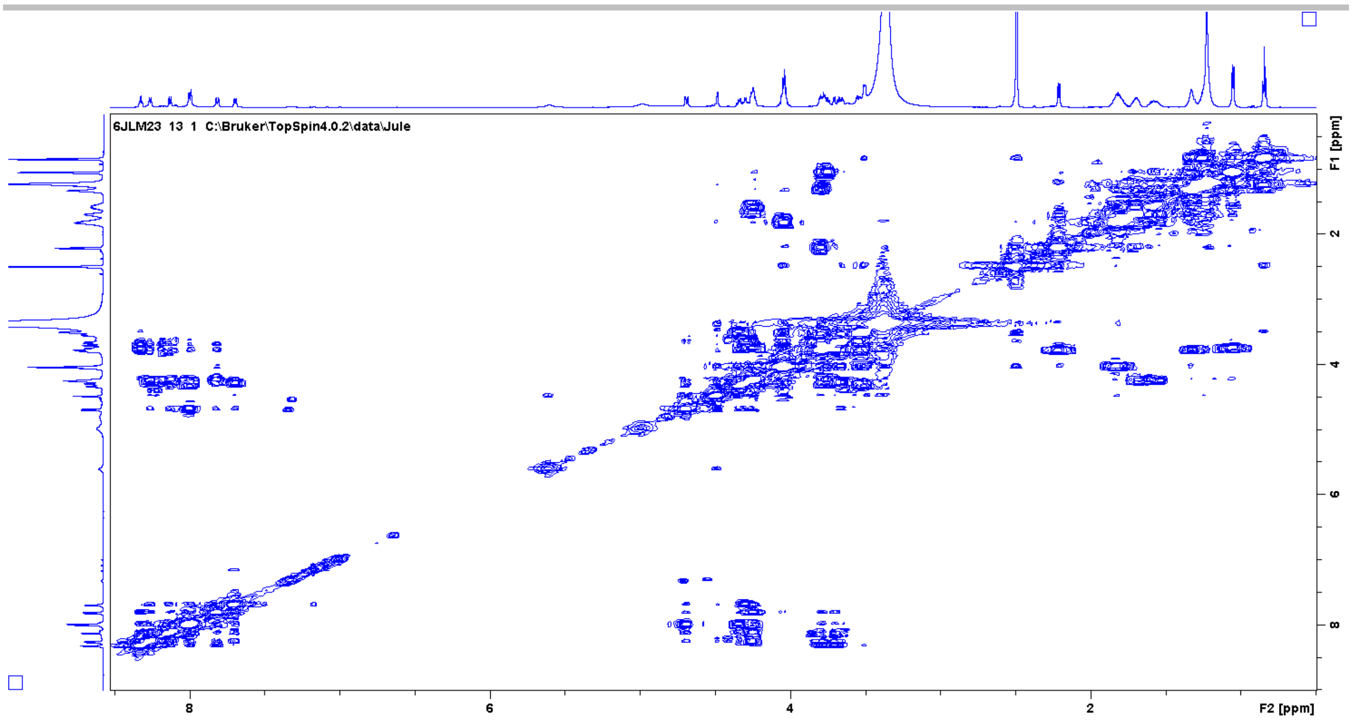

Figure S56. ^{1}H-^{1}H-COSY spectrum of megapolibactin E (600 MHz, 298K, DMSO-d_6).

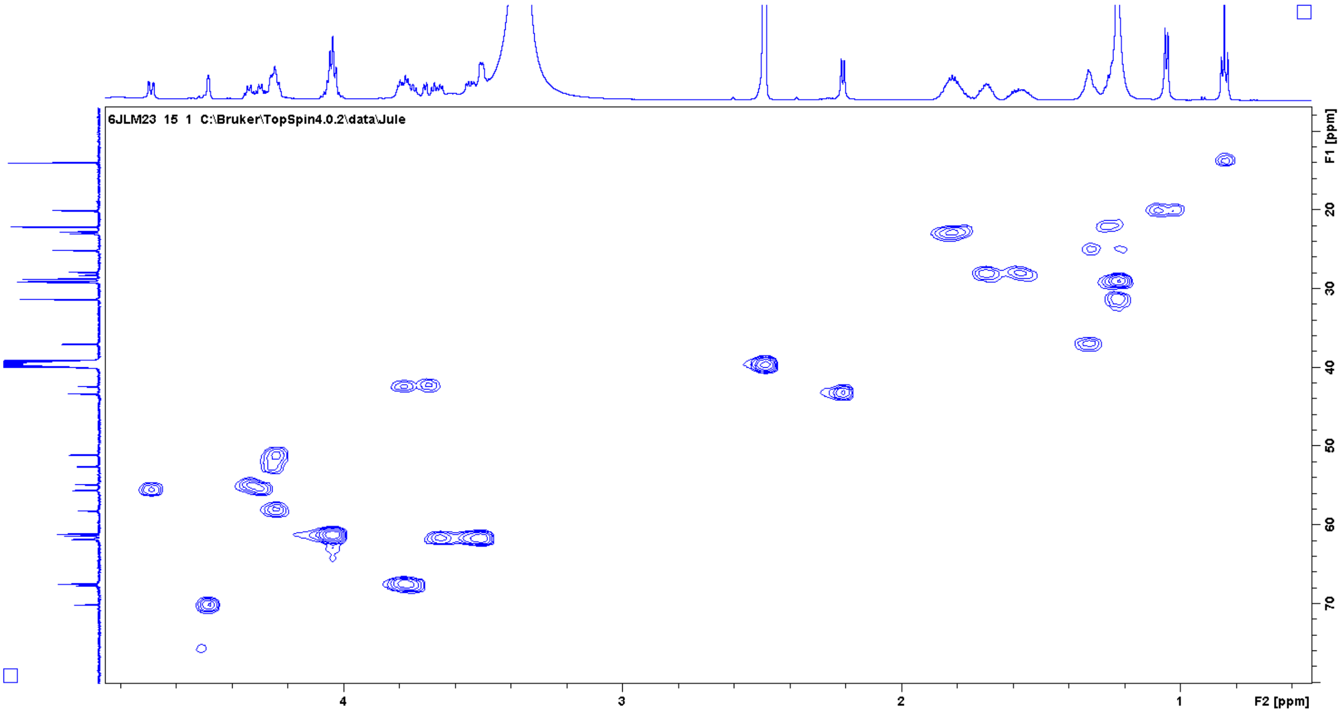

Figure S57. HSQC spectrum of megapolibactin E (600 MHz, 298K, DMSO-d_6).

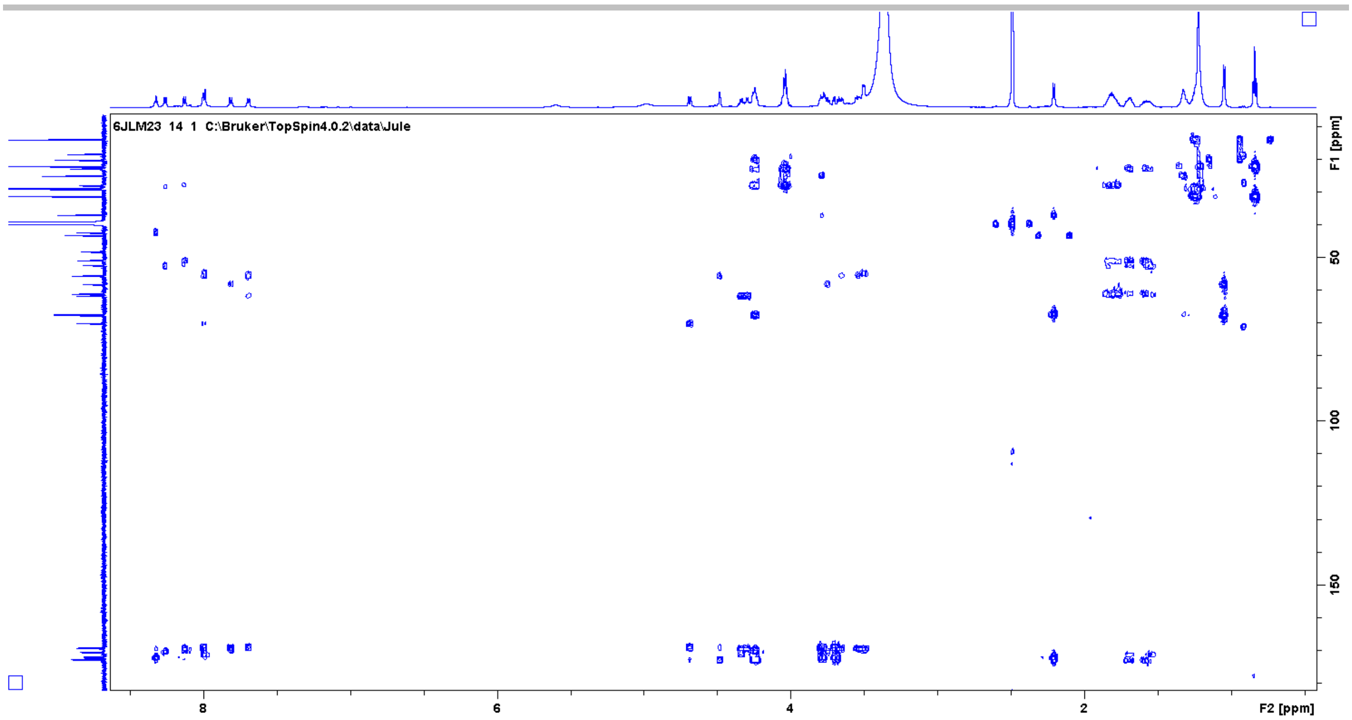

Figure S58. HMBC spectrum of megapolibactin E (600 MHz, 298K, DMSO-d_6).

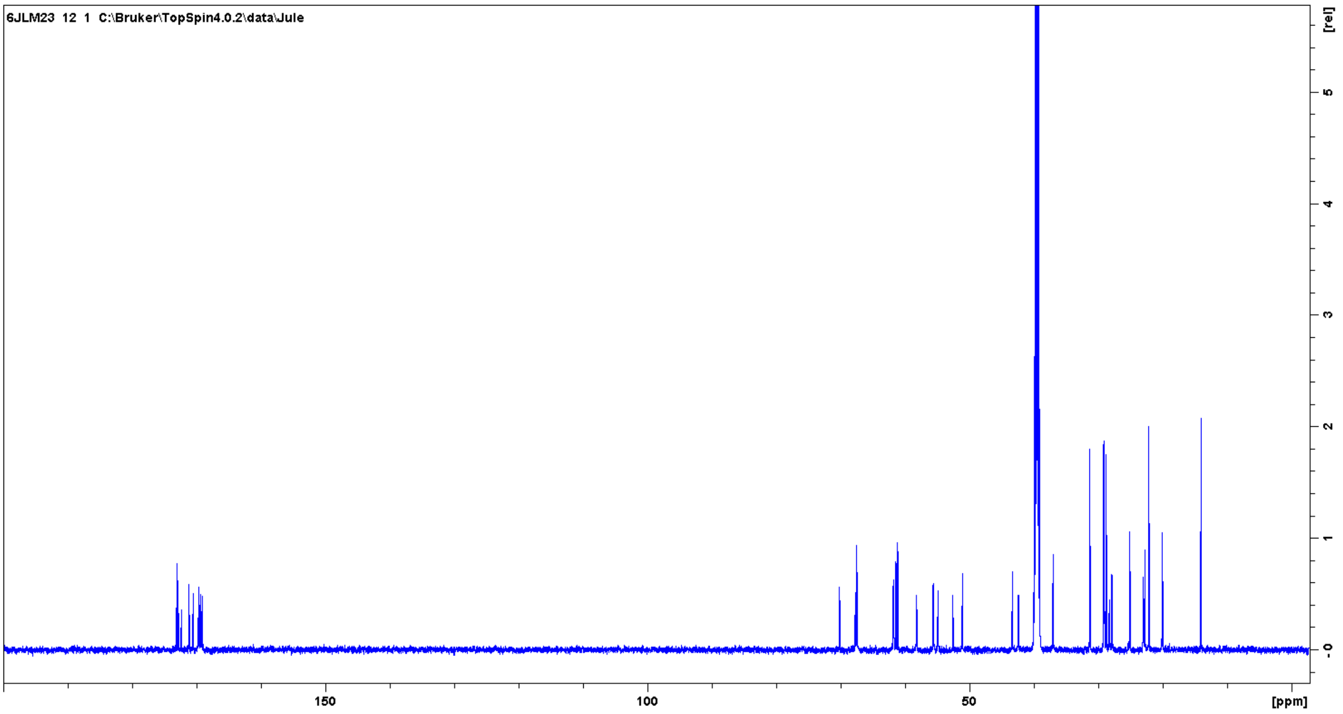

Figure S59. ^{13}C NMR spectrum of megapolibactin E (150 MHz, 298K, DMSO-d_6).

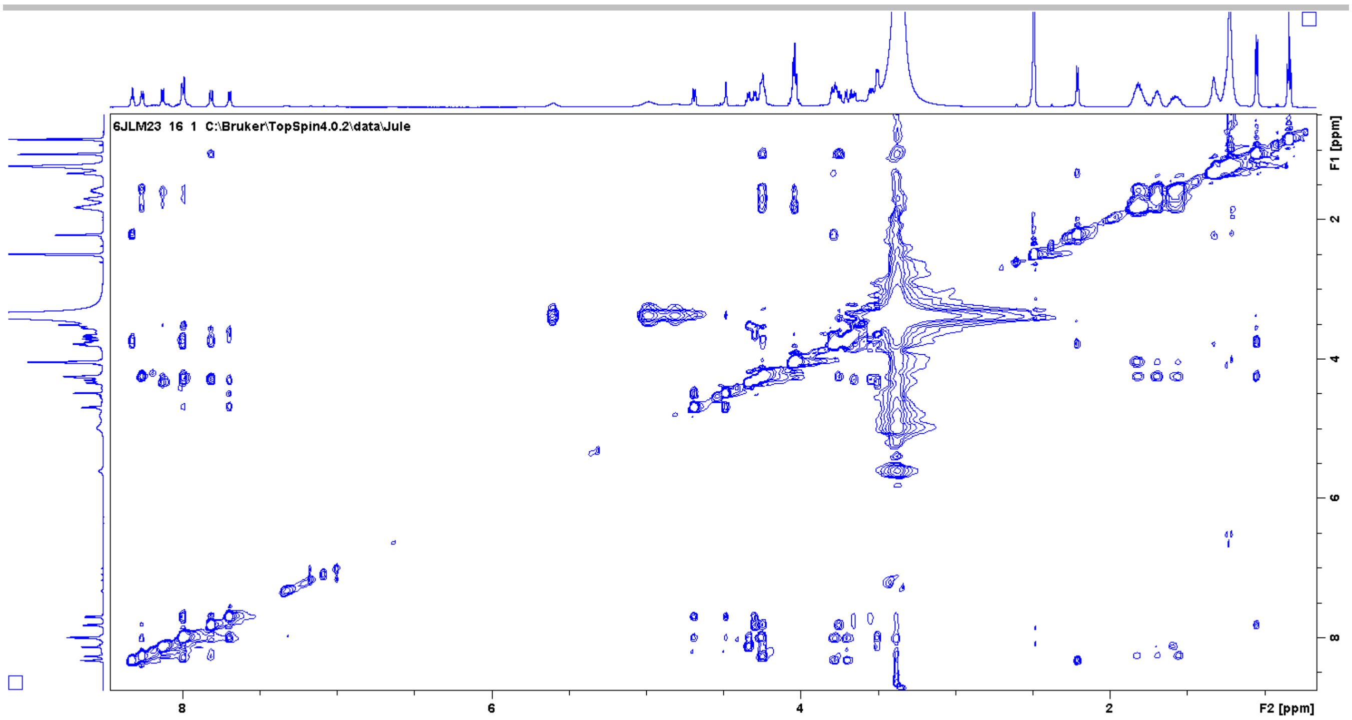

Figure S60. NOESY spectrum of megapolibactin E (600 MHz, 298K, DMSO-d_6).

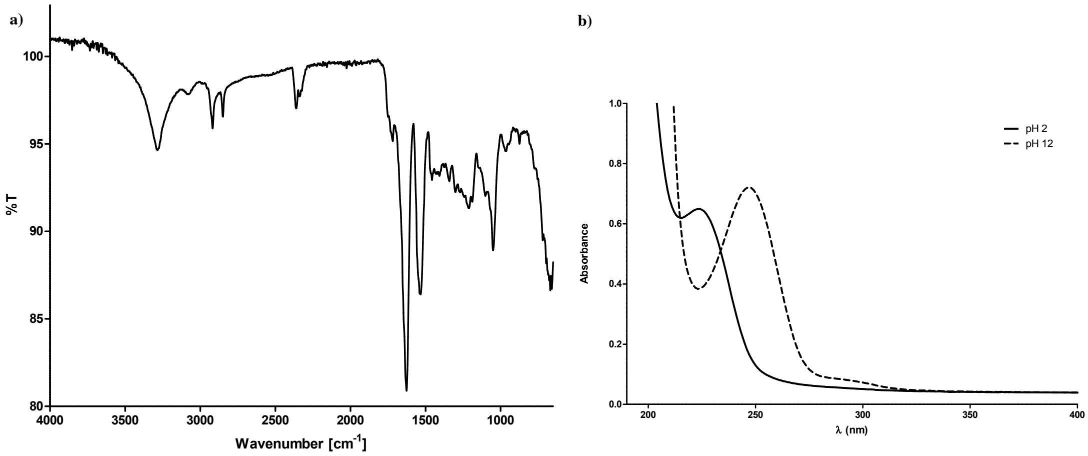

Figure S61. IR spectrum (a) and UV spectrum (b) of megapolibactin F.

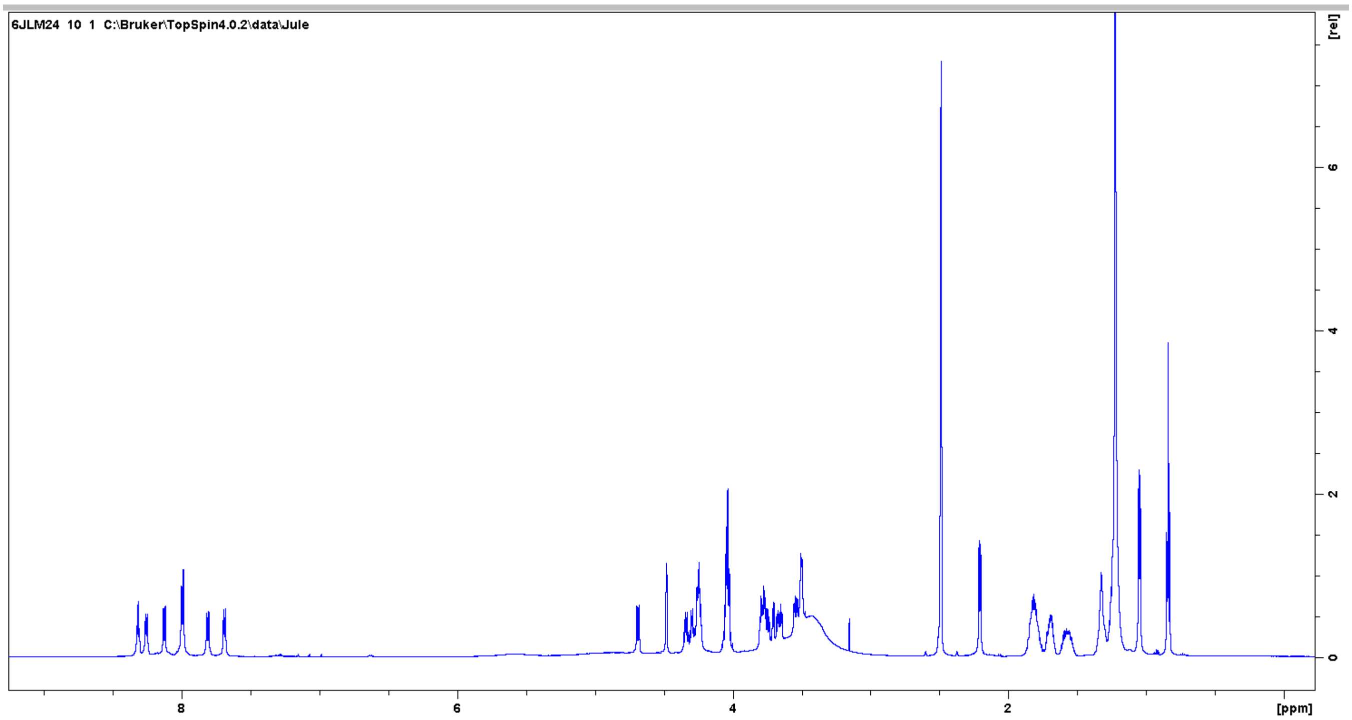

Figure S62. ^{1}H NMR spectrum of megapolibactin F (600 MHz, 298K, DMSO-d_6).

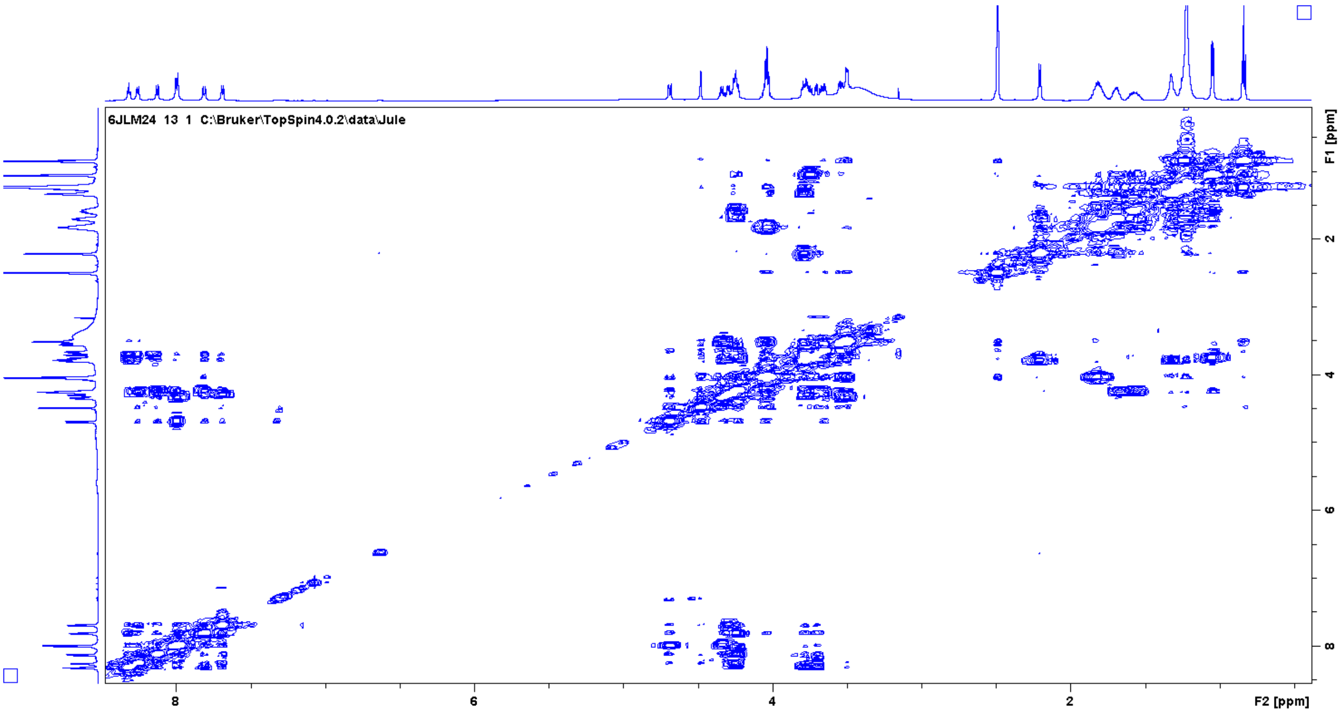

Figure S63. ^{1}H-^{1}H-COSY spectrum of megapolibactin F (600 MHz, 298K, DMSO-d_6).

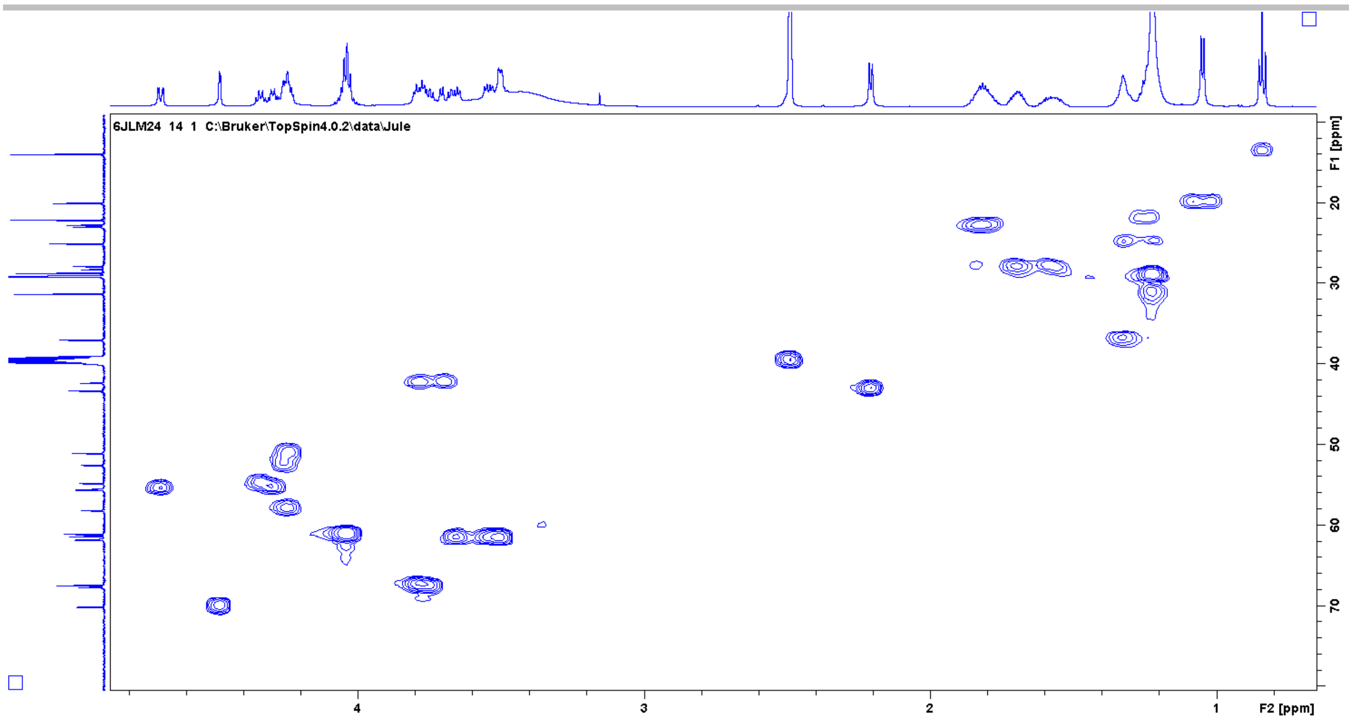

Figure S64. HSQC spectrum of megapolibactin F (600 MHz, 298K, DMSO-d_6).

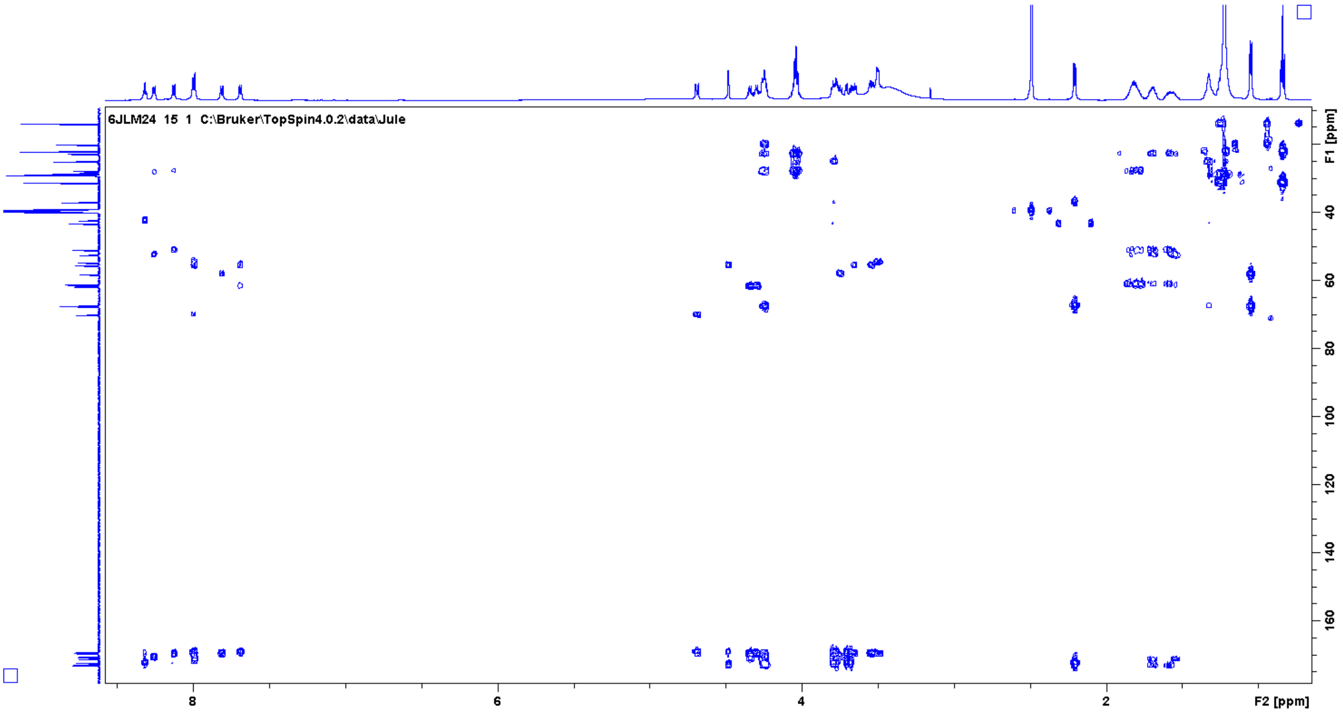

Figure S65. HMBC spectrum of megapolibactin F (600 MHz, 298K, DMSO-d_6).

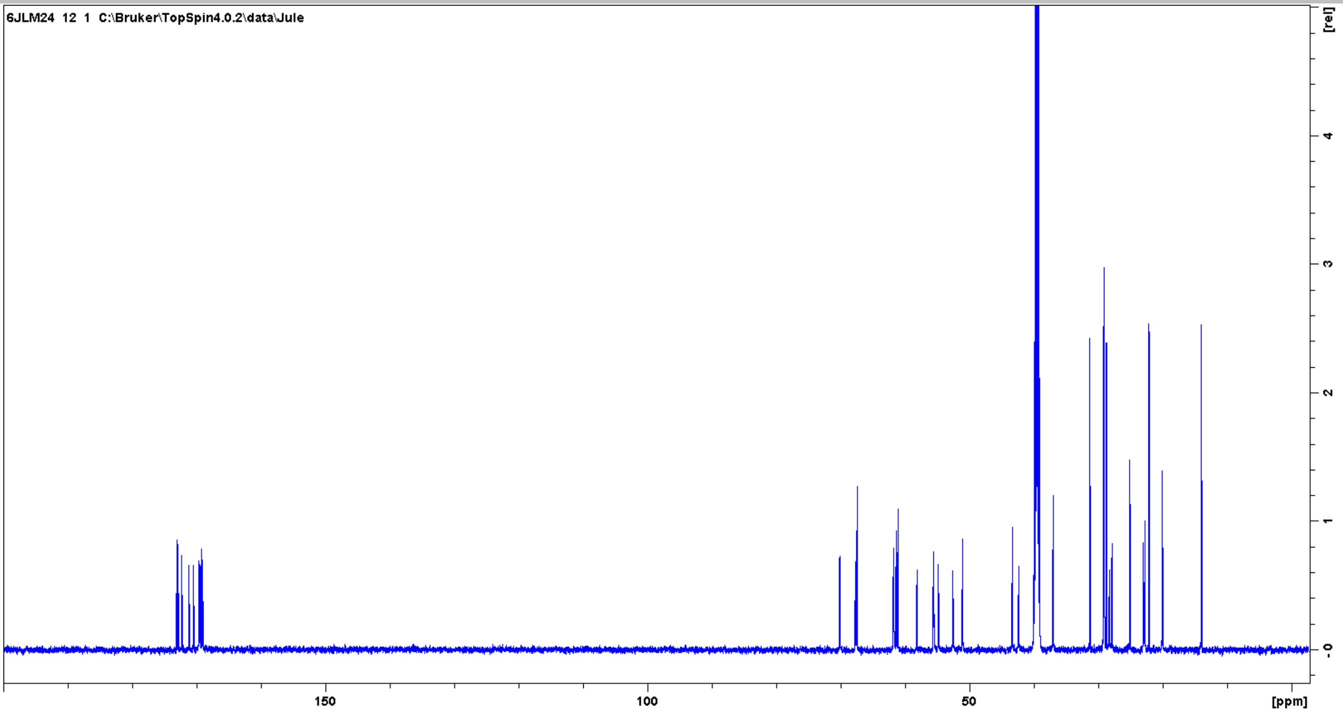

Figure S66. ^{13}C NMR spectrum of megapolibactin F (150 MHz, 298K, DMSO-d_6).

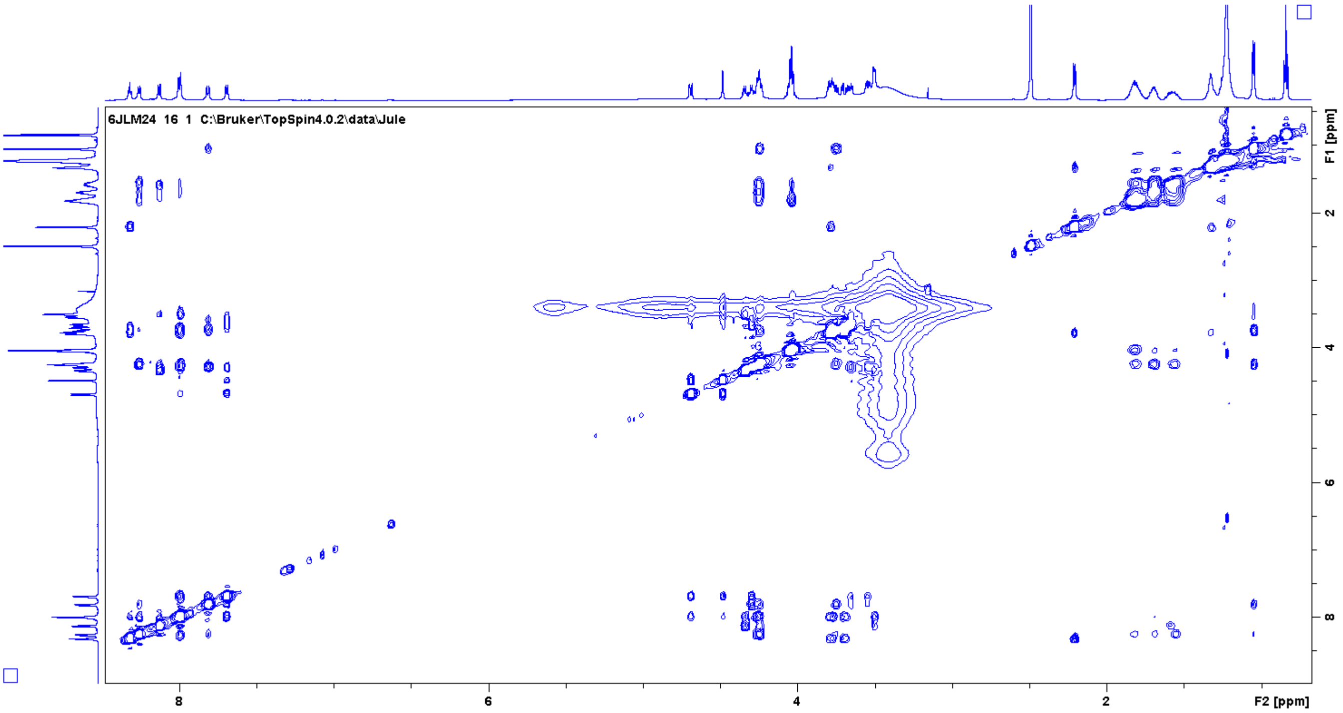

Figure S67. NOESY spectrum of megapolibactin F (600 MHz, 298K, DMSO-d_6).

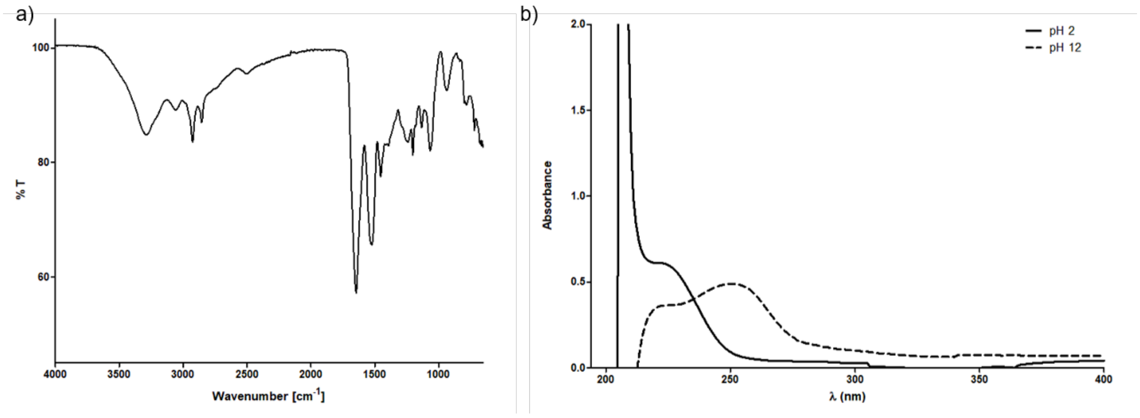

Figure S68. IR spectrum (a) and UV spectrum (b) of plantaribactin.

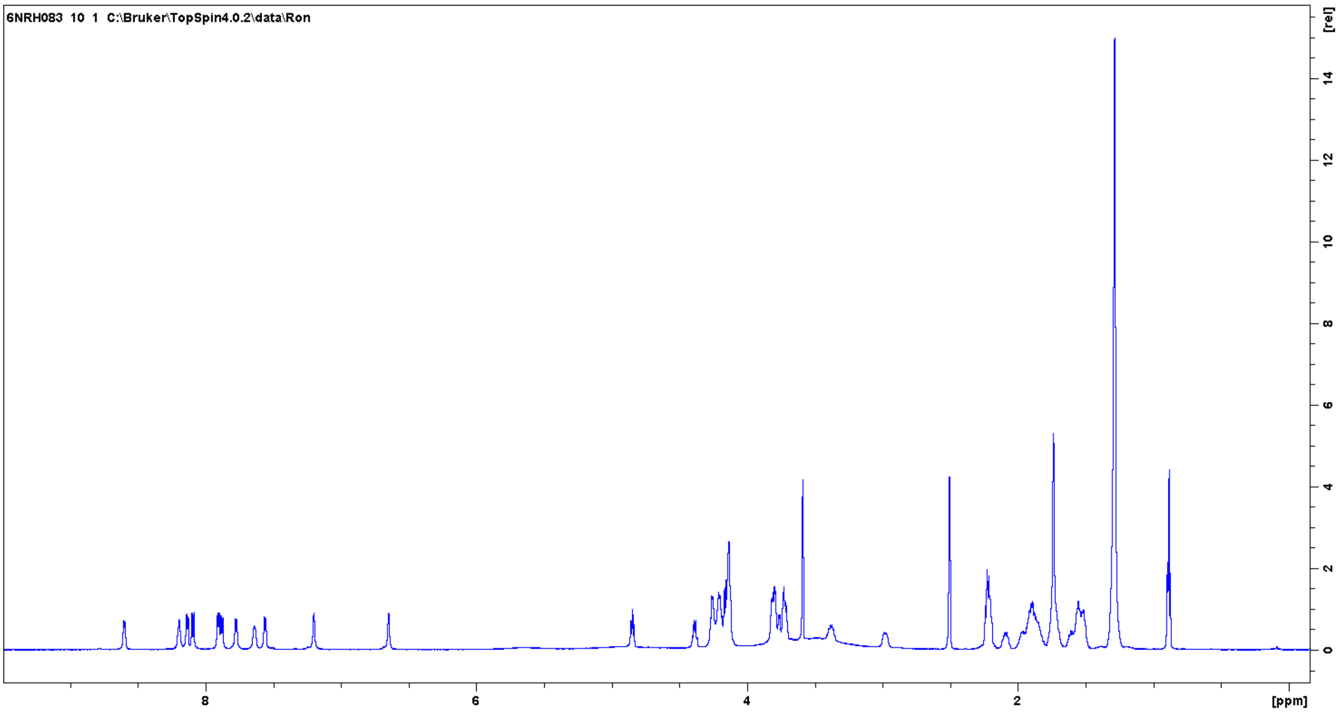

Figure S69. ^{1}H NMR spectrum of plantaribactin (600 MHz, 298K, DMSO-d_6/THF-d_8 (1:3)).

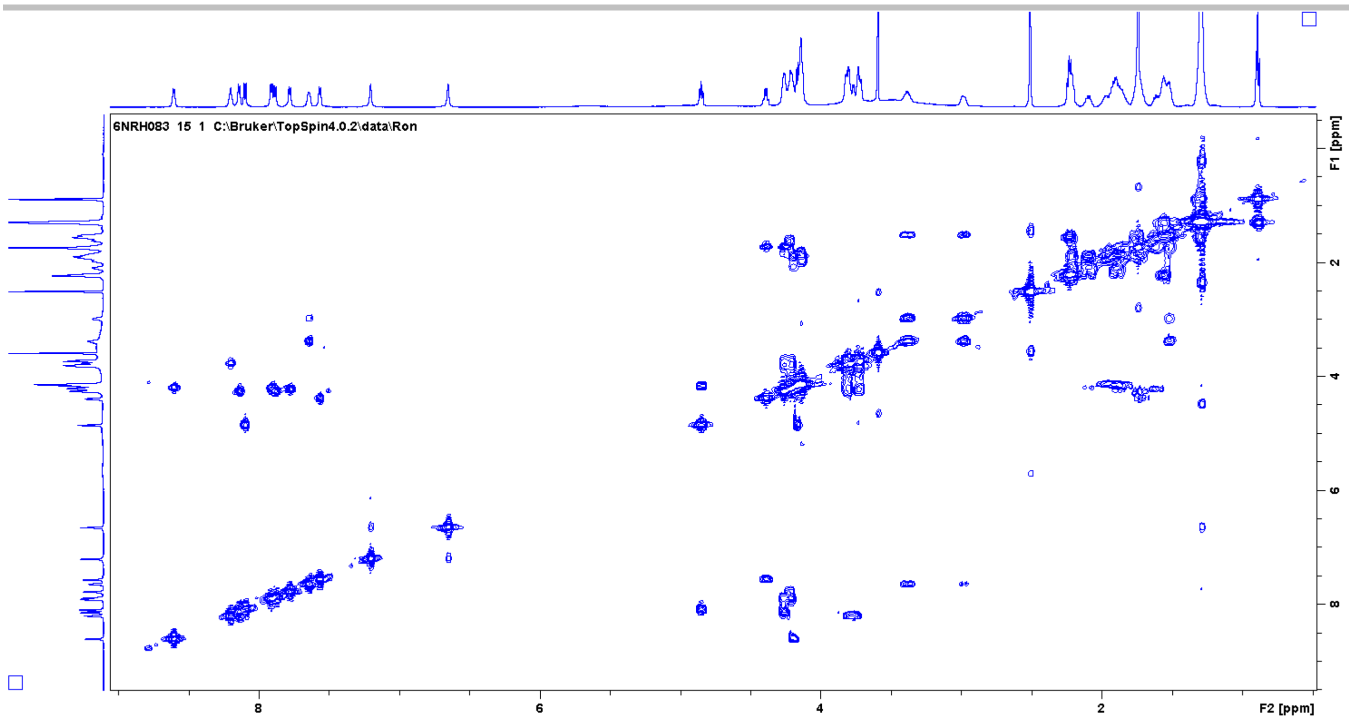

Figure S70. ^{1}H-^{1}H-COSY spectrum of plantaribactin (600 MHz, 298K, DMSO-d_6/THF-d_8 (1:3)).

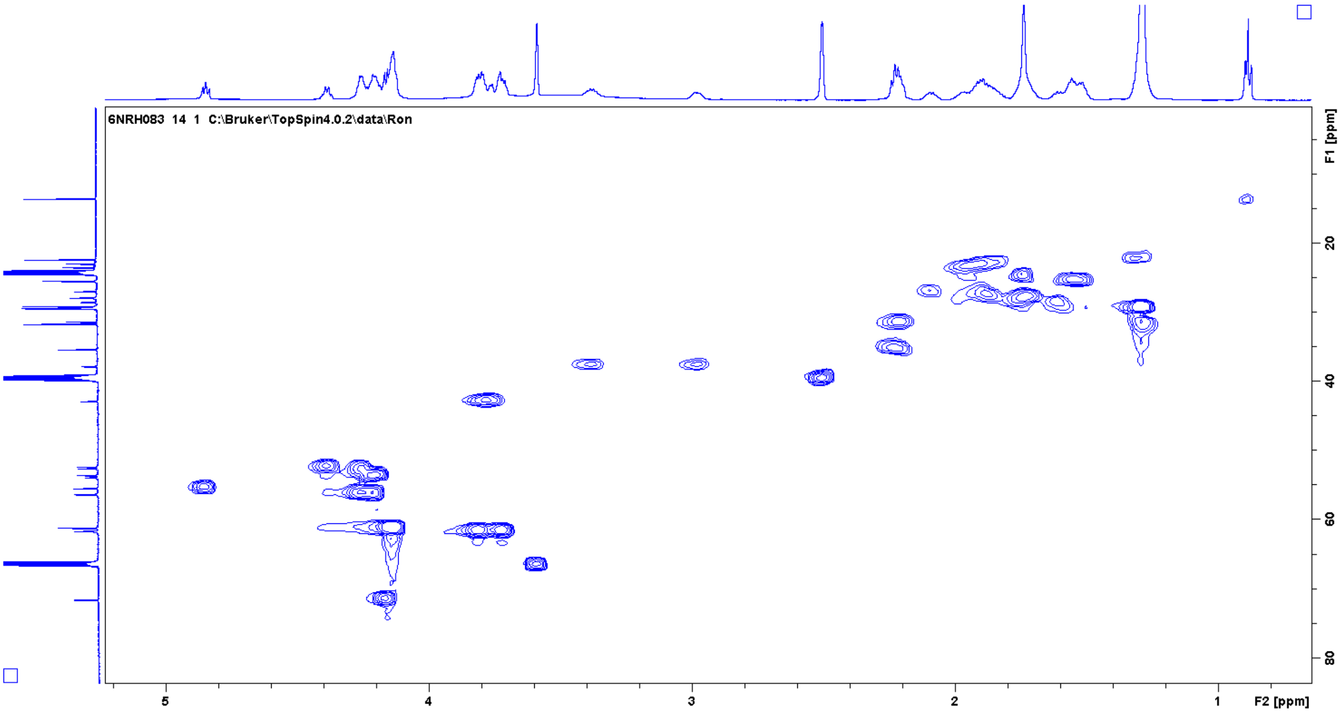

Figure S71. HSQC spectrum of plantaribactin (600 MHz, 298K, DMSO-d_6/THF-d_8 (1:3)).

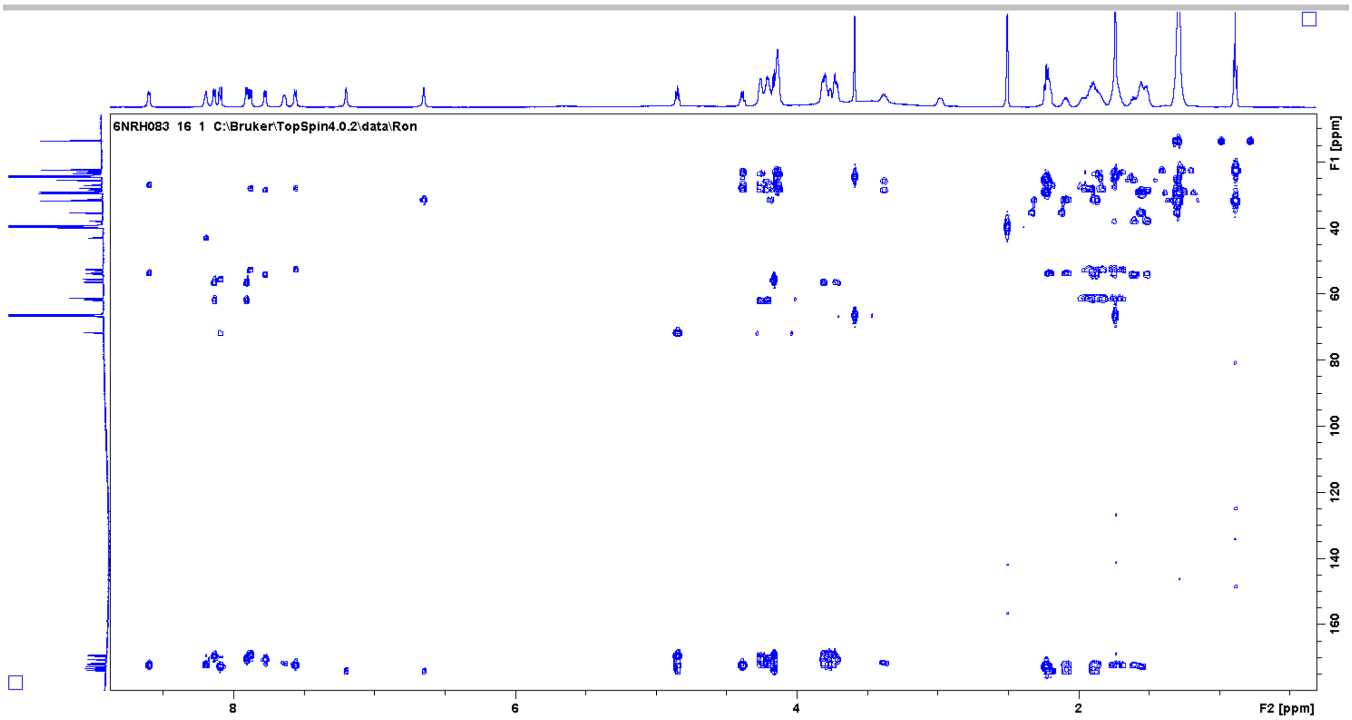

Figure S72. HMBC spectrum of plantaribactin (600 MHz, 298K, DMSO-d_6/THF-d_8 (1:3)).

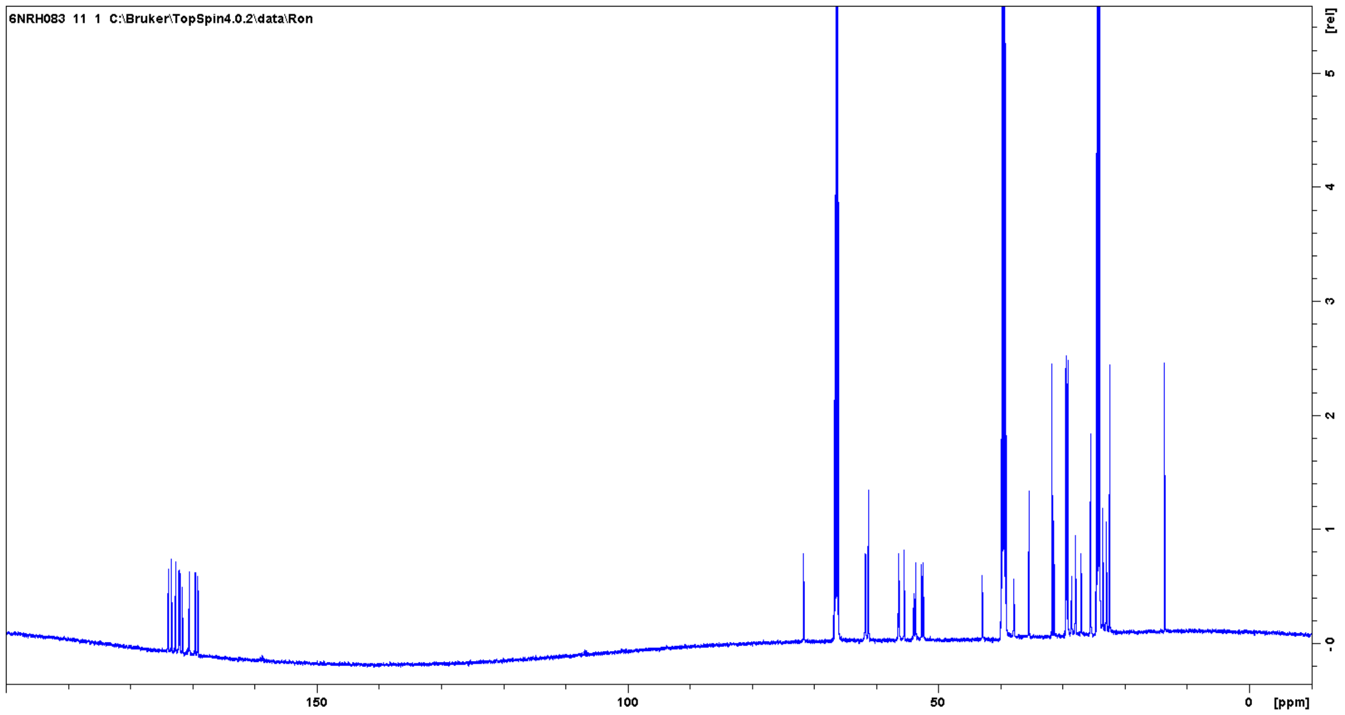

Figure S73. ^{13}C NMR spectrum of plantaribactin (150 MHz, 298K, DMSO-d_6/THF-d_8 (1:3)).

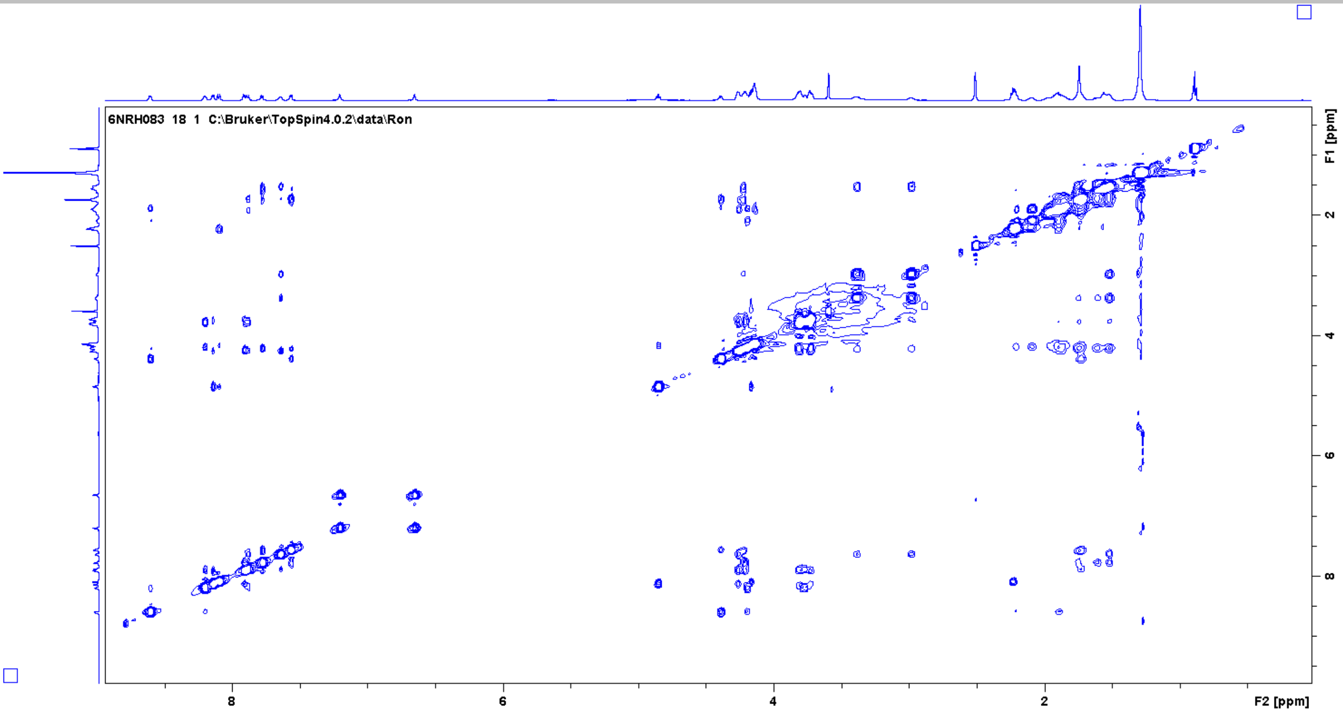

Figure S74. NOESY spectrum of plantaribactin (600 MHz, 298K, DMSO-d_6/THF-d_8 (1:3)).

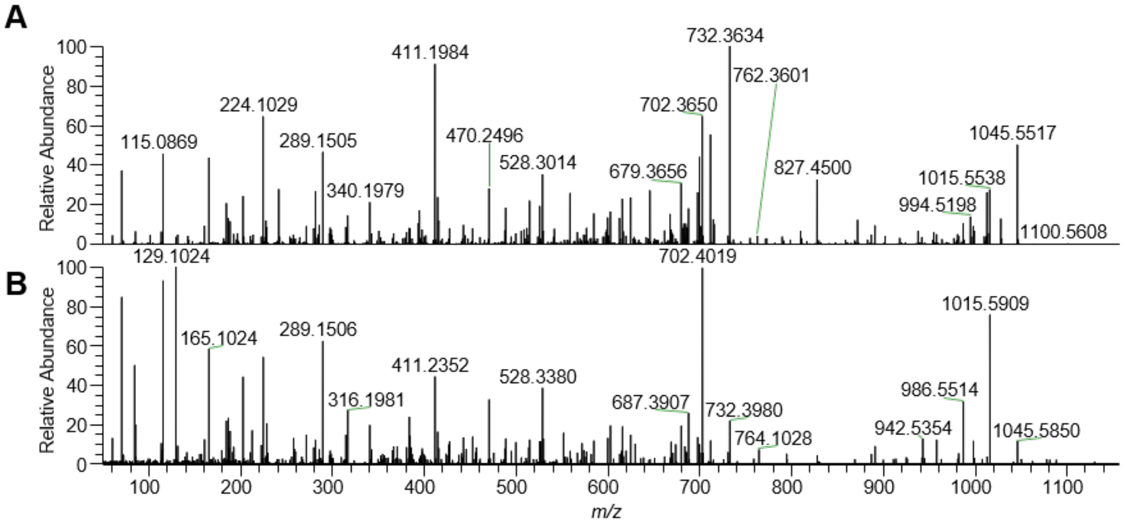

Figure S75. Comparison of MS/MS spectra of plantaribactin (**A**) and gladiobactin (**B**).

References

[1] R. Hermenau, K. Ishida, S. Gama, B. Hoffmann, M. Pfeifer-Leeg, W. Plass, J. F. Mohr, T. Wichard, H.-P. Saluz, C. Hertweck, *Nat. Chem. Biol.* **2018**, *14*, 841-843.
[2] H. L. Buss, A. Lüttge, S. L. Brantley, *Chem. Geol.* **2007**, *240*, 326-342.
[3] K. Ishida, T. Lincke, C. Hertweck, *Angew. Chem. Int. Ed.* **2012**, *51*, 5470-5474.
[4] A. C. Chang, S. N. Cohen, *J. Bacteriol.* **1978**, *134*, 1141-1156.
[5] J. A. Gerlt, J. T. Bouvier, D. B. Davidson, H. J. Imker, B. Sadkhin, D. R. Slater, K. L. Whalen, *Biochim. Biophys. Acta, Proteins Proteomics* **2015**, *1854*, 1019-1037.
[6] G. B. Payne, P. H. Williams, *J. Org. Chem.* **1959**, *24*, 54-55.
[7] C. W. Jones Iii, D. E. Leyden, C. H. Stammer, *Can. J. Chem.* **1969**, *47*, 4363-4366.
[8] K. Fujii, Y. Ikai, T. Mayumi, H. Oka, M. Suzuki, K.-i. Harada, *Anal. Chem.* **1997**, *69*, 3346-3352.
[9] G. Lackner, N. Moebius, C. Hertweck, *ISME J.* **2011**, *5*, 252-261.
[10] S. P. Niehs, B. Dose, K. Scherlach, S. J. Pidot, T. Stinear, C. Hertweck, **2019**, *in preparation*.
[11] V. Viallard, I. Poirier, B. Cournoyer, J. Haurat, S. Wiebkin, K. Ophel-Keller, J. Balandreau, *Int. J. Syst. Evol. Microbiol.* **1998**, *48*, 549-563.
[12] K. Azegami, K. Nishiyama, Y. Watanabe, I. Kadota, A. Ohuchi, C. Fukazawa, *Int. J. Syst. Evol. Microbiol.* **1987**, *37*, 144-152.
[13] T. Coenye, S. Laevens, A. Willems, M. Ohlén, W. Hannant, J. R. Govan, M. Gillis, E. Falsen, P. Vandamme, *Int. J. Syst. Evol. Microbiol.* **2001**, *51*, 1099-1107.
[14] T. Uematsu, D. Yoshimura, K. Nishiyama, T. Ibaragi, H. Fujii, *Jpn. J. Phytopathol.* **1976**, *42*, 464-471.
[15] W. M. Gill, A. L. J. Cole, *Can. J. Microbiol.* **1992**, *38*, 394-397.
[16] T. Coenye, B. Holmes, K. Kersters, J. R. W. Govan, P. Vandamme, *Int. J. Syst. Evol. Microbiol.* **1999**, *49*, 37-42.
[17] L. V. Flórez, K. Scherlach, P. Gaube, C. Ross, E. Sitte, C. Hermes, A. Rodrigues, C. Hertweck, M. Kaltenpoth, *Nat. Commun.* **2017**, *8*, 15172.
[18] K. Kamachi, T. Yamaya, T. Mae, K. Ojima, *Plant Physiol.* **1991**, *96*, 411-417.
[19] F.-X. Felpin, E. Fouquet, *Chem. - Eur. J.* **2010**, *16*, 12440-12445.
[20] E. N. Gate, M. D. Threadgill, M. F. G. Stevens, D. Chubb, L. M. Vickers, S. P. Langdon, J. A. Hickman, A. Gescher, *J. Med. Chem.* **1986**, *29*, 1046-1052.
[21] Y.-M. Lin, M. J. Miller, *J. Org. Chem.* **1999**, *64*, 7451-7458.
[22] B. O. Bachmann, J. Ravel, *Methods Enzymol.* **2009**, *458*, 181-217.
